# Quinone-transporting filaments expand bioenergetic capacity in Gram-positive Bacillota

**DOI:** 10.1038/s41564-026-02450-z

**Published:** 2026-08-17

**Authors:** Ashleigh Kropp, Kazem Asadollahi, Jamie A. Stapleton, Luka Simsive, Pok Man Leung, Rachel L. Darnell, Christopher K. Barlow, Benjamin G. Hartmann, Nicholas D. J. Yates, Tasha Lumbantobing, Daniel R. Fox, Chris Greening, Oleksii Zdorevskyi, Vivek Sharma, James N. Blaza, Alison Parkin, Rhys Grinter

**Affiliations:** 1 Department of Biochemistry and Pharmacology, Bio21 Molecular Science and Biotechnology Institute, The University of Melbourne, Parkville, Victoria Australia; 2 Centre for Electron Microscopy of Membrane Proteins, Monash Institute of Pharmaceutical Sciences, Parkville, Victoria Australia; 3 Department of Microbiology, Biomedicine Discovery Institute, Monash University, Clayton, Victoria Australia; 4 Department of Chemistry, University of York, York, UK; 5 Department of Physics, University of Helsinki, Helsinki, Finland; 6 Centre for Bacterial Cell Biology, Newcastle University Biosciences Institute, Newcastle University, Newcastle-upon-Tyne, UK; 7 Monash Proteomics and Metabolomics Platform, Department of Biochemistry, Monash Biomedicine Discovery Institute, Monash University, Clayton, Victoria Australia; 8 York Structural Biology Laboratory, Department of Chemistry, University of York, York, UK; 9 HiLIFE Institute of Biotechnology, University of Helsinki, Helsinki, Finland

**Keywords:** Bacterial physiology, Cryoelectron microscopy, Enzyme mechanisms

## Abstract

Cellular respiration depends on transferring electrons to hydrophobic quinones in membrane bilayers, meaning bioenergetic capacity is constrained by available membrane surface area. While Gram-negative bacteria expand this capacity through internal membrane invaginations and eukaryotes use membrane-bound organelles, whether Gram-positive bacteria have alternative capacity-generating mechanisms is unknown. Here we show that *Bacillus subtilis* forms a quinone-transporting pseudomembrane composed of filaments of the NADH dehydrogenase Ndh and the quinone-transporting protein Ncp. Cryo-EM, lipidomics and molecular dynamics reveal that Ndh and Ncp co-assemble with lipids into a 4:4 stoichiometric complex with a solvent-excluded hydrophobic lumen containing phospholipids, which sequesters quinones. These complexes further assemble into filaments, linking chambers into a continuous conduit that amplifies quinone reduction. Phylogenetic analysis suggests that this capacity is widespread in Bacillota. Quinone-transporting filaments thus reveal a strategy to expand the quinone pool and bioenergetic capacity while occupying minimal membrane space.

## Abstract (web summary)

*Bacillus subtilis* Ncp and NADH dehydrogenase form tube-like filaments with phospholipid-lined chambers that associate with quinones, which can expand bioenergetic capacity and support respiratory electron transfer.

## Main

Cellular respiration is fundamentally constrained by the surface area available to house electron-transport machinery^1,2^. Overcoming this limitation has been proposed as a central driver of eukaryogenesis, with mitochondria and chloroplasts providing massively expanded internal membranes to support energy conversion^3–5^. Certain Gram-negative bacteria also employ organelles for energy conversion, notably thylakoids, chlorosomes and anammoxosomes^6^. Others use extensive membrane invaginations to boost electron input capacity^3–5,7–10^. Gram-positive bacteria appear to lack internal membranes or organelles that expand their respiratory capacity^11^. In addition, they only have a single membrane, which must accommodate other essential processes such as cell wall synthesis, nutrient import and secretion^11,12^. Because respiratory ATP synthesis depends on electron transfer within this limited membrane surface, mechanisms that expand this capacity would confer an advantage under rapid growth or metabolic stress^1,13^. Central to this process are respiratory quinones, highly hydrophobic molecules that shuttle electrons between respiratory dehydrogenases and terminal oxidases^14^. For respiratory enzymes, accessing membrane-embedded quinones poses a fundamental challenge. Many enzymes solve this problem by using integral membrane subunits, often containing haem or iron–sulfur clusters, that reduce quinones directly within the bilayer, and require permanent membrane real estate^15,16^. Others peripherally associate with the membrane to reach the quinone pool without dedicated transmembrane subunits^17,18^. Another recently discovered strategy involves extracting quinones from the membrane and reducing them in the cytosol using either membrane-associated quinone transport channels or a soluble quinone-binding shuttle protein^19–21^.

Nicotinamide adenine dinucleotide (NADH) is a key intermediate electron carrier in cellular metabolism, transporting low-potential electrons primarily from organic carbon oxidation to the electron-transport chain via the reduction of respiratory quinone^22,23^. While this reaction is classically associated with the multisubunit complex I (type-1 NADH dehydrogenase), many bacteria instead rely on type-2 NADH dehydrogenase (NDH-2). Unlike complex I, NDH-2 is a compact, ~50 kDa flavoprotein that does not span the membrane or pump protons^24–28^. It is often the dominant or sole NADH-oxidizing enzyme in bacteria, underscoring its physiological importance^17,29^.

The soil-dwelling facultatively anaerobic Gram-positive bacterium *Bacillus subtilis* encodes three putative NDH-2 enzymes, *ndh, yumB* and *yutJ*, to transfer electrons from NAD(P)H to the respiratory chain^29,30^. Among these, Ndh is the primary NADH dehydrogenase during vegetative growth^29^. NDH-2 enzymes often peripherally associate with the membrane to transfer electrons to quinone within the bilayer^17,26,31–34^. However, *B. subtilis* Ndh lacks key residues in this region for membrane interaction, raising the question of how it accesses and reduces quinones. The *ndh* gene shares an operon with *yjlC*, which encodes a protein with a C-terminal DUF1641 domain^29^. Recently discovered DUF1641-domain proteins form quinone-transporting complexes with both H_2_- and formate-oxidizing enzymes (Huc and ForCE)^19,20^.

Here we identify that the *yjlC* gene product, which we designate the Ndh-coupling protein (Ncp), forms a stable NADH-oxidizing complex with Ndh. Unlike the Huc and ForCE complexes, the Ndh–Ncp complex assembles into tube-like filaments with internally connected hydrophobic chambers lined by many embedded phospholipids^19,20^. These Ndh–Ncp ‘pseudomembrane’ tubes enable the sequestration and long-range transport of respiratory quinones, hundreds of angstroms outside of the membrane, for reduction by electrons from NADH. By doing this, Ndh–Ncp filaments extend the surface area for quinone reduction, providing a solution to the limitation of membrane real estate available for energy generation, functionally convergent with mitochondrial cristae and respiratory supercomplexes^3,4,35,36^. Genomic analysis shows that Ndh–Ncp complexes are widely distributed across Bacillota, indicating that respiratory chain expansion via protein–lipid pseudomembrane filaments is a conserved and fundamental mechanism in bacterial bioenergetics.

## Results

### Ndh from *B. subtilis* forms a functional complex with Ncp

**Fig. 1:**
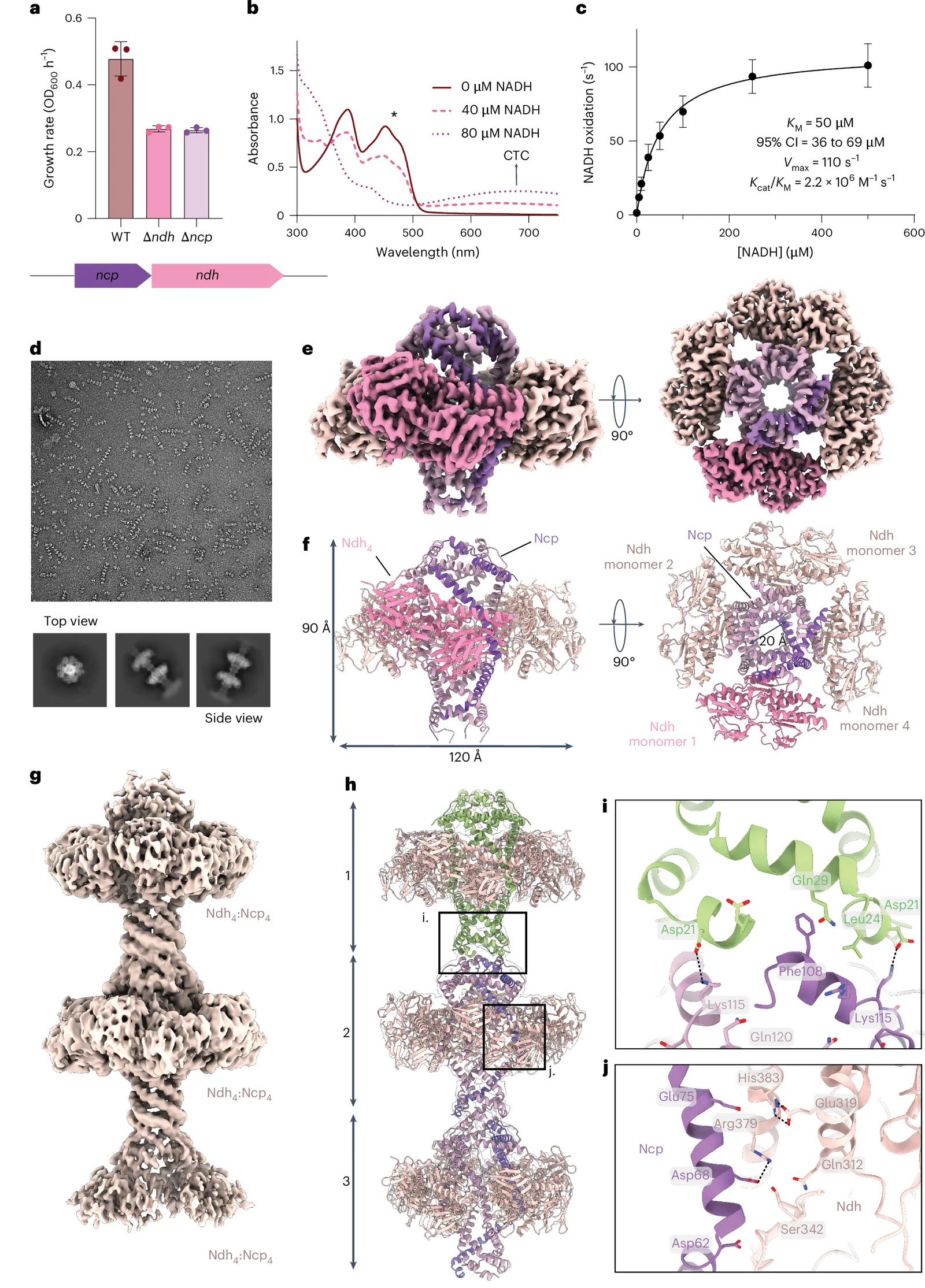
*B. subtilis* Ncp complexes with Ndh and is essential for its activity. **a**, Growth rates of WT, Δ*ndh* or Δ*ncp B. subtilis* 168 strains during exponential phase. Data presented as mean ± s.d. of *n* = 3 biological replicates. **b**, Representative UV–visible spectra of Ndh–Ncp complex with increasing concentrations of NADH. The asterisk (*) shows the typical flavin peak at 450 nm, and the arrow represents the charge-transfer complex (CTC) at ~660 nm. **c**, Michaelis–Menten kinetics of Ndh–Ncp incubated with 500 µM MDA and varying concentrations of NADH. Data presented as mean ± s.d. of *n* = 3 independent replicates. **d**, Representative negative-stain EM micrograph showing filamentous Ndh–Ncp (top, representative micrograph from *n* = 10 replicates) and cryo-EM 2D class averages of filaments (bottom). **e**, Cryo-EM map of the Ndh–Ncp complex showing the Ndh subunits in pale pink and one representative in bright pink, and the Ncp in purple. The images show the side (left) and top (right) views of the complex. **f**, Cartoon representation of the cryo-EM structure of the Ndh–Ncp complex. Four Ndh monomers (light pink) couple to Ncp (purple) with C4 symmetry. Left: side view. Right: top view of the complex. **g**, Cryo-EM map of the Ndh–Ncp helical refinement showing three subunits in the filament**. h**, Ndh–Ncp filament structure showing three units in the filament. A cartoon representation is shown, with the top Ncp tube in green, the following two tubes in purple and the interacting Ndh enzymes in pale pink. **i**, Interaction between the ‘tail’ (green) of Ncp from one unit and the ‘head’ (purple) Ncp from another unit in the filament. Key residues that promote filament formation are highlighted. **j**, Interface between one Ndh enzyme and Ncp monomer, highlighting key residues that drive the interaction. Source data

**Extended Data Fig. 1:**
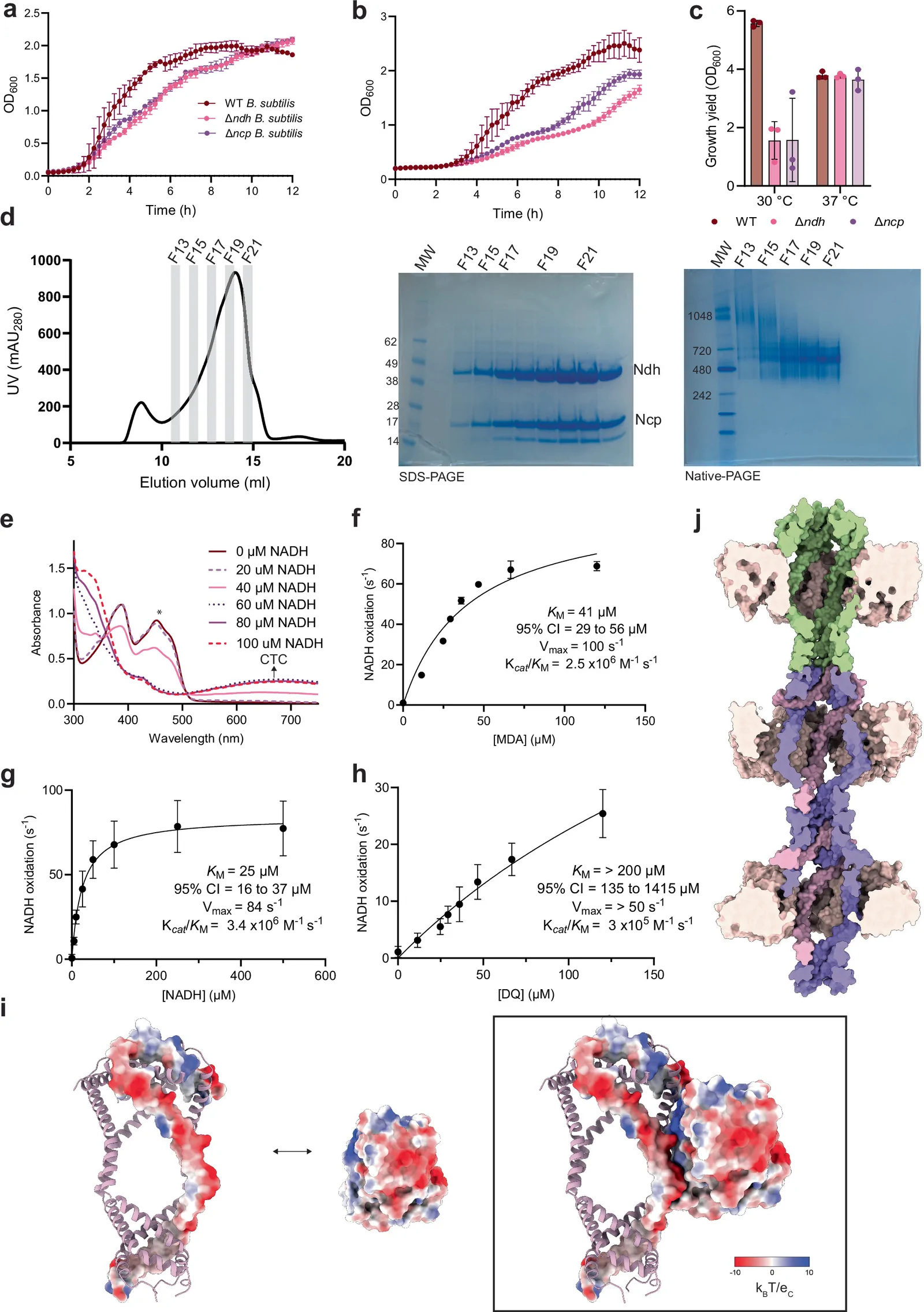
*B. subtilis* Ndh-Ncp purification and characterisation. **a**, A growth curve of WT, Δ*ndh* and Δ*ncp B. subtilis* 168 strain, grown at 37˚C, showing OD_600_ vs. time (h). **b**, A growth curve of WT, Δ*ndh* and Δ*ncp B. subtilis* 168 strain, grown at 30˚C, showing OD_600_ vs. time (h). **c**, The growth yield of WT, Δ*ndh* and Δ*ncp B. subtilis* 168 strain after 24 hours of growth at 30 or 37 ˚C, measured in OD_600_. Data in panels a, b and c are from three or six biological replicates (n = 3, n = 6) with mean ± standard deviation displayed. **d**, The SEC profile (Superose 6 10/300) from the purification of Ndh-Ncp with the corresponding SDS-PAGE and Native^−^PAGE gels. Ndh has an expected size of 42 kDa and Ncp 19.4 kDa (with strep tag). Raw gel images are shown in the Source Data. **e**, Representative UV-Vis spectra of Ndh-Ncp complex with increasing concentrations of NADH (all concentrations shown). **f**, Michaelis-Menten kinetics of Ndh-Ncp incubated with NADH and varying concentrations of MDA. **g**, Michaelis-Menten kinetics of Ndh-Ncp incubated with decylubiquinone (DQ) and varying concentrations of NADH. **h**, Michaelis-Menten kinetics of Ndh-Ncp incubated with NADH and varying concentrations of DQ. **i**, An electrostatic surface representation of the cryo-EM structure of one Ncp and one Ndh overlayed on the cartoon representation of NCP tube showing the interaction interface between one Ncp and one Ndh. Red colouring corresponds to negatively charged residues, white neutral, and blue positively charged residues. **j**, Surface representation of the Ndh-Ncp cryo-EM structure cut down the centre to show the continuous Ncp tube between complexes. Data in panels f, g and h are from three independent replicates (n = 3) with mean ± standard deviation displayed. Source data

The *ndh* gene from *B. subtilis* is important for optimal growth and forms an operon with *ncp* (formerly *yjlC*)^29^. To test whether *ndh* and *ncp* are functionally associated, we generated *B. subtilis* knockout strains lacking either gene (Δ*ndh*::kan or Δ*ncp*::kan) and assessed their growth in liquid culture. Both Δ*ndh*::kan and Δ*ncp*::kan strains exhibited a slower growth rate, with defects of similar magnitude at 37 °C, suggesting that loss of *ncp* phenocopies loss of *ndh* (Fig. 1a and Extended Data Fig. 1a). In addition, when grown at 30 °C (which reduces sporulation of *B. subtilis*), the growth yield of both strains was substantially reduced (Extended Data Fig. 1b,c).

Given the genetic linkage between *ndh* and *ncp*, and the homology of Ncp to HucM, we hypothesized that Ndh and Ncp form a complex that couples Ndh activity to the membrane. To investigate this, we constructed an *Escherichia coli* expression construct encoding both *ncp* and *ndh* genes in tandem, with *ncp* N-terminally twin-strep tagged. Expression and purification from clarified cell lysate resulted in the isolation of a complex containing both Ndh and Ncp, which had an elongated peak on size exclusion chromatography (SEC), with mass photometry indicating a non-uniform size distribution (Extended Data Fig. 1d and Supplementary Fig. 1). UV–visible spectra demonstrated that the purified Ndh–Ncp complex contains flavin in an oxidized state with absorbance peaks at ~390 nm and 450 nm. The addition of NADH led to the formation of a charge-transfer complex (CTC) between NADH and FAD^+^, indicated by an absorbance peak at 660 nm (Fig. 1b and Extended Data Fig. 1e)^26^. This CTC wavelength is comparable to that of NDH-2 from *Caldalkalibacillus thermarum* and *Staphylococcus aureus* but is shorter than the 740 nm of the distantly related eukaryotic NDH-2 from *Saccharomyces cerevisiae*^26,33,37^.

We next measured the catalytic kinetics of Ndh–Ncp using the quinone analogues menadione (MDA) or decylubiquinone (DQ) as electron acceptors. Ndh–Ncp oxidizes NADH with a Michaelis constant (*K*_M_) of 50 µM (confidence interval (CI) 36–69 µM) and a maximum velocity (*V*_max_) of 110 s^−1^, with MDA as the electron acceptor (Fig. 1c and Extended Data Fig. 1f). The complex has lower affinity and is ~10-fold slower than NDH-2 from *C. thermarum* with MDA as an electron acceptor (*K*_M_ = 29 µM, *V*_max_ = 1,190 s^−1^)^26^. Using DQ as an electron acceptor for Ndh–Ncp resulted in a lower *V*_max_, suggesting that Ndh–Ncp prefers menaquinone over ubiquinone (Extended Data Fig. 1g,h). The slower kinetics of *B. subtilis* Ndh–Ncp compared with *C. thermarum* NDH-2 suggest that this complex may restrict the access of soluble quinones to the Ndh electron acceptor sites compared to characterized NDH-2 enzymes.

### Ndh and Ncp form a complex that associates into filaments

**Extended Data Fig. 2:**
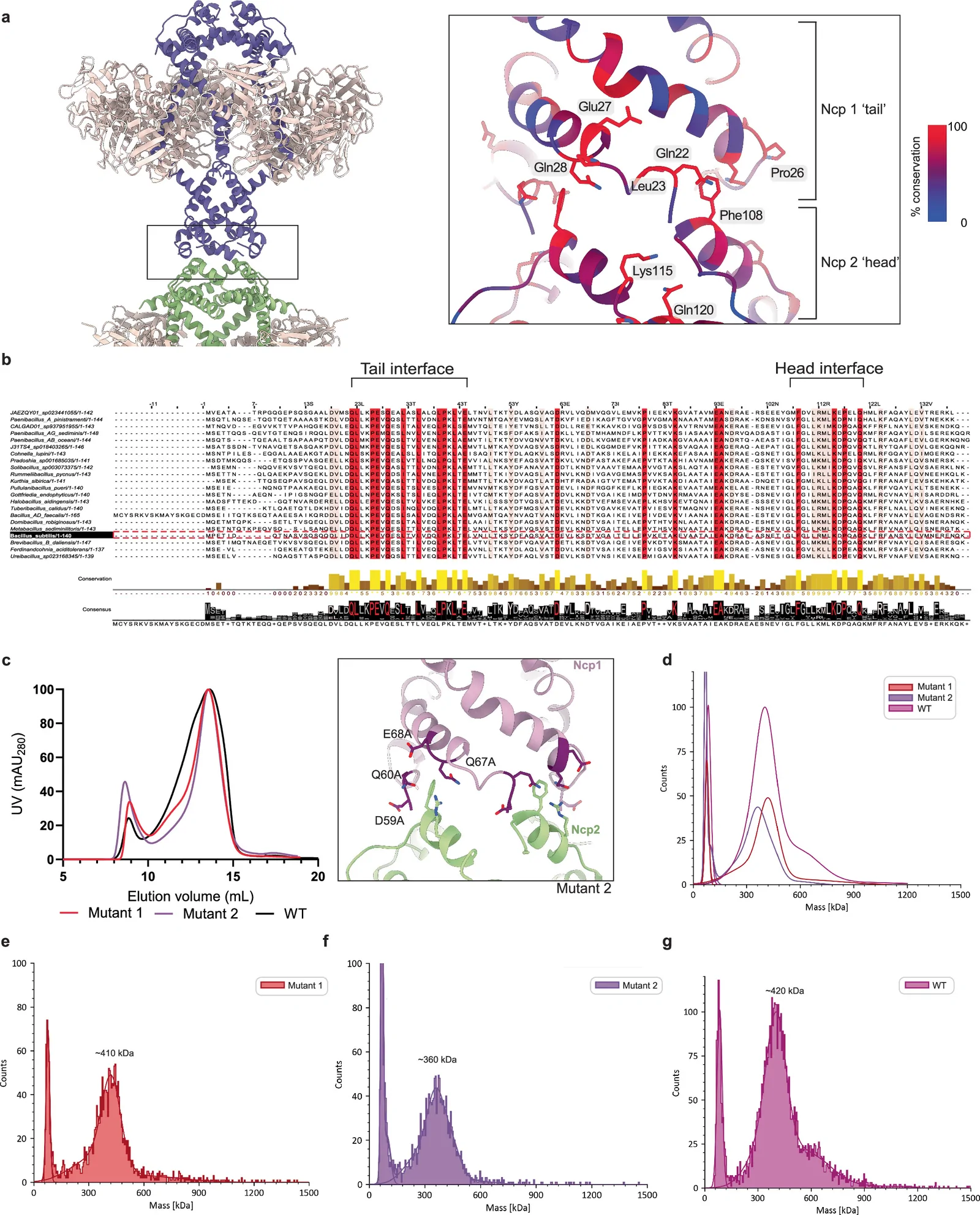
Analysis of the Ndh-Ncp filament interface. **a**, A cartoon view of the Ndh-Ncp complex showing the filament interface (left), with a zoomed view of this region, showing the conservation of amino acids forming key interface contacts (right). **b**, A multiple sequence alignment of selected diverse Ncp homologues showing highly conserved amino acids at the head and tail regions of the filament interface. **c**, SEC chromatograms of purified WT Ndh-Ncp, and Ndh-Ncp with mutations which disrupt the filament interface (mutations are detailed in the methods). **d**, Processed mass photometry data on WT and filament interface Ndh-Ncp mutants, showing mutations cause a loss of higher order oligomerisation. Data were normalised to WT counts. **e-g**, Raw mass photometry counts for curves depicted in panel **d**.

To gain insight into the structure of the Ndh–Ncp complex, we performed negative-stain electron microscopy. Strikingly, these micrographs revealed that purified Ndh–Ncp forms filaments with as many as 13 repeating units (Fig. 1d). To determine the high-resolution structure of this complex, we performed cryo-electron microscopy (cryo-EM) on Ndh–Ncp, followed by single-particle and helical reconstruction (Fig. 1d). We resolved the structure of a single Ndh–Ncp complex at a global resolution of 2.11 Å, revealing a 4:4 stoichiometry of Ndh and Ncp subunits (Supplementary Fig. 2 and Table 1). Ncp forms a hollow tetrameric tube at the centre of the complex, which scaffolds four Ndh subunits. The complex has C4 symmetry, spans ~90 Å in length and ~120 Å in width, and has openings in the Ncp tube of ~20 Å in diameter at its head and tail (Fig. 1e,f). The interface between the Ndh monomers and the Ncp scaffold is primarily driven by electrostatic interactions mediated by a positively charged Ndh C-terminal helix, which is complemented by the negative charge of the Ncp scaffold in this region (Fig. 1j and Extended Data Fig. 1i). To reconstruct the Ndh–Ncp filament, we performed a helical refinement, generating a map with three connected Ndh–Ncp complexes (Fig. 1g,h, Extended Data Fig. 1j, and Supplementary Fig. 2, Video 1 and Table 1). These Ndh–Ncp subunits stack head-to-tail to form the filament, with the connecting interface composed entirely of the Ncp subunit and driven by a phenylalanine (Phe108) on the Ncp head embedding into the tail complex, as well as a salt bridge between Lys115 (head) and Asp21 (tail) (Fig. 1h,i). Sequence analysis of Ncp homologues from diverse Gram-positive bacterial species indicates that key interacting residues on both sides of the filament interface are highly conserved (Extended Data Fig. 2a,b), indicating that natural selection maintains the integrity of this interface, suggesting that it is physiologically relevant. Consistent with this, mutagenesis of key interacting residues of the interface diminished filament formation (Extended Data Fig. 2c–g).

**Extended Data Fig. 3:**
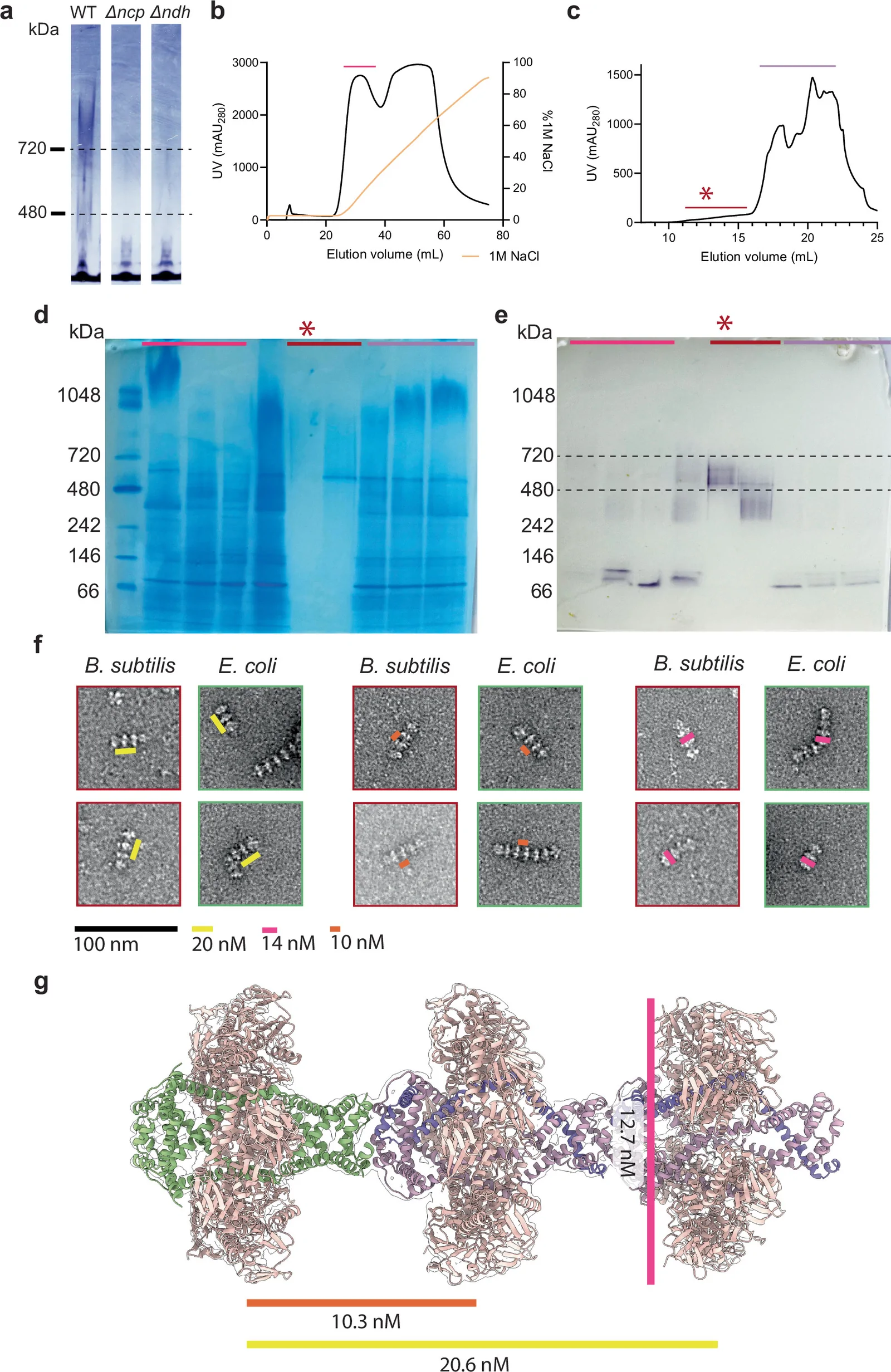
Extraction of Ndh-Ncp filaments from *B. subtilis*. **a**, Native-PAGE of WT, *Δncp* and *Δndh B. subtilis* cell lysates stained for NADH oxidase activity, showing activity attributable to the intact Ndh-Ncp complex ranging between the 480 and 720 kDa size markers. One representative gel shown from n = 3 replicates. **b**, An anion exchange chromatography trace of *B. subtilis* cell lysate eluting over an NaCl gradient. The peak containing strong NADH oxidase activity is marked by the pink line. **c**, A SEC chromatogram of the pooled NADH oxidase activity fractions from panel b. The fraction containing NADH oxidase activity in the size range corresponding to Ndh-Ncp is indicated with a star. **d**, A Coomassie stained Native-PAGE gel of fractions from fractionations shown in panels b and c (n = 1). **e**, NADH oxidase activity staining of a duplicate of the gel from panel d (n = 1). The fraction containing activity at a size attributable to Ndh-Ncp, which was used for negative-stain EM is indicated with a star. **f**, Regions of Negative stain EM images fractionated *B. subtilis* lysates with NADH oxidase activity, showing particles of a size and shape consistent with Ndh-Ncp filaments (left panels), compared to recombinantly produced Ndh-Ncp filaments (right panels) (representative particles from n = 50 micrographs). **g**, A cartoon representation of the Ndh-Ncp filament, showing its dimensions correspond to the particles in panel **f**. Source data

To determine whether Ndh–Ncp filament formation also occurs under native expression conditions in *B. subtilis*, we extracted the complex directly from cells and analysed it using negative-stain EM. To identify NADH-oxidase activity corresponding to Ndh–Ncp, we performed activity staining on lysates from wild-type, *Δndh* and *Δncp* strains. High molecular weight staining was observed exclusively in the wild-type lysate, consistent with the apparent molecular weight of recombinant Ndh–Ncp on native polyacrylamide gel electrophoresis (PAGE) (Extended Data Figs. 1d and 3a). Guided by this activity, we fractionated *B. subtilis* lysates using anion exchange followed by size exclusion chromatography, tracking Ndh–Ncp by native PAGE activity staining (Extended Data Fig. 3b–e). SEC fractions containing NADH-oxidase activity in the expected size range were analysed by negative-stain EM. These fractions contained stacked filaments closely resembling those formed by recombinant Ndh–Ncp, with dimensions consistent with the cryo-EM structure (Extended Data Fig. 3f,g). We additionally confirmed that Ndh–Ncp is strongly enriched in this fraction by mass spectrometry (Supplementary Data 1). This indicates that Ndh–Ncp filaments form under physiological conditions in *B. subtilis*. Notably, this fractionation strategy did not substantially concentrate the lysate, suggesting that filament formation is not an artefact of overexpression or protein concentration.

**Fig. 2:**
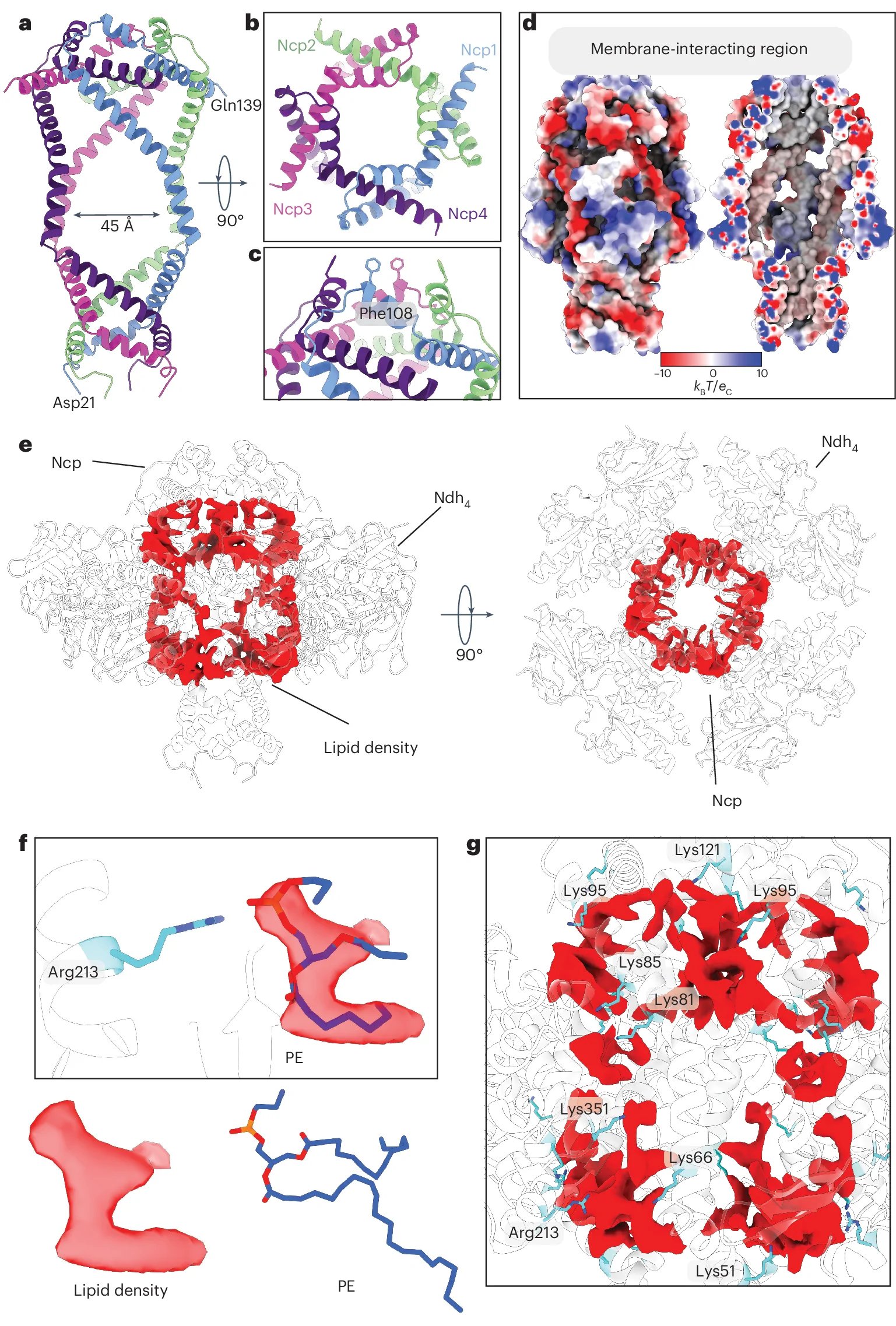
Ncp facilitates membrane interaction and Ndh–Ncp associates with lipids. **a**, Cartoon representation of the structure of Ncp tetramer alone, with each Ncp monomer in a different color. The Ncp tube broadens where Ndh binds, reaching a width of ~45 Å. **b**, The DUF1641 domain forms the opening of the tube and **c**, Phe108 from each monomer facilitates membrane interaction. **d**, The membrane-interacting region of Ncp is characterized by positively charged residues (blue) flanking the hydrophobic Phe108 (white) that inserts into the hydrophobic bilayer. Internally, the Ndh–Ncp complex is hydrophobic (white). Scale of electrostatic surface potential (*k*_B_*T*/*e*_C_) shown on the bar below. **e**, Cartoon representation of the Ndh–Ncp cryo-EM structure in white with grey outline, with density corresponding to phospholipids in red. **f**, Example of a phosphatidylethanolamine (PE) molecule modelled into lipid density from the cryo-EM maps of the Ndh–Ncp complex, with the phosphate head group coordinated by Arg213 from Ndh. **g**, Positively charged residues in blue from the Ndh and Ncp subunits coordinate lipid density (red) throughout the complex.

The width of the Ncp tube varies, reaching ~45 Å in the region where Ndh attaches (Fig. 2a). The C-terminal ‘head’ of the Ncp tetramer contains the DUF1641 domain. This region is structurally similar to HucM^20^, with each Ncp subunit travelling up and over the monomer immediately left of it, then under the monomer immediately right of it, stabilizing the complex and creating the opening at the head of the tube (Fig. 2b,c). The equivalent region in Huc mediates membrane interaction via positively charged residues and a bilayer-interacting tryptophan^20^. Consistent with this, the Ncp head is positively charged, with Phe108 positioned to embed in the bilayer (Fig. 2c,d). Interestingly, this region is also responsible for filament formation, suggesting a dual role in lipid and protein interactions, reminiscent of the recently characterized CODH–CoxG complex^21^. Considering that Ndh–Ncp forms filaments and that the complex is purified from the soluble fraction when expressed recombinantly, interaction between the Ncp and the membrane is most probably dynamic in nature or specific to native host membranes.

The Ndh–Ncp filament consists of a tube formed by the head and tail region of the Ncp tetramer, which connects a broader chamber where Ndh associates with the complex. Both the tube and chamber are lined entirely with hydrophobic amino acids, forming a continuous conduit for hydrophobic quinones from the point of membrane attachment to the electron acceptor sites of Ndh (Fig. 2d). Strikingly, additional density is present in the cryo-EM maps in the region of Ndh attachment, filling gaps between the Ndh and Ncp polypeptide chains (Fig. 2e and Supplementary Video 2). The shape and chemical environment of this density are consistent with it belonging to phospholipids. The solvent-facing portion of these densities, which probably corresponds to negatively charged phosphate groups of phosphatidylglycerol (PG) and phosphatidylethanolamine (PE) lipids, is coordinated by complementary positively charged residues (lysine residues 51, 66, 81, 85, 95 and 121 from Ncp and Arg213 and Lys351 from Ndh), and density probably corresponding to aliphatic tails extends into the hydrophobic interior of the Ndh–Ncp complex (Fig. 2e–g). The presence of this density suggests that the Ndh–Ncp complex incorporates a substantial quantity of phospholipids as an additional structural and functional element. Overall, the structure of the Ndh–Ncp complex reveals similarities to Huc and ForCE, indicating a conserved role for the DUF1641 superfamily in enzyme scaffolding, membrane attachment and long-range quinone transport^19,20^. However, filament formation by Ndh–Ncp and the incorporation of phospholipids into the structure indicate considerable structural and mechanistic differences and demonstrate pronounced structural diversity within the DUF1641 superfamily.

### Ndh–Ncp binds NADH and menaquinone in conserved binding sites

**Fig. 3:**
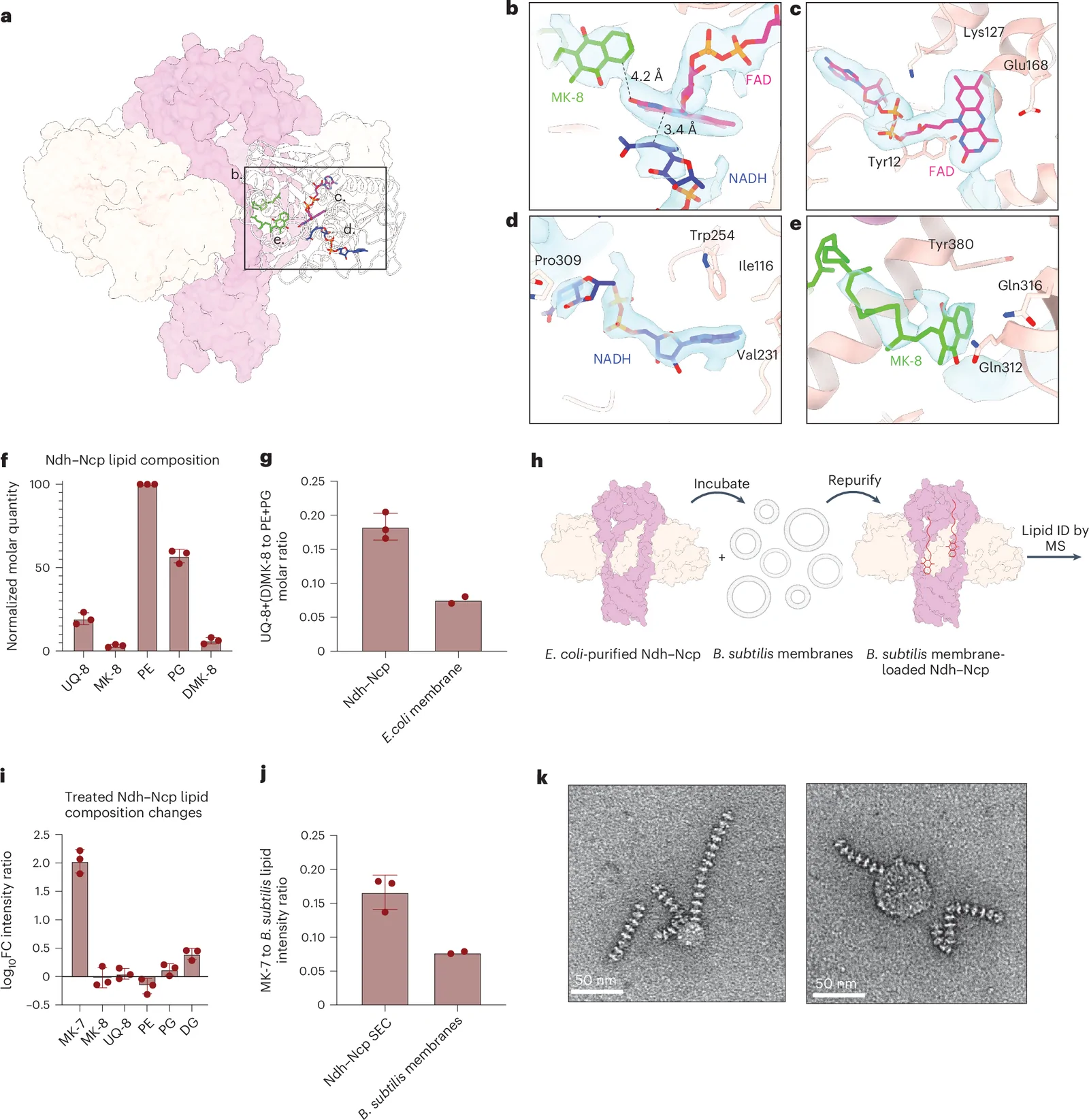
Ndh–Ncp binds NADH and MK-8. **a**, Cartoon representation of the cryo-EM structure of Ndh–Ncp with NADH and quinone bound, showing their location in one Ndh enzyme. **b**, Interatomic distances between each of the ligands; NADH in blue, FAD in pink and MK-8 in green. **c**, Coordination of FAD is driven by conserved Lys127, Glu168, Lys373 and Tyr12. **d**, NADH has a conserved binding mode. **e**, MK-8 is coordinated by Tyr380, Ala311, Gln312 and Gln316. Density for all three cofactors is shown overlaid on the structure, contoured at 5*σ*. **f**, Graph showing the molar quantity of lipids in the Ndh–Ncp purified sample, normalized to the most abundant lipid, PE. Data were from positive ion mode MS, and each type of lipid was summed into its associated group (that is, PE lipids of differing lengths were summed into the PE group). **g**, Molar ratio of UQ-8, MK-8 and DMK-8 to PE and PG in purified Ndh–Ncp complex compared to membranes isolated from the *E. coli* expression strain. **h**, Schematic of the experimental protocol for the incubation of Ndh–Ncp with *B. subtilis* membranes. **i**, Graph showing the fold change (FC) in lipids and quinones after treatment. **j**, Relative MS peak intensity ratio of MK-7 to lipids unique to *B. subtilis* membranes in Ndh–Ncp complex reisolated after incubation with *B. subtilis* membranes compared to the membranes used for the incubation. **k**, Negative-stain EM of streptactin-isolated Ndh–Ncp after incubation with *B. subtilis* membranes, showing attachment to membrane vesicles (representative micrograph from *n* = 20). PE, phosphatidylethanolamine; PG, phosphatidylglycerol; UQ-8, ubiquinone-8; MK-8, menaquinone-8; MK-7, menaquinone-7; DMK-8, demethylmenaquinone-8; DG, diglyceride; PC, phosphatidylcholine. Lipidomics data (**f**,**g**,**j**,**i**) were derived from 3 independent experiments (*n* = 3) for Ndh–Ncp complexes and two independent experiments (*n* = 2) for *E. coli* and *B. subtilis* membranes, with all samples analysed concurrently. Data represent mean ± s.d. Panel **h** created in BioRender: Kropp, A. https://biorender.com/udvq7pg (2026). Source data

Our initial cryo-EM data were collected on Ndh–Ncp in the absence of NADH. This structure had no NADH or quinone bound at their respective binding sites. To obtain the substrate-bound structure, we repeated the cryo-EM, supplementing Ndh–Ncp with 5× the molar concentration of NADH immediately before freezing. We resolved this complex at a global resolution of 2.52 Å, showing clear density for the FAD group, NADH and quinone, at their structurally conserved binding sites (Fig. 3a–e, and Supplementary Fig. 3, Video 3 and Table 1). FAD coordination by Ndh is comparable to that by other structurally characterized NDH-2 enzymes, with isoalloxazine stabilization from Lys127, Glu168 and Lys373 side-chain residues and Ala311 and Gln312 main-chain nitrogens^26,28,31–34^. The pyrophosphate groups engage in hydrogen bonding with the Tyr12, Gly13 and Asp295 main-chain nitrogens, and the adenine ring interacts with the Val80, Gln261 and Lys37 main-chain nitrogens (Fig. 3b,c)^26,31–34^. Ndh binds NADH in the conserved region, interacting with the key WxxG motif (Trp254 and Gly257), as well as with the highly conserved residues Ile116, Gly163, Val196–Gly199, Val231, Pro309 and Thr310 (Fig. 3d)^26,31–34^.

In our NADH-bound structure, we observed density at the conserved quinone-binding electron acceptor site^31,32,34^. No exogenous quinone was added to the Ndh–Ncp complex, meaning that the observed quinone was probably extracted during *E. coli* expression and retained through protein purification. The absence of this density in the NADH-free structure may indicate that quinone binding is preceded by NADH binding. *E. coli*, the expression host for the Ndh–Ncp complex, produces ubiquinone-8 (UQ-8) and menaquinone-8 (MK-8) as its major quinones during aerobic and anaerobic conditions, respectively^14^. The shape and coordination environment of the quinone headgroup density in our structure is more consistent with MK-8, so we modelled this in our structure (Fig. 3e and Supplementary Fig. 4a,b). This is also supported by the superior kinetics for Ndh–Ncp with MDA versus DQ as the electron acceptor (Extended Data Fig. 1f,h)^38^. The Ndh quinone binding site is conserved in other NDH-2 homologues, specifically the motif (AQxAxQ; Ala311, Gln312, Ala314, Gln316)^26,31,34^. The MK-8 binding site faces the interior of the Ndh–Ncp tube, indicating that quinone enters the Ndh electron acceptor site from the internal hydrophobic chamber of the complex (Fig. 3e and Supplementary Fig. 4c,d). Considering this, the slower kinetics of Ndh–Ncp may result from restricted access to the electron acceptor site, due to the tube slowing the movement of the soluble quinones used in this assay (Fig. 1c).

### The Ndh–Ncp complex binds phospholipids and extracts menaquinone

Our cryo-EM structure suggests that phospholipids form an important component of the Ndh–Ncp complex and that the hydrophobic chamber accommodates respiratory quinones. To identify lipids associated with the complex, we performed lipidomics on the purified complex. This analysis revealed a substantial lipid content with a considerable quantity of PE and PG lipid species (Fig. 3f, and Supplementary Data 2 and 3). These data support our interpretation of the cryo-EM density as PE and PG phospholipids. These lipids are probably derived from the *E. coli* plasma membrane and associate with the complex during or after assembly. The high lipid content of the tubular assembly suggests a protein–lipid hybrid architecture capable of extending membrane-like environments beyond the bilayer.

In addition to phospholipids, abundant features with masses and elution profiles corresponding to UQ-8, MK-8 and demethylmenaquinone-8 (DMK-8) were associated with the complex (Fig. 3f, and Supplementary Data 2 and 3). The presence of MK-8 supports its assignment at the electron-acceptor site in the NADH-bound structure. UQ-8 was the most abundant quinone associated with the complex (Fig. 3f); however, as *B. subtilis* primarily utilizes MK-7, this probably reflects the *E. coli* expression system rather than substrate preference. The presence of both ubiquinone and menaquinone in the complex suggests that association is driven primarily by interactions with their hydrophobic isoprenyl tails, which can be accommodated within the central cavity. To quantify quinone enrichment, we compared standardized lipidomics profiles of the Ndh–Ncp complex with those of *E. coli* membranes, calculating the molar ratio of total quinones (UQ and MK species) to total PE and PG lipids. This ratio was higher in the Ndh–Ncp complex, indicating that the complex concentrates quinones relative to the membrane (Fig. 3g). Given this enrichment, quinones can probably occupy both the canonical Ndh binding sites and the central cavity, although additional quinones could not be resolved structurally due to the dynamic nature of this hydrophobic environment.

**Extended Data Fig. 4:**
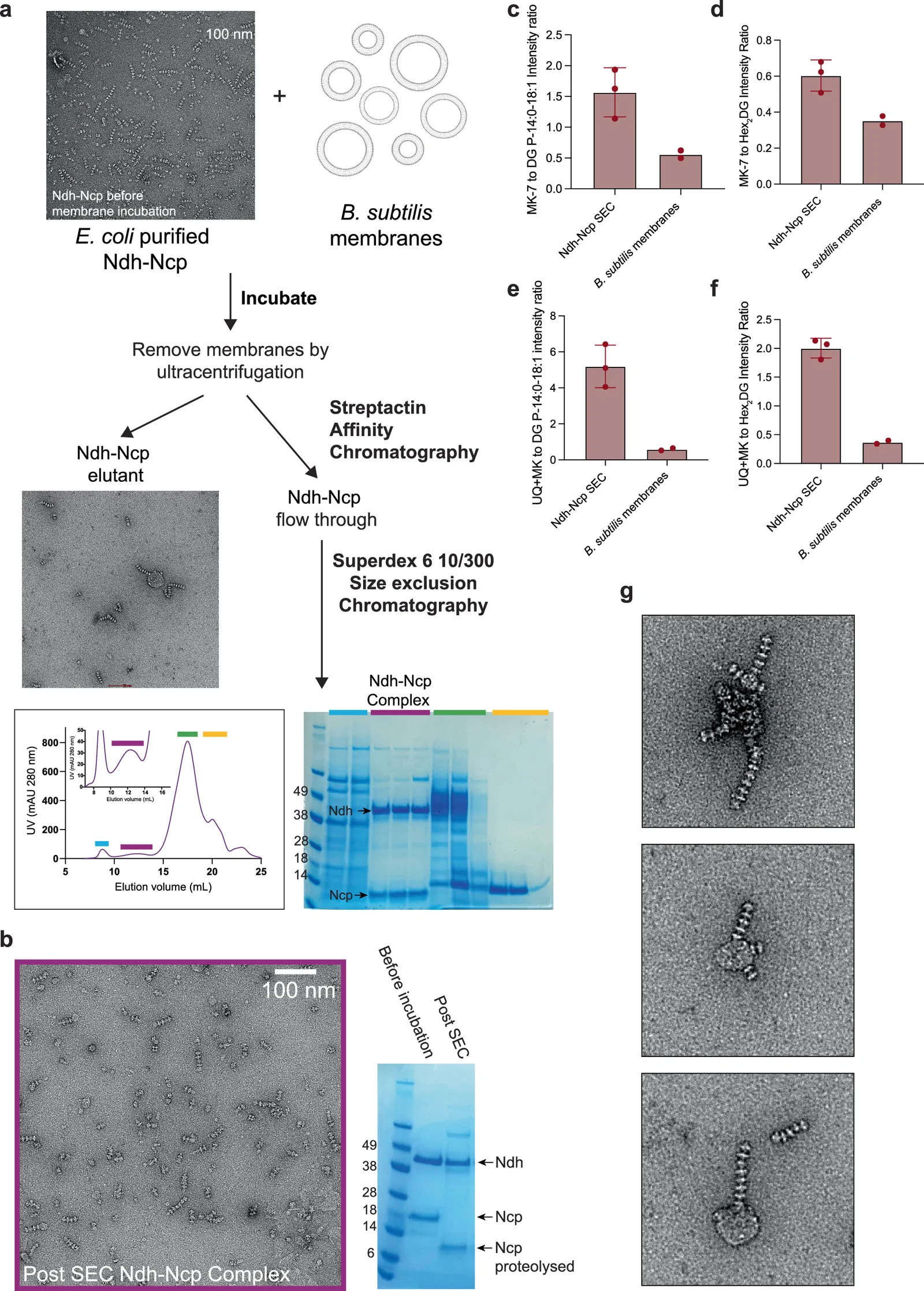
Analysis of recombinant Ndh-Ncp incubated with *B. subtilis* membranes. **a**, A scheme of the experimental workflow for the incubation and re-isolation of recombinantly produced Ndh-Ncp with *B. subtilis* membranes (one representative micrograph is shown from n = 10 (top left) or n = 20 (bottom left) micrographs). Aspects of this figure were generated with BioRender. **b**, Negative stain EM and SDS-PAGE of the reisolated Ndh-Ncp complex utilised for lipidomic analysis. Data from one representative experiment is shown, with three independent experiments conducted to provide material for analysis (one representative micrograph shown from n = 10 micrographs). **c**, The relative mass spec peak intensity ratio of MK-7 to a putatively identified DG P-14-18:1 species in Ndh-Ncp complex reisolated after incubation with *B. subtilis* membranes compared to the membranes used for incubation. **d**. The relative mass spec peak intensity ratio of MK-7 to putatively identified dihexosyldiacylglycerol (Hex_2_DG) species in Ndh-Ncp complex reisolated after incubation with *B. subtilis* membranes compared to the membranes used for incubation. **e**, The relative mass spec peak intensity ratio of all UQ and MK assigned species to DG P-14-18:1 in Ndh-Ncp complex reisolated after incubation with *B. subtilis* membranes compared to the membranes used for the incubation. **f**, The relative mass spec peak intensity ratio of all UQ and MK assigned species to Hex_2_DG species in Ndh-Ncp complex reisolated after incubation with *B. subtilis* membranes compared to the membranes used for the incubation. **g**, Additional negative-stain EM images of Streptactin isolated Ndh-Ncp after incubation with *B. subtilis* membranes, showing attachment to membrane vesicles (one representative micrograph shown from n = 20 micrographs). Lipidomics data in panels **c,d,e** and **f**, are derived from three independent experiments (n = 3) for Ndh-Ncp complexes and two independent experiments (n = 2) for *B. subtilis* membrane, with all samples analysed concurrently. Error bars represent ± SD of the mean. Source data

To assess whether the Ndh–Ncp complex can extract quinones in a native context, we incubated the purified complex with isolated *B. subtilis* membranes, then repurified it and analysed its lipid content (Fig. 3h, Supplementary Note 1 and Extended Data Fig. 4a). *B. subtilis* produces MK-7 as its sole respiratory quinone, and accordingly, MK-7 was the only quinone species identified with increased abundance^38^, with levels increased by ~90-fold relative to pre-incubation (Fig. 3i, and Supplementary Fig. 4 and Data 3)^38^.

To assess enrichment of MK-7 in membrane-incubated Ndh–Ncp relative to the membrane, we compared the within-sample peak intensity ratio of MK-7 to a set of *B. subtilis*-specific lipid species in the post-incubation complex and *B. subtilis* membranes (Supplementary Data 3). The ratio was higher in the Ndh–Ncp sample, indicating increased relative abundance of MK-7 in the complex (Fig. 3j and Extended Data Fig. 4c–f). Similarly, the ratio of total quinone signal to *B. subtilis*-specific lipids was elevated in the complex, suggesting that MK-7 accumulation is not simply due to exchange with pre-bound quinones but reflects net enrichment (Extended Data Fig. 4d,e). Although these ratios were derived from raw peak intensities and may be affected by matrix effects, consistent enrichment across multiple lipid species supports a qualitative conclusion of MK-7 enrichment following membrane incubation.

### The architecture of the Ndh–Ncp–phospholipid complex

**Fig. 4:**
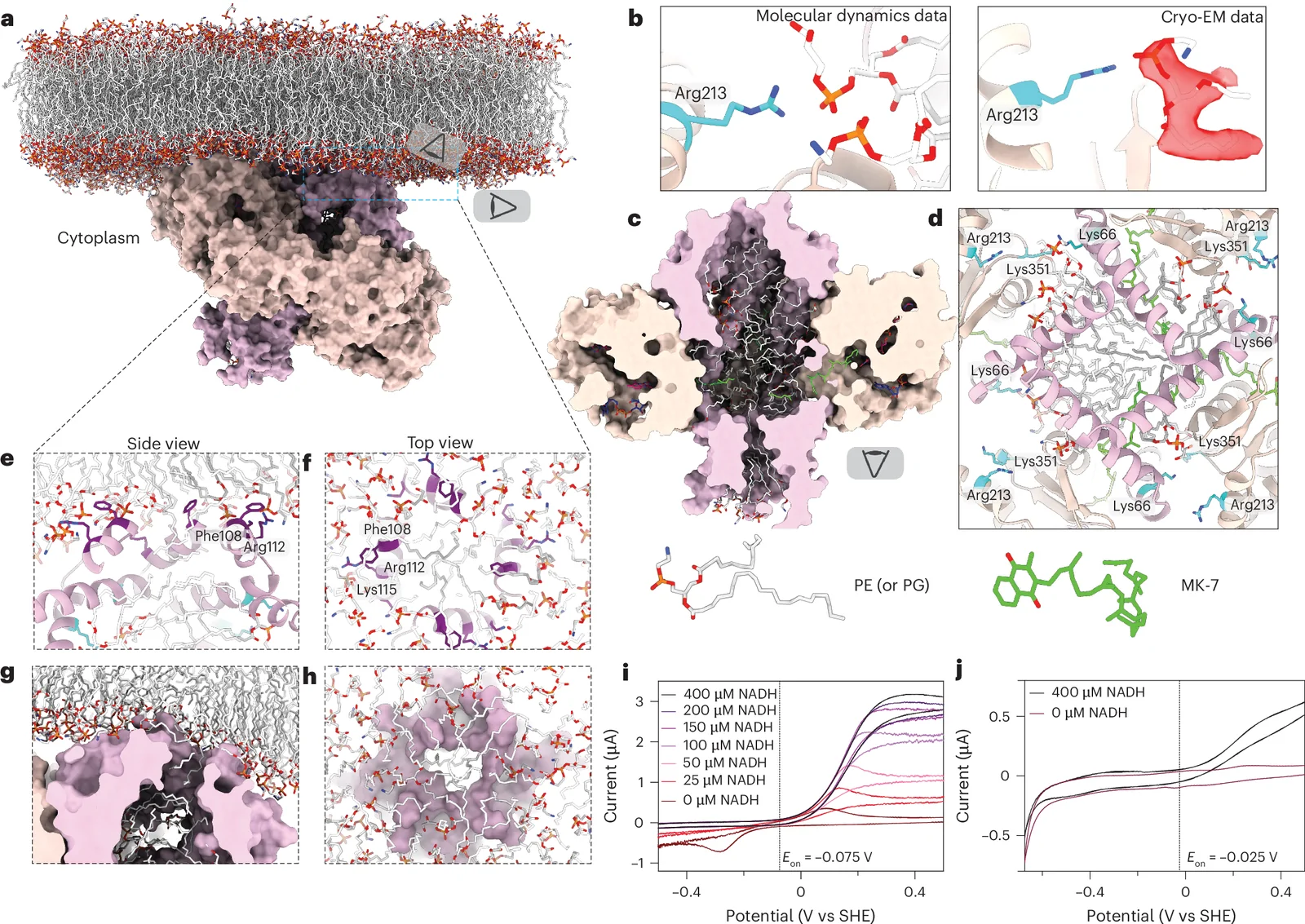
Molecular dynamics simulations demonstrate the Ndh–Ncp protein–lipid architecture. **a**, Ndh–Ncp simulation was set up with a bilayer consisting of PE and PG lipids. Lipids and MK-7 were docked inside the Ncp on the basis of structural and lipidomics data. **b**, A PE and PG molecule from an MD snapshot showing the PG coordinated by Arg213 (left) and cryo-EM data showing a PE molecule in the cryo-EM density coordinated by Arg213 (right). **c**, Snapshot of the last frame of the simulation with PE/PG present within Ndh–Ncp. Cutaway shows the occupation of these molecules within the Ncp and quinone binding site. MK-7 is shown in green and PE/PG in white. **d**, Snapshot of the last frame of the simulation showing the position of phospholipid headgroups in proximity to positively charged residues predicted to coordinate the lipid headgroups (lipids in white, coordinating residues in cyan). The complex is viewed from the bottom. **e**, Side view and **f**, top view snapshot from the last frame of the MD simulation of the Ncp interacting with the bilayer. Key residues Phe108, Arg112 and Lys115 are shown in dark purple. **g**, Side view and **h**, top view of the last frame from MD data of the Ncp tube interacting with the bilayer. Lipids from the bilayer enter the Ncp with the hydrophobic tail directed into the Ncp tube. **i**, Representative cyclic voltammograms of Ndh–Ncp stabilized on a phospholipid membrane recorded at varying NADH concentrations from −0.675 to +0.625 V vs SHE at 10 mV s^−1^ in the presence of MK-7. **j**, Representative cyclic voltammograms of Ndh–Ncp recorded at 0 µM NADH (pink) or 400 µM NADH (black) from −0.675 to +0.625 V vs SHE at 10 mV s^−1^ in the absence of exogenous MK-7. The onset potential (*E*_on_) for each experiment is noted on the voltammograms in **i** and **j**. Source data

**Extended Data Fig. 5:**
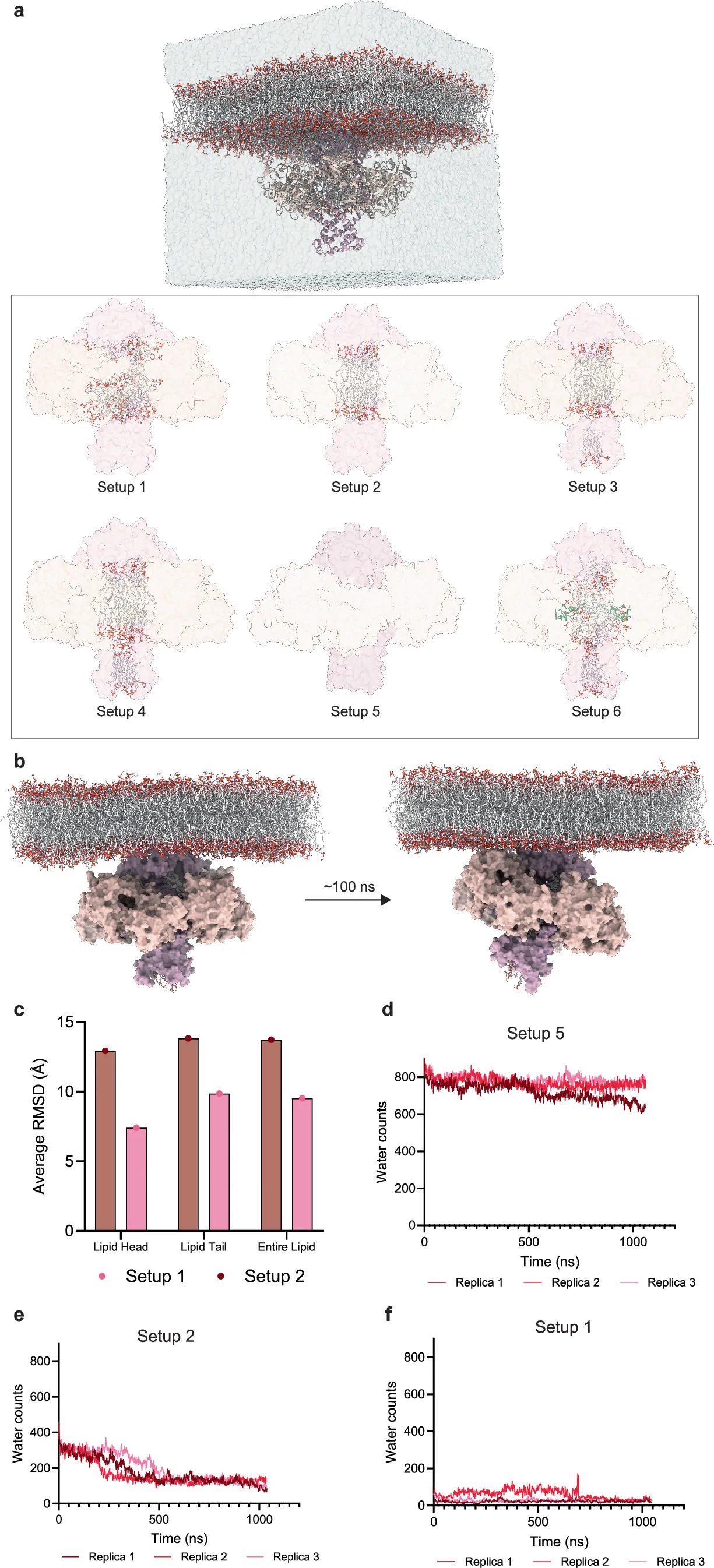
Molecular dynamic simulation setups. **a**, Model systems constructed in this work. Bottom inset shows the initial lipid arrangement inside the Ncp tube for every simulation setup (Table S3), showing the lipids (white) and MK-7 (green). **b**, Tilting of the Ndh-Ncp complex over 100 ns of the simulation trajectory. **c**, The average RMSD (Å) of lipid movement across the simulations in setups 1 and 2, showing lipids are more mobile in setup 2. **d-f**, The number of water molecules within the Ncp chamber over the course of the simulations for setup 5 (no lipids) and setups 1 and 2. Source data

Our structural and lipidomic analysis indicates that Ndh–Ncp forms a hybrid protein–phospholipid complex. However, the density in our cryo-EM maps is insufficiently resolved to model the location of phospholipids in this complex. To resolve this, we performed molecular dynamics (MD) simulations of Ndh–Ncp in complex with PE and PG and associated with a PE/PG lipid bilayer. Due to the size and complexity of the system, we performed extensive optimization simulations to determine optimal lipid numbers and starting positions (Supplementary Note 2, Extended Data Fig. 5a and Supplementary Table 3). The Ndh–Ncp chamber is considerably larger than the Huc or ForCE complexes^19,20^, allowing it to accommodate 39 phospholipids (Supplementary Table 3). In this model, the charged phosphate-containing head groups of PE and PG occupy gaps in the complex between the Ndh and Ncp subunits, facing towards the external solvated environment (Fig. 4a,c,d). The phospholipid headgroup positioning and coordination correlate well with our cryo-EM data, indicating that the MD simulations provide a realistic model of the complex (Fig. 4b). The aliphatic lipid tails extend into and occupy the internal chamber of the complex (Fig. 4c,d). The lipid tails are dynamic throughout the simulations, which is consistent with the poor density in our cryo-EM maps and the role of the complex in long-range quinone transport, which requires lipid mobility within the chamber.

**Extended Data Fig. 6:**
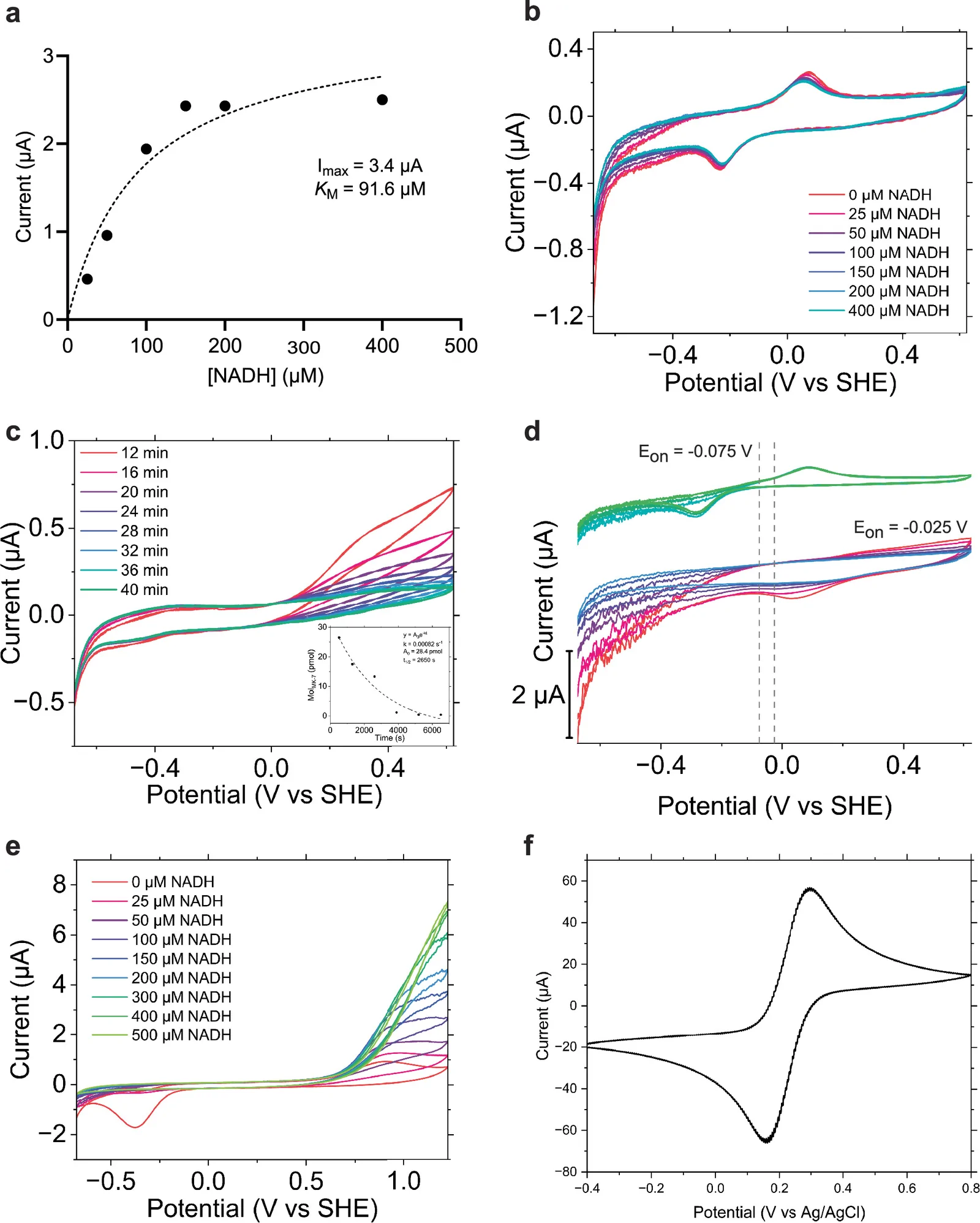
Cyclic voltammetry of Ndh-Ncp. **a**, A plot of the current values at +0.3 V vs SHE were extracted from the reductive sweeps (from Fig. 4i) and plotted against NADH concentration (solid circles), then fit to the Michaelis-Menten equation (dashed line). **b**, Cyclic voltammograms of NADH titration in the absence of Ndh-Ncp, with MK-7 present **c**, Cyclic voltammograms of Ndh-Ncp associated with the bilayer-coated electrode, recorded at 400 μM NADH from -0.675 to +0.625 V vs SHE at 10 mVs^−1^ in the absence of MK-7 at 4-minute intervals, inset of the quinone decay plot calculated from the reductive peak of Extended Data Fig. 6d (bottom). **d**, Cyclic voltammograms of Ndh-Ncp associated with the bilayer-coated electrode in the absence of NADH, in the presence (top) and absence (bottom) of exogenous MK-7. **e**, Cyclic voltammograms recorded at varying NADH concentrations from -0.675 to +1.225 V vs SHE at 10 mVs^−1^ in the presence of ubiquinone-8. **f**, Cyclic voltammogram with a clean, blank electrode recorded in 0.2 M Tris buffer, pH 7.00 in the presence of 10 mM potassium ferricyanide to calibrate the Ag/AgCl reference electrode against the known midpoint potential of potassium ferricyanide. The scan was recorded from -0.4 to +0.8 V vs Ag/AgCl at 100 mVs^−1^. All data are derived from a single representative experimental, performed after multiple rounds of optimisation. Source data

As indicated by our structural analysis, the Ncp tube interacts with the membrane through four Phe108 residues that stably embed in the bilayer and four Arg112 and Lys115 residues that coordinate the charged phospholipid headgroups (Fig. 4e,f). Over the course of the simulations, the Ndh–Ncp complex tilts so that one of the Ndh subunits contacts the membrane, with the opening of the Ncp tube penetrating the hydrophobic region of the lipid bilayer (Fig. 4a,g and Extended Data Fig. 6b). To accommodate the Ncp tube in the membrane, the phospholipid headgroups of the bilayer move away from the opening of the Ncp tube, and tails of bilayer phospholipids occupy the opening of the Ncp tube (Fig. 4g,h). This arrangement connects the hydrophobic lumen of the bilayer with the interior of the Ndh–Ncp complex, allowing for diffusion of quinone between the two structures, a process critical for long-range quinone transport. This arrangement contrasts with the recently published ForCE structure, in which phospholipid headgroups are modelled within the hydrophobic ForE tube with tails directed outward, an assignment weakly supported by the density that would occlude the proposed quinone-access pathway^19^. Our MD model offers a more chemically realistic view of DUF1641–membrane interaction.

### Quinone diffusion drives electron transfer from Ndh–Ncp to the membrane

To directly assess the ability of the Ndh–Ncp complex to utilize quinone to transfer electrons from NADH oxidation to a lipid bilayer, we performed cyclic voltammetry on the complex immobilized on an electrode-bound, MK-7-supplemented phospholipid membrane, stabilized by a mixed self-assembled monolayer consisting of EO_3_-cholesterol and 6-mercaptohexanol. In the absence of NADH, MK-7 oxidation and reduction peaks were observed in this system at +0.09 V and −0.28 V vs standard hydrogen electrode (SHE), respectively (Fig. 4i). The addition of NADH yielded a catalytic oxidative current with an onset potential of −0.075 V, close to the midpoint potential of MK-7 (ref. ^39^), indicating that MK-7 is responsible for mediating electron transfer between the electrode and the Ndh. The reductive MK-7 peak disappeared upon NADH addition, indicating that MK-7 is reduced at the Ndh active site rather than at the electrode. The electrochemical *K*_M_ for NADH, determined from the magnitude of the catalytic current at +0.3 V vs SHE at different NADH concentrations, was 91.6 µM, in agreement with our previous kinetic assays (Extended Data Fig. 6a). Control experiments lacking the enzyme showed no NADH oxidation (Extended Data Fig. 6b). These observations are consistent with the behaviour of other NDH-2 enzymes, including homodimeric *C. thermarum* NDH-2 (ref. ^40^).

We next examined the electrochemical behaviour of the complex in the absence of MK-7 in the electrode-associated bilayer. Cyclic voltammetry revealed that under these conditions, Ndh–Ncp produced a catalytic oxidative current upon the addition of 400 µM NADH, but at a reduced magnitude. We attribute this activity to the presence of MK-8 in Ndh–Ncp sequestered from *E. coli*, which decayed over repeated scans, suggesting that quinones present in the Ndh–Ncp complex are responsible for electron transfer from NADH to the electrode, and the magnitude of this transfer reduces over time as they diffuse into the lipid bilayer (Fig. 4j and Extended Data Fig. 6c). We calculated a quinone diffusion constant of 8.2 × 10^−^^4^ s^−^^1^ from the decay rate (Extended Data Fig. 6c), indicating the rate of diffusion between Ndh–Ncp and the membrane. Together with our lipidomics analysis showing quinone enrichment by Ndh–Ncp, these electrochemical data demonstrate that the complex not only sequesters quinones from the membrane but also retains them in quantities capable of sustaining Ndh catalysis away from the bilayer, providing a mechanism for extending respiratory activity beyond the membrane.

Cyclic voltammetry experiments of Ndh–Ncp immobilized on a bilayer containing UQ-8 demonstrated that Ndh is selective for MK-7, with a much greater onset potential (>0.5 V) observed for UQ-8 (Fig. 4j and Extended Data Fig. 6c,e). This is consistent with the kinetic experiments and further indicates that the MK-8 associated with the Ndh–Ncp complex is responsible for facilitating electron transfer in the electrochemical experiments in the absence of quinone supplementation in the bilayer.

### The Ndh–Ncp complex is widespread throughout Bacillota

**Extended Data Fig. 7:**
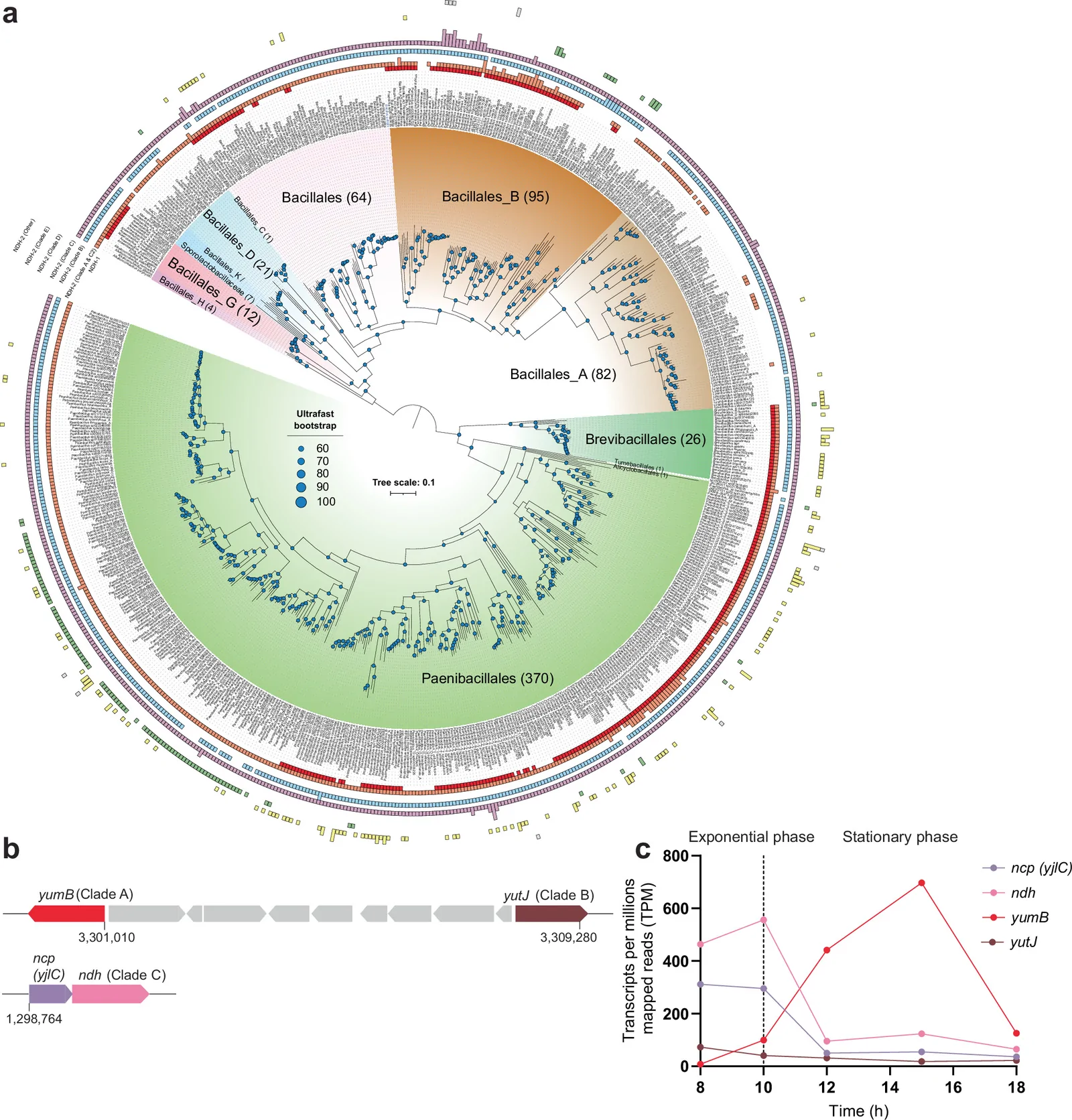
Ndh-Ncp encoding Bacillota are taxonomically widespread and co-encode multiple NDH-2 enzymes with distinct physiological functions. **a**, Mid-point rooted maximum-likelihood phylogenomic tree of 684 Ndh-Ncp encoding Bacillota species genomes from GTDB R09-RS220 using concatenated 120 GTDB bacterial marker genes and WAG + G20 model. Outer-rings show the copy numbers of Complex I (NDH-1) and various NDH-2 clades encoded by each species. Branch circles show ultrafast bootstrap support values with 1000 replicates. The scale bar corresponds to the expected number of substitutions per site. **b**, Canonical genetic arrangement of the NDH-2 from clade A (*yumB*) and NDH-2 from clade B (*yutJ*), compared to NDH-2 from clade C (*ndh*) and Ncp, using *B. subtilis* as an example. Canonical NADH-oxidizing NDH-2 (Clade A) and NADPH-oxidizing NDH-2 (Clade B) are commonly co-encoded in close genetic proximity (typically 4 – 20 genes apart) in Bacillota genomes. Clade C1 NDH-2 is always co-encoded with Ncp in the immediate genetic proximity **c**, Time-course transcriptomic profiling of NDH-2 and Ncp expression in *B. subtilis* grown in lysogeny broth, from Jun et al.^52^.

To understand the distribution and evolution of Ndh–Ncp in Gram-positive bacteria, we performed a comprehensive survey of Ndh and Ncp homologues in all 113,104 representative archaeal and bacterial species genomes from the Genome Taxonomy Database (GTDB) (Supplementary Data 4)^41^. The Ndh–Ncp complex is exclusively found in the phylum Bacillota, where 8.5% of genes encoding Ndh homologues occur directly adjacent to an *ncp* gene. The 684 Ndh–Ncp-encoding species span 30 different families, indicating that this complex is widely encoded by taxonomically diverse members of this phylum (Extended Data Fig. 7a). Genome-wide metabolic analysis suggested that the Ndh–Ncp complex occurs in aerobic or facultatively anaerobic lineages but is absent in obligately anaerobic lineages such as Clostridia and Negativicutes (Supplementary Data 4). All Ndh–Ncp-encoding species co-encode at least one additional, non-Ncp-associated NDH-2 enzyme (Extended Data Fig. 7b), suggesting distinct physiological roles of these enzymes, as exemplified by *yumB* and *yutJ* encoded by *B. subtilis*^29^.

**Fig. 5:**
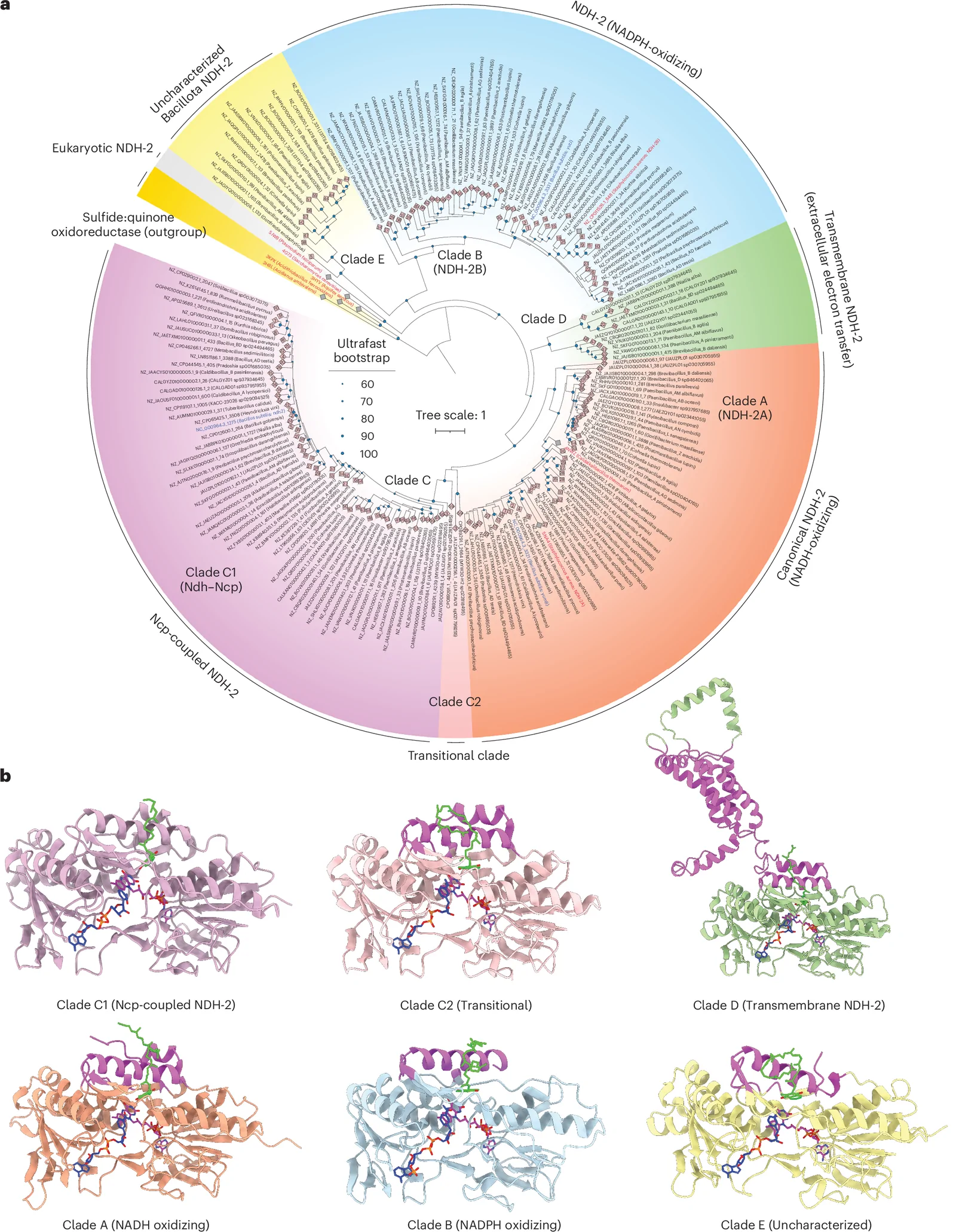
Ndh–Ncp is a recently evolved NDH-2 clade widespread among Bacillota. **a**, Maximum-likelihood phylogenetic tree of NDH-2 from Ndh–Ncp-encoding Bacillota genomes. Sequences are from a representative subset of 60 phylogenetically divergent Bacillota species (see Extended Data Fig. 7a and Supplementary Data 4 for all Ndh–Ncp-encoding species), highlighting the common presence of multiple divergent NDH-2 genes within the same organism, which probably mediate different physiological functions. The tree was constructed on the basis of 195 amino acid sequences using the LG + I + R10 model and rooted using sulfide:quinone oxidoreductases (Sqr) as an outgroup. Taxonomy of the genomes follows GTDB R09-RS220. Coloured tip labels show reference NDH-2/Sqr sequences (red) and the three *Bacillus subtilis* NDH-2 (blue). Branch circles show ultrafast bootstrap support values with 1,000 replicates. Diamond labels at the tip denote identifiers of the 60 genomes. The scale bar corresponds to the expected number of substitutions per site. **b**, Chai1-predicted structural models for representatives of each NDH-2 clade, coloured to match the tree. Membrane interaction regions are coloured in purple. Clade C1: *B. subtilis* Ndh (NC_000964.3_1275) (experimental data), Clade C2: *MYW30-H2 sp022818495* (CP089291.1_2664), Clade D: *B. daliensis* (NZ_JAJISB010000001.1_475), Clade A: *B. subtilis* YumB (NC_000964.3_3321), Clade B: *B. subtilis* YutJ (NC_000964.3_3331) and Clade E: *B. daliensis* (NZ_JAJISB010000001.1_165). Model validation is provided in Supplementary Fig. 5.

**Extended Data Fig. 8:**
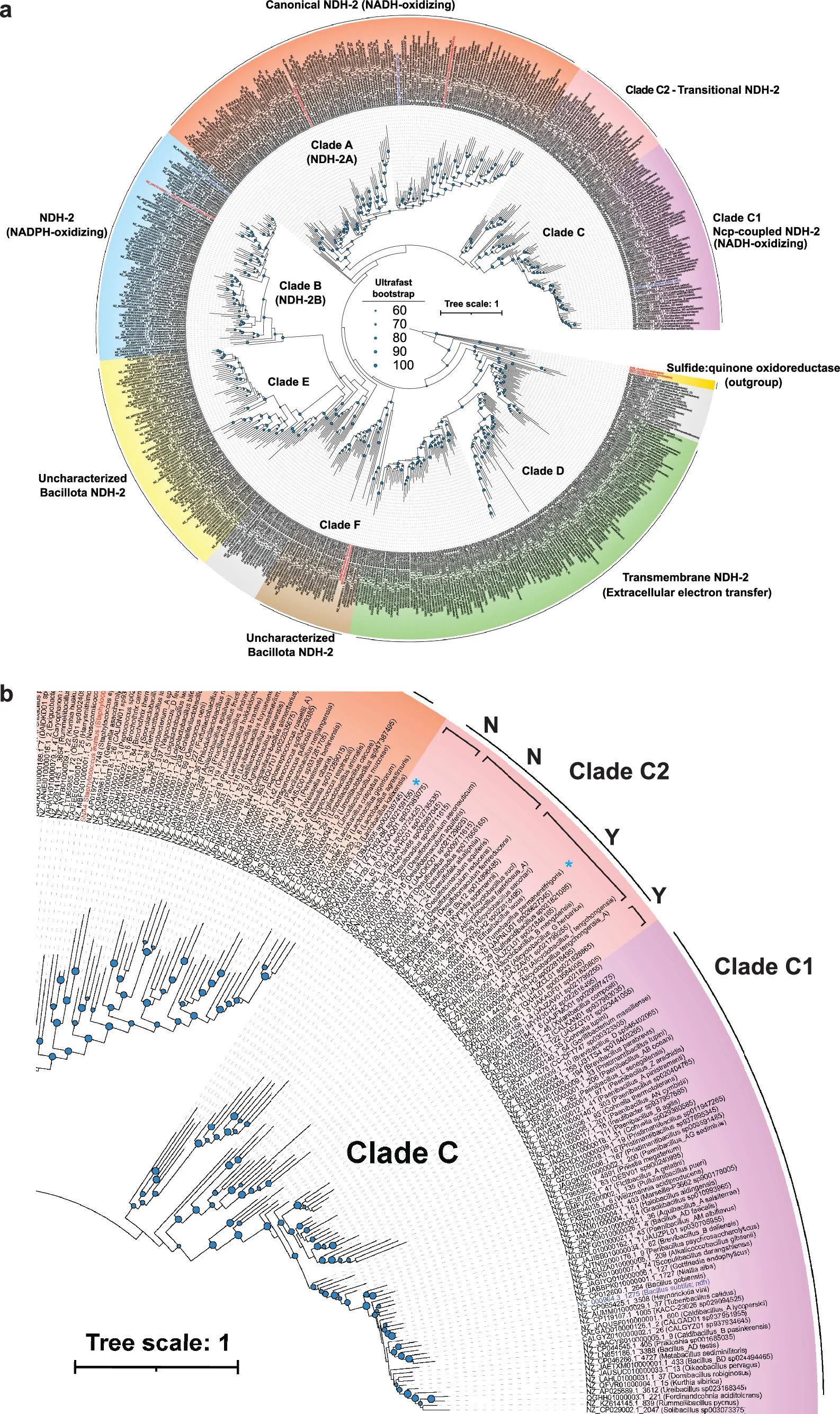
Expanded phylogenetic tree of NDH-2 across Bacillota. **a**, Maximum-likelihood tree constructed based on 683 cluster-dereplicated NDH-2 amino acid sequences from Bacillota (Ncp-coupled Ndh: 80% identity; Other NDH-2: 60% identity) using the LG + I + R10 model and rooted using sulfide:quinone oxidoreductases (Sqr) as an outgroup. Taxonomy of the genomes follows GTDB R09-RS220. Coloured tip labels show reference NDH-2/Sqr sequences (red) and the three *Bacillus subtilis* NDH-2 (blue). Branch circles show ultrafast bootstrap support values with 1000 replicates. The scale bar corresponds to the expected number of substitutions per site. **b**, Zoom of clade C NDH-2 with annotations on clade C2 showing Ndh with a DUF1641-domain protein present elsewhere in the genome (Y) or absent (N). Those annotated with a blue asterisk are outliers.

**Extended Data Fig. 9:**
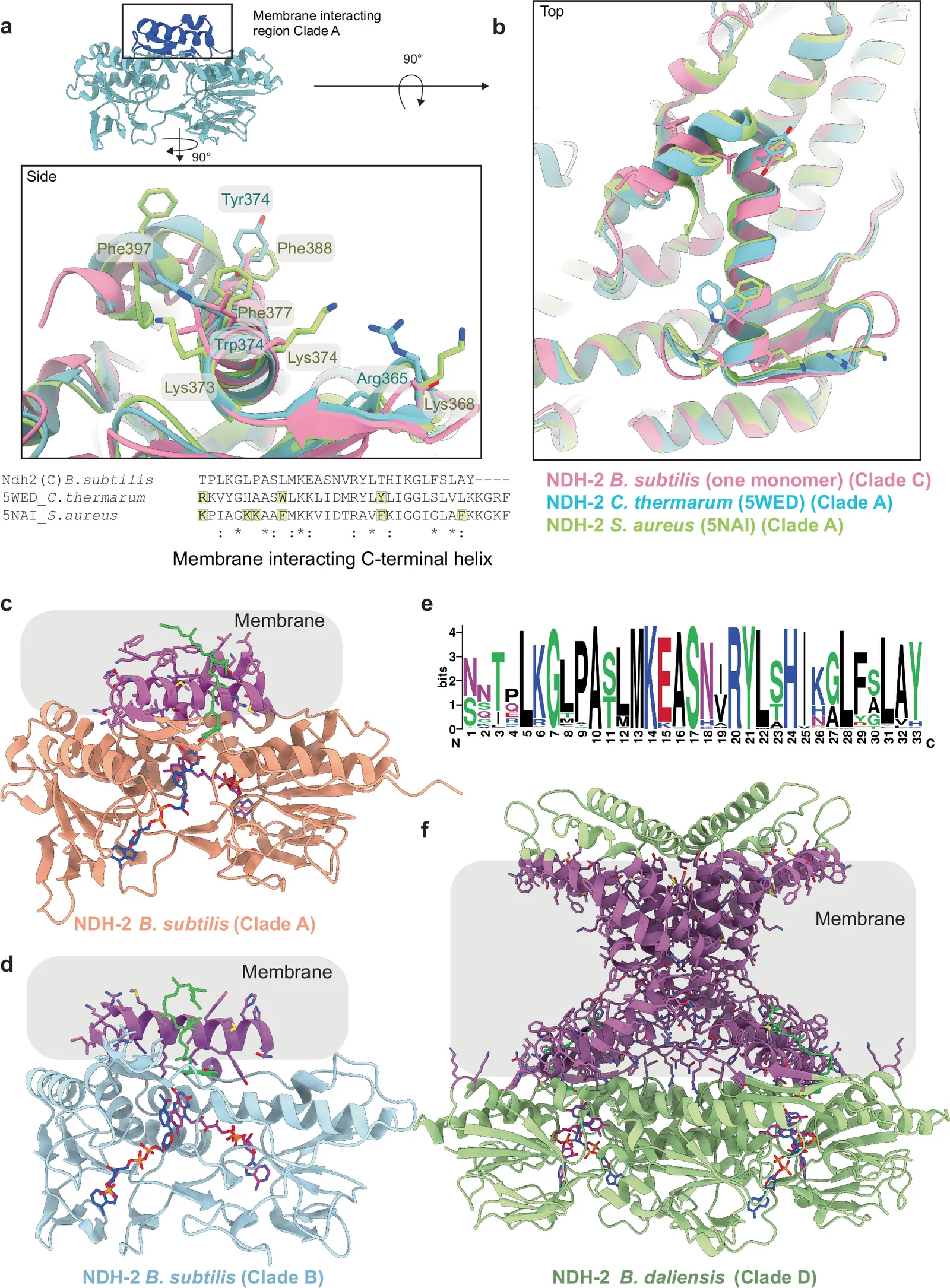
Membrane interaction modes of NDH-2 from Bacillota. **a**, Overlay of NDH-2 from *B. subtilis* (Clade C1 Ndh, this study), *C. thermarum* (Clade A, PDB: 5WED) and *S. aureus* (Clade A, PDB: 5NAI). The side view of the key residues for membrane interaction from these helices are shown. *S. aureus* (green) and *C. thermarum* (blue) have key aromatic residues that are absent in *B. subtilis* Ndh (pink). **b**, The top view of the structures shows the key residues, with the sequence alignment below demonstrating the conservation of these residues in the clade A NDH-2. **c**, Model of clade A NDH-2 (*B. subtilis*) with membrane-interacting region shown in purple **d**, Model of clade B NDH-2 (*B. subtilis*) **e**, WebLogo of the C-terminus of the Ndh from clade C1 that couple to Ncp from Bacillota **f**, Model showing the membrane-interacting region of clade D NDH-2 (*B. daliensis*). Notably, clade D NDH-2 has transmembrane helices to completely integrate into the membrane.

Phylogenetic analysis of Ndh homologues across Bacillota revealed three major monophyletic NDH-2 clades (A, B, C) and three smaller clades (D, E, F) (Fig. 5a,b, Extended Data Fig. 8a and Supplementary Data 4). All Ncp-associated NDH-2 enzymes are contained within clade C where Ncp-associated Ndh enzymes form a monophyletic group, which we refer to as Clade C1, defined by *ndh–ncp* synteny and the evolution of an Ndh C-terminal domain that mediates the stable interaction interface with Ncp (Fig. 5a, and Extended Data Figs. 8 and 9e). In addition, we identify a transitional lineage that we term clade C2. Enzymes in clade C2 retain a C terminus widely conserved in the NDH-2 family that mediates direct membrane interaction (Fig. 5a,b and Extended Data Fig. 8). Phylogenetic analysis resolves two C2 sub-branches distinguished by the presence or absence of a DUF1641-containing gene within their genome (Fig. 5a and Extended Data Fig. 8b). The DUF1641-encoding genes of this C2 branch lack the strict *ndh–ncp* synteny that defines clade C1, suggesting an intermediate stage in which the *ncp* progenitor was acquired by the ancestral bacterium, possibly forming a complex with a different enzyme, but the NDH-2 enzyme continued to interact with the membrane directly. From this transitional state, we hypothesize that the Ncp-associated Ndh enzymes in clade C1 emerged via spontaneous complex formation with the DUF1641-containing protein, followed by co-evolution to form the Ndh–Ncp. The short branch length of clade C1 relative to other clades suggests that the complex formation with Ncp is a relatively recent evolutionary innovation, but has become widely disseminated in Bacillota (Fig. 5a and Extended Data Fig. 8a).

For comparison, clades A and B represent widespread NDH-2 subtypes that frequently co-occur and genomically co-localize (separated by 4–20 genes) across divergent Bacillota species (Fig. 5a,b, Extended Data Figs. 8a and 7b, and Supplementary Data 4). Clade A represents the canonical monotopic NADH-oxidizing NDH-2, containing *yumB* of *B. subtilis* and the structurally resolved NDH-2 enzymes from *S. aureus* and *C. thermarum* (Extended Data Fig. 9a–c). These structures feature C-terminal helices that anchor NDH-2 at the membrane surface, facilitating quinone access (Extended Data Fig. 9a–c)^33,34,42^. Clade B is represented by the biochemically characterized monotopic NADPH-oxidizing NDH-2 from *S. aureus* (NDH-2B)^42^, in addition to *yutJ* of *B. subtilis* (Fig. 5a,b, and Extended Data Figs. 8a and 9d). Their genomic co-localization suggests ancestral relationships and complementary functions in NADH and NADPH oxidation. Interestingly, clade D represents NDH-2 enzymes that associate with the membrane through transmembrane helices, with previous work indicating that this clade forms a component of an extracellular electron-transport system (Extended Data Fig. 9f)^43^.

Because genomes containing *ndh–ncp* also typically encode clade A and clade B NDH-2 enzymes, we next asked why multiple NDH-2 subtypes coexist. Transcriptomic data from *B. subtilis* show that *ndh* (Ndh–Ncp) is expressed primarily during exponential growth, when respiratory activity is highest (Extended Data Fig. 7c)^44^. By contrast, *yumB* (clade A) is expressed during stationary phase and sporulation^29,44^, when energy demand is reduced, and fewer respiratory complexes occupy the membrane. *yutJ* (clade B) shows lower expression throughout, consistent with a specialized role in NADPH oxidation (Extended Data Fig. 7c)^29,44^. These complementary modes of expression and membrane interaction underscore the distinct physiological roles of Ndh–Ncp and clade A NDH-2, a division of labour that appears conserved across Bacillota.

## Discussion

**Fig. 6:**
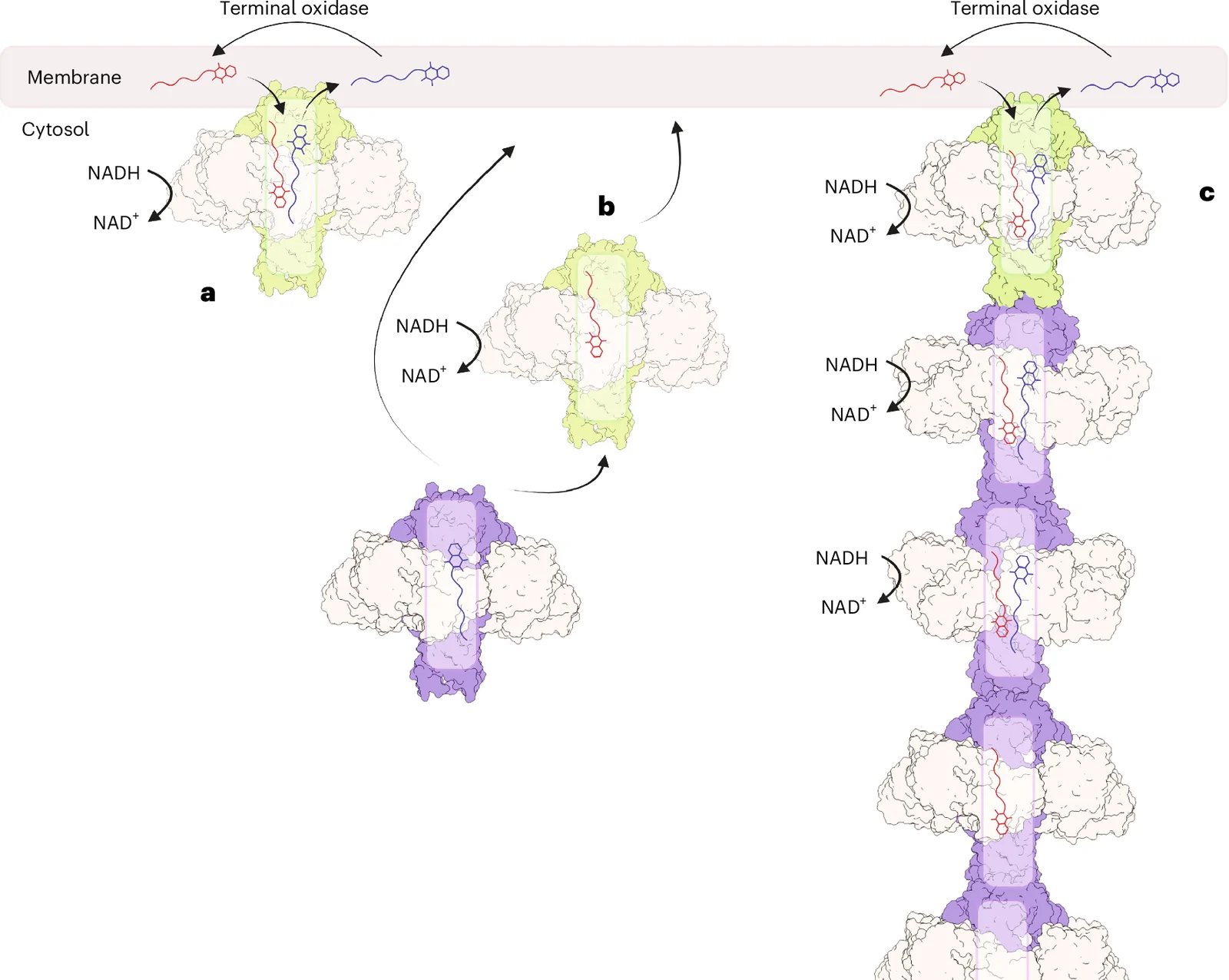
A model for quinone transfer and reduction by the Ndh–Ncp complex. **a**, Ndh can interact with the respiratory membrane through the C-terminal head region of the Ncp. The Ncp sequesters MK-7 (menaquinone in red) from the membrane for delivery to the electron acceptor sites in Ndh, where MK-7 is reduced (menaquinol in blue) by electrons from NADH oxidation. **b**, Ndh–Ncp can either remain at the membrane or dissociate using the MK-7 stored in the tube. Ndh–Ncp associates with other Ndh–Ncp complexes or directly with the membrane using the same interface. **c**, Ndh–Ncp forms a filament allowing for MK-7 to diffuse from the membrane through several Ncp tubes to the associated Ndh–Ncp chambers for storage and reduction. Created in BioRender: Kropp, A. https://biorender.com/l14erwr (2026).

Our findings show that *B. subtilis* Ndh, the primary NADH dehydrogenase in this organism, forms a stable complex with the DUF1641 protein Ncp, which enables filament formation and quinone-mediated electron transfer to the respiratory chain (Fig. 6). A defining feature of this complex is its large internal chamber, which accommodates numerous phospholipids, creating a solvent-excluded hydrophobic lumen that mimics the membrane bilayer. We observe Ndh–Ncp filaments of up to 13 units, with chambers connected into a continuous hydrophobic conduit. This arrangement could allow 52 Ndh subunits to access quinones with a membrane footprint similar to a single NDH-2 dimer embedded in the bilayer. These pseudomembrane filaments would extend NADH-driven quinone reduction into the cytosol, expanding metabolic capacity by increasing the surface area available for respiration. This structure provides an elegant solution to the constraint of limited membrane surface area^1,2^. In addition, the ability of Ndh–Ncp to sequester quinone may also enable these filaments to function as a ‘quinone battery’, concentrating quinone for reduction by Ndh, which can be exchanged during interaction with the membrane. These features distinguish Ndh–Ncp from other DUF1641-containing enzyme complexes such as Huc and ForCE, which incorporate few or no observable phospholipids and do not form filaments^19,20^.

These properties place Ndh–Ncp alongside other strategies to expand bioenergetic capacity. Thylakoids, mitochondrial cristae and the intracytoplasmic membranes of nitrifying and anammox bacteria all expand membrane area to support electron transfer^3–5,7–9^. Ndh–Ncp represents a conceptually distinct solution to this problem, a protein–lipid filament that substitutes for additional membrane surface area, by creating an internal hydrophobic tunnel for quinone transfer. This mechanism is structurally similar to electron-transferring redox enzyme filaments such as hydrogen-dependent CO_2_ reductase (HDCR), yet functionally more analogous to classical membrane elaborations^45^.

Consistent with a role in expanding respiratory capacity, Ndh–Ncp predominates during exponential growth, when respiratory demand is highest and the membrane most crowded, whereas the membrane-anchored YumB is produced under the lower respiratory load of stationary phase and sporulation^29,44^. The distribution of Ndh–Ncp across diverse Bacillota further supports the utility of this complex; although recent in evolutionary terms, it has been selected as an efficient strategy to enhance respiratory flexibility. Beyond its biological role, the Ndh–Ncp complex provides design principles for synthetic bioenergetic systems. The ability to sequester and transport hydrophobic electron carriers using soluble protein-based filaments expands possibilities for building synthetic respiratory systems with greater spatial control of electron transfer than classical bilayers.

In summary, the Ndh–Ncp complex expands the conceptual and mechanistic framework of respiratory biology. By demonstrating that respiratory enzymes can operate through protein-based pseudomembrane filaments, this work reveals a mechanism for spatially separating respiratory redox chemistry from the membrane. This strategy alleviates the challenge of limited membrane surface area while preserving efficient quinone reduction. Future work should determine the quinone capacity of these complexes, how their filaments behave in vivo and how they integrate with other respiratory systems. Together, our results establish Ndh–Ncp as a striking example of how evolution has reimagined membrane function, and provide a foundation for engineering synthetic energy-transducing systems.

## Methods

### Data analysis and visualization

In addition to programs described elsewhere in the manuscript, Microsoft Excel 2016, GraphPad Prism 10 and Origin were used for data analysis and visualization.

### Cloning and mutant construction

The *Ndh* and *ncp* genes were amplified from *B. subtilis* 168 genomic DNA (gDNA) as one continuous fragment and subcloned into a pET22b plasmid, between BamHI and NheI sites, modified to encode an N-terminal twin strep II tag, yielding the expression vector p22b–Ndh–Ncp. To generate Ndh–Ncp mutants for recombinant purification, we used the Q5 mutagenesis kit (NEB) to add 4 mutations to generate Mutant 1 (Q14A, Q16A, Q17A, D21A) and Mutant 2 (D21A, Q22A, Q29A, E30A) in the p22b–Ndh–Ncp vector. Mutants were confirmed by whole plasmid sequencing (Plasmidosaurus).

To generate isogenic deletion mutants, gDNA was isolated from the *B. subtilis* kanamycin gene deletion library strains Δ*yjlC*::kan (now Δ*ncp*::kan) and Δ*ndh*::kan as previously described^46,47^. Subsequently, our *B. subtilis* 168ca (trp+) wild-type strain was transformed with the isolated gDNA and mutants were selected on kanamycin-containing nutrient agar plates^46^. Colonies were clean streaked on fresh nutrient agar–kanamycin plates and stored at −80 °C. Deletions were confirmed by PCR and Sanger sequencing. All primers, strains and plasmids are listed in Supplementary Table 2.

### *B. subtilis* growth assay

*B. subtilis* 168ca (trp+) wild-type^48^ and isogenic mutants Δ*ncp*::kan and Δ*ndh*::kan were routinely grown overnight in lysogeny broth (LB: 10 g l^−1^ tryptone, 5 g l^−1^ yeast extract, 10 g l^−1^ sodium chloride) at 37 °C with aeration, with the addition of kanamycin at a final concentration of 5 µg ml^−1^ for the deletion mutants. The following day, cultures were normalized to a starting optical density at 600 nm (OD_600_) of 0.05 in fresh LB and transferred in 200 µl aliquots, in technical triplicate and biological duplicate, to a 96-well plate at either 37 °C or 30 °C. Growth was subsequently monitored at OD_600_ every 15 min for 12 h in a BMG Spectrostar Nano plate reader. Growth rates were calculated using OD_600_ values between 0.1 and 1.6, equating to bacterial growth during exponential phase. The rate of growth was calculated for each replicate and plotted as three individual data points. Growth yield experiments were carried out in LB + 0.5% glucose to reduce sporulation and yield calculated as OD_600_ reading at 24 h after inoculation.

### Expression of Ndh–Ncp

p22b–Ndh–Ncp and mutants were transformed into *E. coli* C41 cells and grown as a starter culture in LB media until the culture reached the stationary phase, ~12–16 h. Large cultures of 500 ml terrific broth media (TB: 12 g l^−1^ tryptone, 24 g l^−1^ yeast extract, 12.26 g l^−1^ K_2_HPO_4_, 2.31 g l^−1^ KH_2_PO_4_, 4 g l^−1^ glycerol) in a 2.5 l flask were inoculated with 5 ml of the starter culture and grown with shaking at 180 r.p.m. and 37 °C until they reached ~1.0 OD_600_. The cultures were then cooled at 4 °C for 30 min before being returned to an incubator at 20 °C and induced with 0.3 mM isopropyl β-D-1-thiogalactopyranoside (IPTG). The cultures were incubated for a further 16–18 h before collection. Cells were collected by centrifugation at 3,500 × *g* for 20 min at 4 °C using a Sorvall high-speed centrifuge. Once pelleted, cells were frozen and stored at −80 °C.

### Recombinant purification of Ndh–Ncp

Purification occurred in a two-step process, beginning with affinity purification and followed by size exclusion chromatography. Initially, cells were thawed and resuspended with lysis buffer (50 mM Tris, 150 mM NaCl, pH 8.0, 1× cOmplete EDTA-free protease inhibitor (Roche, 11836145001), 1 mg ml^−1^ DNase and lysozyme) at room temperature. Cells were then lysed by two passages through a cell disruptor (Emulsiflex C-5), and the lysate was clarified by centrifugation at 30,000 × *g* for 20 min at 4 °C. Lysate was then incubated for 5 min with BioLock biotin blocking buffer (IBA Lifesciences IBA, 2-0205-050) and loaded onto 1 ml StrepTrap XT (Cytiva). Once loaded, the column was washed with lysis buffer for ~30 column volumes (CV). The protein was eluted with elution buffer (50 mM Tris, 150 mM NaCl and 50 mM biotin, pH 8.0) in ten 1-ml fractions. Resulting fractions were analysed using SDS–PAGE, then pooled and concentrated to 500 µl for SEC using a 4-ml centrifugal concentrator with a 100-kDa molecular weight cut-off (MWCO) (Amicon, UFC803008). Concentrated protein was then loaded onto a Superose 6 Increase 10/300 GL column (Cytiva, 17517201) in SEC buffer (50 mM Tris, 150 mM NaCl, pH 8.0) and eluted in 0.5-ml fractions. Fractions were analysed by SDS–PAGE for peak identification and purity. Peaks corresponding to the correct complex were either pooled and concentrated for biochemical assays, or individual fractions were concentrated to separate each region of the peak for cryo-EM.

### Native PAGE and coupled activity stain

Native PAGE was run following manufacturer instructions (ThermoFisher Native PAGE Bis-tris). Briefly, either protein or lysate was prepared and the concentration determined, then samples were mixed with 4× loading dye to a final volume of 20 µl. Samples were loaded onto 3–12% 10-well gels, along with Native Mark molecular weight marker (ThermoFisher), and run in native running buffer (ThermoFisher) at 150 V for 90 min. Following this, gels were either stained with Coomassie (Bulldog Acqua Stain) or incubated in buffer (50 mM Tris, 150 mM NaCl, pH 8.0) containing 500 µM nitrotetrazolium blue (NBT) and 500 µM NADH. The activity-stained gels were imaged as the colour developed upon NBT reduction (to purple) indicating NDH-2 activity.

### Fractionation of Ndh–Ncp from *B. subtilis*

*B. subtilis* cells were grown overnight at 37 °C in LB media, then the following day, 1 l of LB media in 5 l flasks was inoculated to 0.01 OD_600_. Cells were grown for 6 h post-inoculation and then collected by centrifugation and frozen. Cell pellet (3 l) was thawed and resuspended in 50 mM Tris pH 8.0 until homogeneous, and 2 mg ml^−1^ of lysozyme was added to the resuspended cell solution. The cells were incubated with gentle stirring for 30 min at 37 °C. Cells were then further lysed by two passages through a cell disruptor (Emulsiflex C-5), and the lysate was clarified by centrifugation at 30,000 × *g* for 20 min at 4 °C. Lysate was applied to a QFF HP anion exchange column (Cytiva) and eluted over 10 CV with a linearly increasing gradient of 1 M NaCl. Eluted fractions were tested for activity with a plate-based activity assay (200 µl 500 µM NADH and 500 µM NBT in the plate and 2–30 µl protein added to the cell, NDH-2 activity confirmed with a purple colour change). Active fractions were pooled and subjected to SEC on a Superose 6 column. Fractions that were active were subjected to SDS–PAGE, native PAGE and negative-stain EM to visualize Ndh–Ncp.

### Mass photometry

These measurements were carried out using a Refeyn 2MP mass photometer. Protein samples and standards were diluted in phosphate buffered saline (PBS) for measurements. Initially, standards of apoferritin and BSA were measured to generate a standard curve for molecular weight calculations. Then each protein sample was measured at different concentrations (10–100 nM) to determine the optimal concentration for accurate reads. At the sample-dependent optimal concentration, each sample was measured three times and one representative from each sample was used in the figure. Data were analysed using Refeyn DiscoverMP software.

### Visible-wavelength spectroscopic measurements

These measurements used a SpectraMax ABS Plus (Molecular Devices) 96-well plate reader fully housed in an anaerobic glovebox (O_2_ < 3 ppm; Belle Technology). A concentration of 85 µM of enzyme was carefully deoxygenated, diluted with anaerobic buffer (50 mM Tris-Cl pH 8, 150 mM NaCl), and spectra were recorded in a quartz cuvette following the addition of NADH (0–100 µM). Data were plotted using GraphPad Prism 10.6.

### Steady-state oxidation of NADH measurements

The oxidation of NADH was measured at 340–380 nm (*ε* = 4.81 mM^−1^ cm^−1^) in a SpectraMax ABS Plus (Molecular Devices) 96-well plate reader. The buffer (50 mM Tris-Cl pH 8, 150 mM NaCl) contained 0.15% asolectin-CHAPS to provide a hydrophobic phase for the short-chain quinones. Menadione reaction kinetics were measured with the addition 4 nM Ndh–Ncp at a concentration range of 0–120 µM menadione or 0–500 µM NADH. Decylubiquinone was measured with the addition of 8 nM Ndh–Ncp over a concentration range of 0–120 µM decylubiquinone or 0–500 µM NADH. The data were fit to the Michaelis–Menten equation (*v* = *V*_max_ × [*S*]/(*K*_M_ + [*S*])), with [*S*] corresponding to substrate concentration, using Solver in Microsoft Excel. Data were plotted in GraphPad Prism 10.6 for figures.

### Negative-stain and cryo-EM imaging and data collection of Ndh–Ncp

Negative-stain grids (carbon film 300 mesh, EMS) were prepared using first glow discharge for 30 s at 15 mA, and then the protein was applied (0.01 mg ml^−1^) for 1 min, followed by blotting of the excess protein. Three rounds of uranyl acetate (1–2%) application followed (two at 30 s, the final for 1 min, each followed by blotting). Grids were then imaged in a Talos L120C transmission electron microscope (ThermoFisher) at ×72,000 magnification.

Cryo-EM grids were prepared using a Vitrobot Mark IV vitrification device (ThermoFisher) pre-cooled to 4 °C and equilibrated to 100% humidity. UltrAuFoil 1.2/1.3 300 mesh grids (Quantifoil) were glow discharged for 180 s at 15 mA. Protein (4 µl) was applied at 6 mg ml^−1^ concentration to the grid after glow discharge. For the NADH-bound structure, Ndh–Ncp (at 6 mg ml^−1^) was incubated with 5× molar concentration of NADH (~130 µM) 10 min before freezing. The grid was then plunge frozen into liquid ethane using the Vitrobot. Grids were immediately clipped for the autoloader and stored long term in liquid nitrogen.

Samples were initially screened on a Talos Arctica 200 kV transmission electron microscope, collecting a small dataset (200–300 movies) to generate two-dimensional (2D) class averages with cryoSPARC live^49^. Large datasets were collected on a Titan Krios G4 transmission electron microscope fitted with a Gatan K3 direct electron detector with energy filter (Ndh–Ncp) or a Falcon4i direct electron detector (Ndh–Ncp NADH/quinone bound). Data were collected at ×130,000 magnification (0.655 Å pixel^−1^) with a total dose of 39 e^−^ Å^−2^, fractionated into 40 frames for Ndh–Ncp, and ×96,000 magnification (0.808 Å pixel^−1^) with an exposure time of 3 s and a total dose of 50 e^−^ Å^−2^, fractionated into 50 frames for Ndh–Ncp NADH/quinone bound. A C2 aperture of 42 µm was used with the objective aperture inserted and a defocus range between −1.2 µm and −0.6 µm. Data collections were automated using the EPU v.3.6 software (ThermoFisher).

### Cryo-EM data processing and analysis

#### Ndh–Ncp

Micrographs (7,876) were grouped into their respective optics groups using the Python script AFIS.py, and the grouped micrographs were then motion corrected using UCSF MotionCor3 and contrast transfer function (CTF) estimation (CTFFIND-4.1) in Relion (5.0)^50^. The particle coordinates were then determined with crYOLO (1.7.6) using a general model and extracted in Relion with a box size of 512 px (refs. ^50,51^). Initial 2D classification was carried out in Relion 5.0, then extracted and imported into cryoSPARC v.4.6.0 for ab initio reconstruction, with one class with C4 symmetry^49^. Homogeneous refinement of 483,556 particles was followed by non-uniform refinement with C4 symmetry. 3D classification followed with 6 classes. Four classes were selected containing 389,955 particles and subjected to another round of non-uniform refinement with C4 symmetry. This generated a map of 2.14 Å global resolution which was improved to 2.12 Å resolution with local refinement, masking only one unit of the filament. From this, helical refinement was carried out with a helical rise of 99.22 Å, twist of 23.95° and final resolution of 3.09 Å. Alternatively, the particles from the local refinement were reclassified with heterogeneous refinement, masking one class with ‘bad particles’. The resulting ‘good particles’ were reclassified in 2D space, and 356,549 particles were selected for local refinement, resulting in maps reaching a global resolution of 2.11 Å (Supplementary Fig. 2).

#### Ndh–Ncp NADH/quinone bound

Micrographs (11,418) were grouped into their respective optics groups using the Python script AFIS.py, and the grouped micrographs were then motion corrected using UCSF MotionCor3 in Relion 5.0. The motion-corrected particles.star files were transferred to cryoSPARC v.4.6.0 for CTF estimation^49,50^. Particle coordinates were determined initially using Blob Picker to generate 2D averages to train Topaz Picking^52^. Topaz Picking was trained only on the 2D class of the top view, in an effort to find more of this underrepresented view. Topaz Picking resulted in 5,095,952 particles, which were extracted at a 420-pixel box size, binned to 120 px, and 2D classification resulted in 2,590,595 particles selected. Ab initio reconstruction was carried out with two classes and C4 symmetry, with the largest class containing 1,301,383 particles used for homogeneous refinement (C4). Homogeneous refinement was followed by heterogeneous refinement with two classes, one masked with the ‘junk’ ab initio class, the other masked from homogeneous refinement. A total of 797,409 particles were used for non-uniform refinement to generate a new model for Topaz Picking. A total of 1,894,345 particles resulted and were extracted at a 420-pixel box size, binned to 120 px. A total of 1,267,220 particles were selected from 2D classification and re-extracted without binning. 2D classification further resulted in 1,217,300 particles selected and ab initio reconstruction was carried out with two classes and C4 symmetry. Homogeneous refinement (C4) with 632,082 particles resulted in maps to a global resolution of 2.58 Å. Further non-uniform refinement and 3D classification using heterogeneous refinement (two rounds) resulted in a non-uniform refinement with 341,297 particles to a global resolution of 2.52 Å (Supplementary Fig. 3).

Of note, processing of both datasets was also carried out using C1 symmetry for 3D refinement; however, maps from C1 processing were of lower resolution, displayed anisotropy due to orientation bias, and did not reveal additional structural features. As such, subsequent processing focused on C4 symmetry.

### Model building and visualization

An initial model of the Ndh–Ncp complex was generated with AlphaFold3 (ref. ^53^), and Ncp and Ndh were then separated into individual molecules using Pymol (3.1.8)^54^. The Ncp tetramer and Ndh enzymes were docked separately into the maps generated, using ChimeraX 1.8 fit map^55^. The models were built and refined using Coot 0.9.8.1 and Phenix (1.2)^56,57^. FAD, MK-8 and NADH were modelled into the complexes using Coot^57^. Model quality was validated using MolProbity^58^. All figures were made using ChimeraX (1.8)^55^.

### Mass spectrometric lipid detection and analysis

Ndh–Ncp purified in *E. coli* was incubated with *B. subtilis* 168 membranes extracted from 4 l of *B. subtilis* cultures or left untreated. To prepare the membranes, *B. subtilis* was grown in LB at 30 °C for 24 h, then collected by centrifugation at 3,500 × *g* and frozen at −20 °C. Cells were lysed in buffer (50 mM Tris, 150 mM NaCl, 0.5 mM EDTA, pH 7.5) and incubated with 1 mg ml^−1^ lysozyme at 37 °C for 30 min. Cells were then incubated with 1× cOmplete EDTA-free protease inhibitor (Roche, 11836145001) and 1 mg ml^−1^ DNase for 10 min at room temperature, followed by lysis with two passages through a cell disrupter (Emulsiflex C-5). The lysate was clarified by centrifugation at 27,000 × *g* for 20 min at 4 °C, and the membranes were then collected by ultracentrifugation at 100,000 × *g* for 1 h at 4 °C. Membranes were resuspended in 50 mM Tris, 150 mM NaCl, pH 7.5 and incubated for 6 h at 4 °C with 5 mg of Ndh–Ncp, rotating slowly. The membranes were then pelleted by ultracentrifugation, and the resulting protein was purified initially by affinity chromatography with a StrepXT column (Cytiva), then the flow through of unbound samples was purified by SEC on a Superose 6 Increase 10/300 GL column (Cytiva, 17517201) in SEC buffer (50 mM Tris, 150 mM NaCl, pH 8.0). Resulting eluted fractions were pooled, concentrated and frozen for mass spectrometry. Samples from each fraction were analysed by negative-stain TEM before concentration.

Samples corresponding to 48.6 µg of purified protein (determined using absorbance at 280 nM) were prepared for liquid chromatography–mass spectrometry (LC–MS) analysis using a modified Folch extraction. Briefly, a 100 μl solution of purified protein (~48.6 μg) or 1 µl of isolated membrane diluted to 100 µl with LC–MS-grade water was treated with 2,000 μl of 2:1 chloroform:methanol v/v containing 50 ng ml^−1^ of menaquinone-7 (d7) (90%, LGC Standards, TRC-KIT6138) and EquiSPLASH (Avanti, 330731-1EA) lipid mixture with each standard at 50 ng ml^−1^, after which the mixture was shaken for 10 min and allowed to stand for a further 50 min. Water (400 μl) was added, and the mixture was shaken for 10 min, after which the sample was allowed to stand until the two phases had completely separated. The lower chloroform-rich phase was then transferred to a 1.4 ml total recovery glass microvial (ThermoFisher, 6PSV9-CHV1) and the solvent was removed under a stream of nitrogen gas. Samples were then reconstituted in 60 µl of 7:3 LC Solvent A:LC Solvent B (described below). Samples were analysed using a Vanquish Horizon ultra-high-performance liquid chromatography platform (ThermoFisher) coupled to an Orbitrap Exploris 480 MS (ThermoFisher) using a C18 column (Zorbax Eclipse Plus C18 Rapid Resolution HD, 2.1 × 100 mm, 1.8 μm, Agilent, 959758-902) with a binary solvent system: solvent A = 40% isopropanol/60% water and solvent B = 98% isopropanol/2% water, both containing 2 mM formic acid and 8 mM ammonium formate. Linear gradient time–%B were as follows: 0 min–0%, 8 min–35%, 16 min–50%, 19 min–80%, 23 min–100%, 28 min–100%, 30 min–0%, 32 min–0%. The flow rate was 250 μl min^−1^, the column temperature 50 °C, and the sample injection volume was 10 μl. Each sample was analysed three times, first at an MS1 level using fast polarity switching, then by data-dependent acquisition (DDA) in a single polarity, that is, one analysis in positive mode and one analysis in negative mode. For the polarity switching analysis, the mass spectrometer operated at a resolution of 120,000 from *m*/*z* = 140–2,000 and DDA was performed with an MS1 survey scan *m*/*z* = 450–2,000 at a resolution of 60,000. The resulting data-dependent MS/MS was conducted at a resolution of 15,000 using an isolation window of 1.5 *m*/*z* and a stepped normalized collision energy of 20, 40 and 60. The method cycled with a loop count of 7 and dynamic exclusion of 8.0 s. The following source conditions were used in all experiments: spray voltage 3.5 kV (positive), 2.5 kV (negative); capillary temperature 325 °C; sheath gas 60; aux gas 15; sweep gas 2 and probe temp 320 °C.

Lipidomic data analysis was performed using untargeted feature extraction on the MS1 polarity switching data in mzMine (4.3)^59^. Briefly, this involved untargeted feature extraction from each sample, alignment of features, gap filling and identification against those features previously identified. Features that did not exceed the signal found in the extraction blanks by at least 5-fold were excluded. Lipids were then putatively identified by comparison against the lipid identification, which was achieved by comparing the MS/MS spectra against the LipidBlast database^60^ and/or the MSDIAL-TandemMassSpectralAtlas-VS69 (ref. ^61^), followed by manual review of the putative annotations. Unassigned features were then annotated on the basis of their accurate mass against the Lipid Maps structural database^62,63^ (MS1ID) and/or the MycoMass database^64^. Raw and processed data can be found at https://zenodo.org/records/21271664. In addition, the molar amount of PE, PG, MK, DMK and CoQ was determined by comparison of each lipids’ peak area against that of the isotopically labelled standard of the same class for PE and PG, or against the menaquinone-7 (d7) standard for MK, DMK and CoQ lipids. Peak integration was performed using TraceFinder 5.2 SP3 (ThermoFisher).

To assess enrichment of MK-7 and related quinone species in the Ndh–Ncp complex following incubation with *B. subtilis* membranes, we performed a semi-quantitative analysis based on within-sample lipid intensity ratios. This analysis was designed to provide a qualitative indication of quinone enrichment across compositionally distinct samples rather than absolute quantification. Lipidomic features (*n* = 10,387; unprocessed output) were filtered to retain a high-abundance, reproducibly detected subset suitable for ratio-based comparisons. Features lacking a putative annotation and internal standards were removed. Remaining features were ranked by summed intensity across all samples, and features with a summed intensity below 1 × 10^8^ across all conditions were excluded, retaining approximately the top 6% of detected features for downstream analysis. To ensure reliable detection, features were required to exceed 3-fold the maximum blank signal in at least two replicates within at least one condition. Features were retained if they met a coefficient of variation threshold of ≤30% in at least one sample group, reflecting the substantial compositional differences between sample types (*E. coli* membranes, *B. subtilis* membranes and purified complexes). Filtered features were consolidated at the lipid species level by summing intensities across adducts, yielding 278 unique lipid species. To define a *B. subtilis*-specific reference lipid set, species were selected that were present in *B. subtilis* membranes but absent or present at <10% relative intensity in the Ndh–Ncp complex before membrane incubation, yielding 113 species (excluding MK-7 itself, which served as the ratio numerator).

Because class-matched internal standards were not available for all lipid classes in the reference pool, intensity ratios were calculated from unnormalized peak intensities to avoid asymmetric normalization between numerator and denominator. The deuterated MK-7 internal standard was used to monitor quinone signal stability across samples; however, as no equivalent deuterated standard was available for the denominator lipid classes, it was not applied to ratio calculations. Quinone enrichment was quantified as the ratio of endogenous MK-7 intensity, or the summed intensity of all detected quinone species, to the summed intensity of the *B. subtilis*-specific reference lipid set within each sample. To assess robustness to denominator selection and mitigate potential biases arising from class-specific ionization or chromatographic effects, analogous ratios were calculated using independent reference sets, including a subset putative of dihexosyldiacylglycerol (Hex_2_DG) species (*n* = 5) and a single abundant putative ether-linked diacylglycerol species (DG O-32:2; DG P-14:0/18:1). Consistent enrichment trends across these independent analyses indicate that the observed quinone enrichment is not driven by the behaviour of a specific lipid class or feature subset^65–67^.

### Molecular dynamics simulations

We performed all-atom classical molecular dynamics (MD) simulations on the high-resolution cryo-EM structures of *B. subtilis* Ndh–Ncp complex with and without MK-7/NADH. Simulations were performed with GROMACS (2022.3/4)^68^ using the CHARMM36m force field for proteins and CHARMM36 for lipids, ions, water and redox-active components (FAD, MK-7, NADH)^69–72^. The membrane bilayer was constructed with the CHARMM-GUI^73^ tool, and comprised 950 POPE (~79%) and 259 POPG (~21%) lipids. The total system size was ~664,000–666,000 atoms depending on lipid and substrate arrangements (see more below). In total, six simulation setups were constructed (Supplementary Table 3).

Two strategies were applied for lipid placement in the Ndh–Ncp complex. In the first (setup 1), lipids were manually embedded into Ndh–Ncp interface cavities with VMD’s graphical interface^74^. The lipid head groups were oriented towards the solvent and tails directed inward, resulting in 33 POPE and 7 POPG molecules in a tightly packed chamber. In the second approach (setup 2), a pre-equilibrated bilayer patch was aligned using the ‘psfgen’ plugin^74^ with Ncp, and only lipids inside the chamber were retained, resulting in a total of 20 POPE and 7 POPG molecules (setups 2, 3, 4, 6, Supplementary Table 3). Lipids near the N-terminal region were added similarly.

All membrane-protein model systems were solvated with TIP3P water and 150 mM Na^+^/Cl^−^, using VMD’s ‘autoionize’ and ‘solvate’ plugins^74–76^. Protonation states were predicted using PROPKA at pH 7.0 (ref. ^77^). In all Ndh monomers, His383 was modelled protonated in non-substrate-bound setups, whereas Glu168 was protonated only in the MK-7/NADH-bound setups. Simulations were performed in the isothermal-isobaric (NPT) ensemble (310 K, 1 atm) with semi-isotropic pressure coupling. MD equilibration used the V-rescale thermostat and C-rescale barostat, followed by production simulations with Nosé–Hoover thermostat and Parrinello–Rahman barostat^78–82^. A 2-fs timestep was used with the leapfrog integrator, and all covalent bonds involving hydrogen atoms were constrained using the LINCS algorithm^83,84^. Non-bonded interactions were applied with a 12 Å cut-off and particle-mesh Ewald (PME)^85,86^ for long-range electrostatics.

The membrane patch was pre-equilibrated for 10 ns by applying harmonic position restraints (20,000 kJ mol^−1^ nm^−2^) to the phosphate groups of all lipids, followed by a 30-ns production run to generate a fully equilibrated lipid bilayer. Simulations of membrane-protein systems (Supplementary Table 3) began with a 1,000-step energy minimization using NAMD (2.14)^87,88^, with harmonic position restraints (force constant of ~41.4 × 10^3^ kJ mol^−1^ nm^−2^) applied to all heavy atoms of the protein and its substrates. This was followed by a steepest-descent minimization in GROMACS 2022.3 with an energy tolerance of 250 kJ mol^−1^ nm^−1^, using restraints (20,000 kJ mol^−1^ nm^−2^) on the same atoms. Subsequently, equilibrations were performed in the NPT ensemble with identical position restraints for 100 ns to relax the lipids within the Ncp tube, followed by a 100-ns equilibration by keeping the same harmonic restraints now only on the protein backbone and FAD atoms. Three 1-µs production simulation replicates were run per setup, where replicates 2 and 3 were generated by extending the last equilibration step by 1 and 2 ns, respectively. In addition, in substrate-bound systems, weaker restraints (2,000 kJ mol^−1^ nm^−2^) were applied to NADH and MK-7 headgroups during equilibration setups. It is noteworthy that MK-7 molecules (setup 5), after binding to FAD for ~300 ns, diffused from their structural locations. Therefore, in additional simulations (setup 6, Supplementary Table 3), harmonic distance restraints were applied between FAD (N5) and NADH (C5N), and between FAD (N3) and the MK-7 (OR8) atoms, using the GROMACS pull code with a force constant of 5,000 kJ mol^−1^ nm^−2^ (ref. ^68^). Volume calculations of solvent accessible surface (SAS) were performed with the MSMS program^89^ combined with the Openstructure software package^90^.

### Synthesis and characterisation of *S*-cholesterol (for electrochemistry membrane bilayer)

#### Synthesis of ethyl-trioxyethanol-cholesteryl 2

2-[2-(2-Aminoethoxy)ethoxy]ethanol **1** (1.08 g, 1.060 ml, 7.22 mmol) was dissolved in anhydrous dichloromethane (DCM) (25 ml) and triethylamine (0.82 g, 1.12 ml, 8.04 mmol) was then added. Separately, cholesteryl chloroformate (3.62 g, 8.04 mmol) was dissolved in anhydrous DCM (50 ml). The solution of cholesteryl chloroformate was then added dropwise to the solution of 2-[2-(2-aminoethoxy)ethoxy]ethanol, and the resultant solution was stirred for 48 h at room temperature under N_2_. After this time, the reaction mixture was diluted with additional DCM (100 ml) and washed with 1 M HCl (2 ×40 ml), then brine (40 ml). The organic layer was dried over MgSO_4_ and concentrated in vacuo to yield a crude residue, which was purified via silica column chromatography (hexane → ethyl acetate) to yield **2** as a colourless oily foam (3.95 g, 97%). Note, when performing TLC, that the location of **2** can be visualized with permanganate stain (Supplementary Fig. 6a).

#### Preparation of ethyl-trioxythioacetate-cholesteryl 3

**2** (1.95 g, 3.47 mmol) was dissolved in anhydrous DCM (50 ml). Triethylamine (630 μl, 4.51 mmol) was then added, followed by *p*-toluenesulfonyl chloride (0.860 g, 4.51 mmol). The reaction solution was then stirred at room temperature under N_2_ overnight. After this time, the reaction solution was diluted with additional DCM (50 ml) and washed with 1 M HCl (2 × 20 ml), saturated NaHCO_3_ (20 ml) and then brine (20 ml). The organic layer was dried over MgSO_4_ and concentrated in vacuo to yield the crude tosylate intermediate. The resulting residue was then redissolved in anhydrous dimethylformamide (DMF) (20 ml) and potassium thioacetate (1.19 g, 10.4 mmol) was then added. The resultant solution was then placed under a nitrogen atmosphere and stirred at 90 °C for 3 h, during which time a strong brown colour developed. The reaction solution was then cooled to room temperature and the majority of the DMF was removed in vacuo. DCM was then added (100 ml) and the resultant solution was transferred to a separating funnel. The solution was washed with water (2 ×50 ml) and then brine (20 ml), and subsequently dried over MgSO_4_. The product, **3**, was then isolated via silica column chromatography (10% ethyl acetate (EtOAc) in petroleum ether), yielding a deep-brown oil (0.910 g, 42%) (Supplementary Fig. 6b).

#### Synthesis of ethyl-trioxythiol-cholesteryl 4

**3** (0.882 g, 1.42 mmol) was dissolved in ethanol (10 ml). Separately, potassium hydroxide (169 mg, 3.01 mmol) was dissolved in ethanol (10 ml) and delivered to the solution of **3**. The reaction mixture was placed under a nitrogen atmosphere and stirred at room temperature for 3 h before being diluted in DCM (50 ml). The reaction mixture was then transferred to a separating funnel. The solution was washed with 1 M HCl (10 ml), followed by brine (20 ml), and then dried over MgSO_4_. Concentration in vacuo yielded a crude brown residue, which was further purified via silica column chromatography (hexane → ethyl acetate). **4** was isolated in resolved fractions as both its free-thiol monomeric form (0.419 g, 51%) and its corresponding disulfide dimer (0.209 g, 26%) in a combined yield of 77% (both the free-thiol and disulfide forms of the product can be used identically for self-assembled monolayer (SAM) formation). Both forms of **4** were brown in colour (Supplementary Fig. 6c).

### Electrode modification

Liposomes were formed from a solution of 5 mg ml^−1^ soy phospholipids (Sigma Aldrich) in aqueous buffer (20 mM MOPS, 30 mM Na_2_SO_4_, pH 7.40). This suspension was extruded through an Avanti mini-extruder containing a Cytiva 0.1 μm membrane filter 31× to generate liposomes. When quinone supplementation was required, 0.44 mg ml^−1^ menaquinone-7 (‘MK-7’, Cambridge Bioscience) or 0.44 mg ml^−1^ ubiquinone-8 (‘UQ-8’, Merck) was added to the lipid suspension before extrusion.

BASi gold disc electrodes (3 mm diameter) were first mechanically polished in air using alumina slurries of decreasing particle size (1, 0.3, then 0.05 μm, Metprep) applied to polishing pads (Beuhler), with 2-min polishes used per alumina size followed by sonication for 3 min in water, then ethanol. The electrodes were then taken into a glovebox (in-house construction, Belle Technology purification columns, [O_2_] < 30 ppm) and an electrochemical polish of 50 cyclic voltammograms from −1.3 to 0.7 V vs Ag/AgCl at 100 mV s^−1^ in 0.5 M NaOH were followed by 50 cyclic voltammograms from −0.25 to 1.55 V vs Ag/AgCl at 100 mV s^−1^ in 0.5 M H_2_SO_4_. Remaining in the glovebox, the cleaned electrodes were left to dry and then immersed in 1 ml of 2-propanol solution containing 8.9 mM 6-mercaptohexanol and 1.1 mM EO_3_-cholesterol (*S*-cholesterol) for 16 h to form a mixed SAM, following previously published procedure^40^. Following this SAM formation, the electrodes were removed from the glovebox and rinsed with ethanol and dichloromethane in the laboratory atmosphere and dried under N_2_. The electrodes were then returned to the glovebox, and to form the liposome functionalization, 5 μl liposome was pipetted onto the tip of the electrode, and 1 μl 120 mM CaCl_2_ in the same buffer solution was added with gentle mixing using the pipette tip. This was left to dry (~30 min), then the same liposome and CaCl_2_ dropcast plus drying process was repeated. Enzyme modification of the electrode was then achieved by applying 5 μl 7 mg ml^−1^ *Bacillus subtilis* Ndh–Ncp in 50 mM Tris, 150 mM NaCl, pH 7.4 to the electrode, and leaving this to dry for ~30 min.

### Electrochemical set up

All experiments were carried out in a 3-electrode cell containing 10 ml buffer solution (20 mM MOPS, 30 mM Na_2_SO_4_, pH 7.40), which was stirred continuously throughout. The cell used a modified gold disc working electrode (see above), a platinum wire counter electrode and an Ag/AgCl reference electrode, which was determined to be offset from SHE by +0.225 V based on calibration experiments using ferricyanide (Extended Data Fig. 6f)^91^. All experiments were carried out in a glovebox under a nitrogen atmosphere ([O_2_] < 30 ppm) at 19 °C. During a NADH titration experiment, the potential was continuously cycled at 10 mV s^−1^, with aliquots of a concentrated stock solution of 100 mM NADH added in increments at approximately −0.4 V vs SHE during every even scan. Every odd scan is displayed.

### Genomic survey, synteny characterization and phylogenetic analysis

To profile the distribution and diversity of the Ndh–Ncp complex across the prokaryotic tree of life, we retrieved all 113,104 representative archaeal and bacterial species genomes from the Genome Taxonomy Database (GTDB) release 09-RS220 (ref. ^41^). Genes were predicted using Prodigal (v.2.6.3)^92^ and functionally annotated against KEGG^93^ (accessed 22 November 2021) and Pfam (v.34.0)^94^ protein databases through DRAM (v.1.2.4)^95^. DUF1641-domain family proteins (including Ncp) were identified by searching against the Conserved Domain Database (CDD)^96^ profiles pfam07849 (DUF1641-domain) and COG2427 (YjgD/ForE, DUF1641-domain protein) using rpsblast (-*e*value 1 × 10^−5^ -max_hsps 1 -max_target_seqs 5) in BLAST+ (v.2.16.0)^97^, as well as against all entries of DUF1641-domain proteins from UniProt^98^ using DIAMOND (v.2.1.10)^99^. Hits were cross-compared and manually inspected to filter false positives. For further curation and the analysis of genetic context, five genes upstream and downstream of DUF1641-domain hits and their annotations by DRAM were retrieved. Type II NADH dehydrogenases (NDH-2) in the neighbourhood and across genomes were identified by matches to KEGG Orthology KO K03885. In total, 43,976 and 5,479 genomes were found to encode NDH-2 and DUF1641-domain proteins, respectively; 684 genomes from exclusively the phylum Bacillota encode the Ndh–Ncp complex wherein *ndh* and *ncp* are always genomically co-located next to each other (Supplementary Data 4).

Genomes and NDH-2 sequences of Bacillota were further analysed to investigate the potential physiological relevance and evolution of the Ndh–Ncp complex. The presence of major aerobic and anaerobic respiratory enzymes (including complexes I–V and terminal oxidases) in Bacillota genomes was identified on the basis of DRAM and KEGG annotation. A phylogenomic tree of the Ndh–Ncp encoding Bacillota was constructed by aligning the concatenated 120 GTDB bacterial marker genes using GTDB-Tk (v.2.4.0)^100^, followed by a maximum-likelihood phylogenetic reconstruction using IQ-TREE (v.2.4.0)^101^ (model: WAG + G20; 1,000 ultrafast bootstrap replicates; mid-point rooted). For phylogenetic analysis of Bacillota NDH-2, Ncp-associated and non-Ncp-associated sequences with lengths over 300 amino acids were respectively clustered at identity thresholds of 80% and 60% using MMseqs2 (ref. ^102^) (-c 0.7–cov-mode 1 --cluster-mode 2 -s 7.5). A multiple sequence alignment of NDH-2 cluster representatives, structurally resolved NDH-2 (PDB 5NA4, 4NWZ, 4G73, 5JWB) and sulfide:quinone oxidoreductases (PDB 3H8L, 3HYV, 3KPK) was built using the align function of Muscle (v.5.3)^103^. Using ModelFinder^103,104^ implemented in IQ-TREE v.2.4.0, the best-fit model according to Bayesian information criterion (BIC) was determined to be LG + I + R10. A maximum-likelihood phylogenetic tree was then constructed using IQ-TREE v.2.4.0 (-m LG + I + R10 -B 1000 -bnni) and rooted using sulfide:quinone oxidoreductases as outgroups (Extended Data Fig. 9). To visualize the common presence of multiple copies of NDH-2 of different clades within the same microorganism, a maximum-likelihood phylogenetic tree using all NDH-2 sequences from a subset of 60 representative and phylogenetically divergent Ndh–Ncp encoding Bacillota species was also constructed with the same method (Fig. 5 and Supplementary Data 4). All phylogenetic trees were visualized using iTOL (v.7)^105^. Major clades of NDH-2 were defined in this study on the basis of phylogeny, structural features (for example, transmembrane domain, interaction with Ncp) and reported physiological roles (for example, NADPH/NADH preference, extracellular electron transfer). NDH-2 and Ncp sequences, synteny of Ndh–Ncp, NDH-2 classification, genome statistics and metabolic annotations of the Bacillota genomes are summarized in Supplementary Data 4.

### Structural modelling

Modelling was performed on the Chai1 or AlphaFold3 online server^53,106^. Entire sequences were provided along with NADH (or NADPH)/FAD/MK-7/MK-8/UQ-8 smiles string.

### Reporting summary

Further information on research design is available in the Nature Portfolio Reporting Summary linked to this article.

## Supplementary information


Supplementary InformationSupplementary Tables 1–3, Notes 1 and 2, and Figs. 1–6.
Reporting Summary
Peer Review File
Supplementary Data 1Mass spectrometry of negative-stain TEM analysed Ndh-Ncp from *B. subtilis* fractionation.
Supplementary Data 2Calculation of molar ratios of phospholipids and quinone species in Ndh-Ncp and *E. coli* membranes.
Supplementary Data 3Raw peak intensity data from lipidomics analysis of Ndh-Ncp pre and post *B. subtilis* membrane incubation, *E. coli* and *B. subtilis* membranes, and calculations of lipid intensity ratios.
Supplementary Data 4Genomic survey results of NDH-2 from Bacillota.
Supplementary Video 1Cryo-EM structure of filamentous Ndh-Ncp.
Supplementary Video 2Density found associated with the Ncp tube corresponding to lipids.
Supplementary Video 3Cryo-EM structure of Ndh-Ncp with NADH/MK-8 bound.


## Source data


Source Data Fig. 1Source data.
Source Data Fig. 3Source data.
Source Data Fig. 4Source data.
Source Data Extended Data Fig. 1Unprocessed gels.
Source Data Extended Data Table 1Source data.
Source Data Extended Data Fig. 3Source data and unprocessed gels.
Source Data Extended Data Fig. 4Unprocessed gels.
Source Data Extended Data Table 4Source data.
Source Data Extended Data Fig. 5Source data.
Source Data Extended Data Fig. 6Source data.


## Data Availability

The cryo-EM structures are available in the PDB and EMDB under the codes PDB 9PXK, EMD-71972 (Ndh–Ncp with MK-8/NADH), PDB 9PXL, EMD**-**71973 (Ndh–Ncp) and PDB 9PXM, EMD-71974 (Ndh–Ncp filament). All phylogenetics sequences and accession numbers are found in Supplementary Data File 4. Raw and processed lipidomics data have been deposited in Zenodo at https://doi.org/10.5281/zenodo.21271664 (ref. ^107^). Molecular dynamics simulation data have been deposited in Zenodo at https://doi.org/10.5281/zenodo.21226551 (ref. ^108^). Source data containing raw and uncropped gels and numerical values for all graphs are provided with this paper. All other data are provided with the manuscript.

## References

[1] Szenk, M., Dill, K. A. & de Graff, A. M. Why do fast-growing bacteria enter overflow metabolism? Testing the membrane real estate hypothesis. *Cell Syst.* **5**, 95–104 (2017). 28755958 10.1016/j.cels.2017.06.005

[2] Zhuang, K., Vemuri, G. N. & Mahadevan, R. Economics of membrane occupancy and respiro-fermentation. *Mol. Syst. Biol.* **7**, 500 (2011). 21694717 10.1038/msb.2011.34 PMC3159977

[3] Mannella, C. A. Structure and dynamics of the mitochondrial inner membrane cristae. *Biochim. Biophys. Acta Mol. Cell Res.* **1763**, 542–548 (2006). 10.1016/j.bbamcr.2006.04.006 16730811

[4] Mustárdy, L. & Garab, G. Granum revisited. A three-dimensional model—where things fall into place. *Trends Plant Sci.* **8**, 117–122 (2003). 12663221 10.1016/S1360-1385(03)00015-3

[5] Mühleip, A. et al. Structural basis of mitochondrial membrane bending by the I–II–III2–IV2 supercomplex. *Nature* **615**, 934–938 (2023). 36949187 10.1038/s41586-023-05817-y PMC10060162

[6] Greening, C. & Lithgow, T. Formation and function of bacterial organelles. *Nat. Rev. Microbiol.* **18**, 677–689 (2020). 32710089 10.1038/s41579-020-0413-0

[7] van Niftrik, L. A. et al. The anammoxosome: an intracytoplasmic compartment in anammox bacteria. *FEMS Microbiol. Lett.* **233**, 7–13 (2004). 15098544 10.1016/j.femsle.2004.01.044

[8] Hanson, R. S. & Hanson, T. E. Methanotrophic bacteria. *Microbiol. Rev.* **60**, 439–471 (1996). 8801441 10.1128/mr.60.2.439-471.1996 PMC239451

[9] Zhu, Y. et al. Structure and activity of particulate methane monooxygenase arrays in methanotrophs. *Nat. Commun.* 10.1038/s41467-022-32752-9 (2022). PMC9445010 36064719

[10] Roger, A. J., Muñoz-Gómez, S. A. & Kamikawa, R. The origin and diversification of mitochondria. *Curr. Biol.* **27**, R1177–R1192 (2017). 29112874 10.1016/j.cub.2017.09.015

[11] Rajagopal, M. & Walker, S. in *Protein and Sugar Export and Assembly in Gram-positive Bacteria* (eds Bagnoli. F. & Rappuoli, R.) 1–44 (Springer, 2017).

[12] Rohde, M. The Gram-positive bacterial cell wall. *Microbiol. Spectr.* 10.1128/microbiolspec.gpp3-0044-2018 (2019). PMC11086966 31124431

[13] Kaila, V. R. & Wikström, M. Architecture of bacterial respiratory chains. *Nat. Rev. Microbiol.* **19**, 319–330 (2021). 33437024 10.1038/s41579-020-00486-4

[14] Franza, T. & Gaudu, P. Quinones: more than electron shuttles. *Res. Microbiol.* **173**, 103953 (2022). 35470045 10.1016/j.resmic.2022.103953

[15] Yankovskaya, V. et al. Architecture of succinate dehydrogenase and reactive oxygen species generation. *Science* **299**, 700–704 (2003). 12560550 10.1126/science.1079605

[16] Volbeda, A. et al. Crystal structure of the O_2_-tolerant membrane-bound hydrogenase 1 from *Escherichia coli* in complex with its cognate cytochrome *b*. *Structure* **21**, 184–190 (2013). 23260654 10.1016/j.str.2012.11.010

[17] Liang, Y., Bueler, S. A., Cook, G. M. & Rubinstein, J. L. Structure of mycobacterial NDH-2 bound to a 2-mercapto-quinazolinone inhibitor. *J. Med. Chem.* 10.1021/acs.jmedchem.5c00049 (2025). 40117195

[18] Singh, H., Arentson, B. W., Becker, D. F. & Tanner, J. J. Structures of the PutA peripheral membrane flavoenzyme reveal a dynamic substrate-channeling tunnel and the quinone-binding site. *Proc. Natl Acad. Sci. USA* **111**, 3389–3394 (2014). 24550478 10.1073/pnas.1321621111 PMC3948300

[19] Broc, M. et al. A scaffold for quinone channeling between membrane and soluble bacterial oxidoreductases. *Nat. Struct. Mol. Biol.* 10.1038/s41594-025-01607-4 (2025). 40855134

[20] Grinter, R. et al. Structural basis for bacterial energy extraction from atmospheric hydrogen. *Nature* **615**, 541–547 (2023). 36890228 10.1038/s41586-023-05781-7 PMC10017518

[21] Kropp, A. et al. Quinone extraction drives atmospheric carbon monoxide oxidation in bacteria. *Nat. Chem. Biol.* 10.1038/s41589-025-01836-0 (2025). PMC12202499 39881213

[22] Bouranis, J. A. & Tfaily, M. M. Inside the microbial black box: a redox-centric framework for deciphering microbial metabolism. *Trends Microbiol* **32**, 1170–1178 (2024). 38825550 10.1016/j.tim.2024.05.003

[23] Covarrubias, A. J., Perrone, R., Grozio, A. & Verdin, E. NAD^+^ metabolism and its roles in cellular processes during ageing. *Nat. Rev. Mol. Cell Biol.* **22**, 119–141 (2021). 33353981 10.1038/s41580-020-00313-x PMC7963035

[24] Efremov, R. G., Baradaran, R. & Sazanov, L. A. The architecture of respiratory complex I. *Nature* **465**, 441–445 (2010). 20505720 10.1038/nature09066

[25] Kerscher, S., Dröse, S., Zickermann, V. & Brandt, U. The three families of respiratory NADH dehydrogenases. *Results Probl. Cell Differ.* **45**, 185–222 (2008). 17514372 10.1007/400_2007_028

[26] Blaza, J. N. et al. The mechanism of catalysis by type-II NADH:quinone oxidoreductases. *Sci. Rep.* **7**, 40165 (2017). 28067272 10.1038/srep40165 PMC5220320

[27] Marreiros, B. C., Sena, F. V., Sousa, F. M., Batista, A. P. & Pereira, M. M. Type II NADH:quinone oxidoreductase family: phylogenetic distribution, structural diversity and evolutionary divergences. *Environ. Microbiol.* **18**, 4697–4709 (2016). 27105286 10.1111/1462-2920.13352

[28] Marreiros, B. C. et al. Structural and functional insights into the catalytic mechanism of the Type II NADH:quinone oxidoreductase family. *Sci. Rep.* **7**, 42303 (2017). 28181562 10.1038/srep42303 PMC5299459

[29] Gyan, S., Shiohira, Y., Sato, I., Takeuchi, M. & Sato, T. Regulatory loop between redox sensing of the NADH/NAD^+^ ratio by Rex (YdiH) and oxidation of NADH by NADH dehydrogenase Ndh in *Bacillus subtilis*. *J. Bacteriol.* **188**, 7062–7071 (2006). 17015645 10.1128/JB.00601-06 PMC1636230

[30] Vamshi Krishna, K. & Venkata Mohan, S. Purification and characterization of NDH-2 protein and elucidating its role in extracellular electron transport and bioelectrogenic activity. *Front. Microbiol.* 10.3389/fmicb.2019.00880 (2019). PMC6513898 31133996

[31] Feng, Y. et al. Structural insight into the type-II mitochondrial NADH dehydrogenases. *Nature* **491**, 478–482 (2012). 23086143 10.1038/nature11541

[32] Iwata, M. et al. The structure of the yeast NADH dehydrogenase (Ndi1) reveals overlapping binding sites for water- and lipid-soluble substrates. *Proc. Natl Acad. Sci. USA* **109**, 15247–15252 (2012). 22949654 10.1073/pnas.1210059109 PMC3458368

[33] Sena, F. V. et al. quinone oxidoreductase from *Staphylococcus aureus* has two distinct binding sites and is rate limited by quinone reduction. *Mol. Microbiol.* **98**, 272–288 (2015). 26172206 10.1111/mmi.13120

[34] Heikal, A. et al. Structure of the bacterial type II NADH dehydrogenase: a monotopic membrane protein with an essential role in energy generation. *Mol. Microbiol.* **91**, 950–964 (2014). 24444429 10.1111/mmi.12507

[35] Schägger, H. & Pfeiffer, K. Supercomplexes in th e respiratory chains of yeast and mammalian mitochondria. *EMBO J* **19**, 1777–1783 (2000). 10775262 10.1093/emboj/19.8.1777 PMC302020

[36] Gong, H. et al. An electron transfer path connects subunits of a mycobacterial respiratory supercomplex. *Science* **362**, eaat8923 (2018). 30361386 10.1126/science.aat8923

[37] Yang, Y. et al. Reaction mechanism of single subunit NADH-ubiquinone oxidoreductase (Ndi1) from *Saccharomyces cerevisiae*: evidence for a ternary complex mechanism. *J. Biol. Chem.* **286**, 9287–9297 (2011). 21220430 10.1074/jbc.M110.175547 PMC3059053

[38] Taber, H. W., Dellers, E. A. & Lombardo, L. R. Menaquinone biosynthesis in *Bacillus subtilis*: isolation of men mutants and evidence for clustering of men genes. *J. Bacteriol.* **145**, 321–327 (1981). 6780514 10.1128/jb.145.1.321-327.1981 PMC217275

[39] Dharmaraj, K., Román Silva, J. I., Kahlert, H., Lendeckel, U. & Scholz, F. The acid–base and redox properties of menaquinone MK-4, MK-7, and MK-9 (vitamin K2) in DMPC monolayers on mercury. *Eur. Biophys. J.* **49**, 279–288 (2020). 32372117 10.1007/s00249-020-01433-0 PMC7244470

[40] Nakatani, Y. et al. Unprecedented properties of phenothiazines unraveled by a NDH-2 bioelectrochemical assay platform. *J. Am. Chem. Soc.* **142**, 1311–1320 (2020). 31880924 10.1021/jacs.9b10254

[41] Parks, D. H. et al. GTDB: an ongoing census of bacterial and archaeal diversity through a phylogenetically consistent, rank normalized and complete genome-based taxonomy. *Nucleic Acids Res.* **50**, D785–D794 (2021). 10.1093/nar/gkab776 PMC8728215 34520557

[42] Sena Filipa, V. et al. The two alternative NADH:quinone oxidoreductases from *Staphylococcus aureus*: two players with different molecular and cellular roles. *Microbiol. Spectr.* **12**, e0415223 (2024). 39012110 10.1128/spectrum.04152-23 PMC11302666

[43] Gu, L., Zhao, S., Tadesse, B. T., Zhao, G. & Solem, C. Scrutinizing a *Lactococcus lactis* mutant with enhanced capacity for extracellular electron transfer reveals a unique role for a novel type-II NADH dehydrogenase. *Appl. Environ. Microbiol.* **90**, e0041424 (2024). 38563750 10.1128/aem.00414-24 PMC11107169

[44] Jun, J.-S., Jeong, H.-E., Moon, S.-Y., Shin, S.-H. & Hong, K.-W. Time-course transcriptome analysis of *Bacillus subtilis* DB104 during growth. *Microorganisms* **11**, 1928 (2023). 37630488 10.3390/microorganisms11081928 PMC10458515

[45] Dietrich, H. M. et al. Membrane-anchored HDCR nanowires drive hydrogen-powered CO_2_ fixation. *Nature* **607**, 823–830 (2022). 35859174 10.1038/s41586-022-04971-z

[46] Harwood, C. R. & Cutting, S. M. *Molecular Biological Methods for Bacillus* (Wiley, 1991).

[47] Koo, B.-M. et al. Construction and analysis of two genome-scale deletion libraries for *Bacillus subtilis*. *Cell Syst.* **4**, 291–305.e7 (2017). 28189581 10.1016/j.cels.2016.12.013 PMC5400513

[48] Stavrakakis, M. D., Humphrey, M., van Rij, T., Harwood, C. R. & Strahl, H. Monitoring single cell bioenergetic status and cell lysis in dense and differentiating *Bacillus subtilis* cultures. Preprint at *bioRxiv* 10.1101/2025.08.21.671461 (2025).

[49] Punjani, A., Rubinstein, J. L., Fleet, D. J. & Brubaker, M. A. cryoSPARC: algorithms for rapid unsupervised cryo-EM structure determination. *Nat. Methods* **14**, 290–296 (2017). 28165473 10.1038/nmeth.4169

[50] Zivanov, J. et al. New tools for automated high-resolution cryo-EM structure determination in RELION-3. *eLife* **7**, e42166 (2018). 30412051 10.7554/eLife.42166 PMC6250425

[51] Wagner, T. et al. SPHIRE-crYOLO is a fast and accurate fully automated particle picker for cryo-EM. *Commun. Biol.* **2**, 218 (2019). 31240256 10.1038/s42003-019-0437-z PMC6584505

[52] Bepler, T. et al. Positive-unlabeled convolutional neural networks for particle picking in cryo-electron micrographs. *Nat. Methods* **16**, 1153–1160 (2019). 31591578 10.1038/s41592-019-0575-8 PMC6858545

[53] Abramson, J. et al. Accurate structure prediction of biomolecular interactions with AlphaFold 3. *Nature* **630**, 493–500 (2024). 38718835 10.1038/s41586-024-07487-w PMC11168924

[54] DeLano, W. L. *PyMOL: An Open-source Molecular Graphics Tool* (ScienceOpen, 2002).

[55] Pettersen, E. F. et al. UCSF ChimeraX: structure visualization for researchers, educators, and developers. *Protein Sci.* **30**, 70–82 (2021). 32881101 10.1002/pro.3943 PMC7737788

[56] Afonine, P. V. et al. Real-space refinement in PHENIX for cryo-EM and crystallography. *Acta Crystallogr. D Struct. Biol.* **74**, 531–544 (2018). 29872004 10.1107/S2059798318006551 PMC6096492

[57] Emsley, P. & Cowtan, K. Coot: model-building tools for molecular graphics. *Acta Crystallogr. D Biol. Crystallogr.* **60**, 2126–2132 (2004). 15572765 10.1107/S0907444904019158

[58] Chen, V. B. et al. Molprobity: all-atom structure validation for macromolecular crystallography. *Acta Crystallogr. D* **66**, 12–21 (2010). 20057044 10.1107/S0907444909042073 PMC2803126

[59] Schmid, R. et al. Integrative analysis of multimodal mass spectrometry data in MZmine 3. *Nat. Biotechnol.* **41**, 447–449 (2023). 36859716 10.1038/s41587-023-01690-2 PMC10496610

[60] Kind, T. et al. LipidBlast in silico tandem mass spectrometry database for lipid identification. *Nat. Methods* **10**, 755–758 (2013). 23817071 10.1038/nmeth.2551 PMC3731409

[61] Tsugawa, H. et al. A lipidome atlas in MS-DIAL 4. *Nat. Biotechnol.* **38**, 1159–1163 (2020). 32541957 10.1038/s41587-020-0531-2

[62] Conroy, M. J. et al. LIPID MAPS: update to databases and tools for the lipidomics community. *Nucleic Acids Res.* **52**, D1677–d1682 (2024). 37855672 10.1093/nar/gkad896 PMC10767878

[63] Sud, M. et al. LMSD: LIPID MAPS structure database. *Nucleic Acids Res.* **35**, D527–D532 (2007). 17098933 10.1093/nar/gkl838 PMC1669719

[64] Layre, E. et al. A comparative lipidomics platform for chemotaxonomic analysis of *Mycobacterium tuberculosis*. *Chem. Biol.* **18**, 1537–1549 (2011). 22195556 10.1016/j.chembiol.2011.10.013 PMC3407843

[65] Nitzschke, A. & Bettenbrock, K. All three quinone species play distinct roles in ensuring optimal growth under aerobic and fermentative conditions in *E. coli* K12. *PLoS ONE* **13**, e0194699 (2018). 29614086 10.1371/journal.pone.0194699 PMC5882134

[66] Yasuhiro, K., Yuzuru, A. & Shoshichi, N. Composition and turnover of the phospholipids in *Escherichia coli*. *Biochim. Biophys. Acta* **144**, 382–390 (1967). 4863608

[67] Neidhardt, F., Ingraham, J. & Schaechter, M. *Physiology of the Bacterial Cell: A Molecular Approach* (Sinauer Associates, 1990).

[68] Abraham, M. J. et al. GROMACS: high performance molecular simulations through multi-level parallelism from laptops to supercomputers. *SoftwareX* **1–2**, 19–25 (2015).

[69] Huang, J. et al. CHARMM36m: an improved force field for folded and intrinsically disordered proteins. *Nat. Methods* **14**, 71–73 (2017). 27819658 10.1038/nmeth.4067 PMC5199616

[70] Huang, J. & MacKerell, A. D. Jr. CHARMM36 all-atom additive protein force field: validation based on comparison to NMR data. *J. Comput. Chem.* **34**, 2135–2145 (2013). 23832629 10.1002/jcc.23354 PMC3800559

[71] Klauda, J. B. et al. Update of the CHARMM all-atom additive force field for lipids: validation on six lipid types. *J. Phys. Chem. B* **114**, 7830–7843 (2010). 20496934 10.1021/jp101759q PMC2922408

[72] Feng, S., Wang, R., Pastor, R. W., Klauda, J. B. & Im, W. Location and conformational ensemble of menaquinone and menaquinol, and protein–lipid modulations in archaeal membranes. *J. Phys. Chem. B* **125**, 4714–4725 (2021). 33913729 10.1021/acs.jpcb.1c01930 PMC8379905

[73] Jo, S., Kim, T., Iyer, V. G. & Im, W. CHARMM-GUI: a web-based graphical user interface for CHARMM. *J. Comput. Chem.* **29**, 1859–1865 (2008). 18351591 10.1002/jcc.20945

[74] Humphrey, W., Dalke, A. & Schulten, K. VMD: visual molecular dynamics. *J. Mol. Graph.* **14**, 33–38 (1996). 27-38. 8744570 10.1016/0263-7855(96)00018-5

[75] Jorgensen, W. L., Chandrasekhar, J., Madura, J. D., Impey, R. W. & Klein, M. L. Comparison of simple potential functions for simulating liquid water. *J. Chem. Phys.* **79**, 926–935 (1983).

[76] Beglov, D. & Roux, B. Finite representation of an infinite bulk system: solvent boundary potential for computer simulations. *J. Chem. Phys.* **100**, 9050–9063 (1994).

[77] Olsson, M. H., Søndergaard, C. R., Rostkowski, M. & Jensen, J. H. PROPKA3: consistent treatment of internal and surface residues in empirical p*K*_a_ predictions. *J. Chem. Theory Comput.* **7**, 525–537 (2011). 26596171 10.1021/ct100578z

[78] Bussi, G., Donadio, D. & Parrinello, M. Canonical sampling through velocity rescaling. *J. Chem. Phys.* **126**, 014101 (2007). 17212484 10.1063/1.2408420

[79] Bernetti, M. & Bussi, G. Pressure control using stochastic cell rescaling. *J. Chem. Phys.* **153**, 114107 (2020). 32962386 10.1063/5.0020514

[80] Nosé, S. A molecular dynamics method for simulations in the canonical ensemble. *Mol. Phys.* **52**, 255–268 (1984).

[81] Hoover, W. G. Canonical dynamics: equilibrium phase–space distributions. *Phys. Rev. A Gen. Phys.* **31**, 1695–1697 (1985). 9895674 10.1103/physreva.31.1695

[82] Parrinello, M. & Rahman, A. Polymorphic transitions in single crystals: a new molecular dynamics method. *J. Appl. Phys.* **52**, 7182–7190 (1981).

[83] Allen, M. P., Tildesley, D. J. & Banavar, J. R. Computer simulation of liquids. *Phys. Today* **42**, 105–106 (1989).

[84] Hess, B., Bekker, H., Berendsen, H. J. C. & Fraaije, J. G. E. M. LINCS: a linear constraint solver for molecular simulations. *J. Comput. Chem.* **18**, 1463–1472 (1997).

[85] Verlet, L. Computer “experiments” on classical fluids. I. Thermodynamical properties of Lennard–Jones molecules. *Phys. Rev.* **159**, 98–103 (1967).

[86] Darden, T., York, D. & Pedersen, L. Particle mesh Ewald: an N⋅log(N) method for Ewald sums in large systems. *J. Chem. Phys.* **98**, 10089–10092 (1993).

[87] Phillips, J. C. et al. Scalable molecular dynamics on CPU and GPU architectures with NAMD. *J. Chem. Phys.* **153**, 044130 (2020). 32752662 10.1063/5.0014475 PMC7395834

[88] Hestenes, M. R. & Stiefel, E. Methods of conjugate gradients for solving linear systems. *J. Res. Nat. Bur. Stand.* **49**, 409–436 (1952).

[89] McIntosh, T. J. & Simon, S. A. Area per molecule and distribution of water in fully hydrated dilauroylphosphatidylethanolamine bilayers. *Biochemistry* **25**, 4948–4952 (1986). 3768325 10.1021/bi00365a034

[90] Sanner, M. F., Olson, A. J. & Spehner, J. C. Reduced surface: an efficient way to compute molecular surfaces. *Biopolymers* **38**, 305–320 (1996). 8906967 10.1002/(SICI)1097-0282(199603)38:3%3C305::AID-BIP4%3E3.0.CO;2-Y

[91] O’Reilly, J. E. Oxidation–reduction potential of the ferro-ferricyanide system in buffer solutions. *Biochim. Biophys. Acta* **292**, 509–515 (1973). 4705442 10.1016/0005-2728(73)90001-7

[92] Hyatt, D. et al. Prodigal: prokaryotic gene recognition and translation initiation site identification. *BMC Bioinformatics* **11**, 119 (2010). 20211023 10.1186/1471-2105-11-119 PMC2848648

[93] Kanehisa, M. & Goto, S. KEGG: Kyoto Encyclopedia of Genes and Genomes. *Nucleic Acids Res.* **28**, 27–30 (2000). 10592173 10.1093/nar/28.1.27 PMC102409

[94] Mistry, J. et al. Pfam: the protein families database in 2021. *Nucleic Acids Res.* **49**, D412–D419 (2020). 10.1093/nar/gkaa913 PMC7779014 33125078

[95] Shaffer, M. et al. DRAM for distilling microbial metabolism to automate the curation of microbiome function. *Nucleic Acids Res.* **48**, 8883–8900 (2020). 32766782 10.1093/nar/gkaa621 PMC7498326

[96] Lu, S. et al. CDD/SPARCLE: the conserved domain database in 2020. *Nucleic Acids Res.* **48**, D265–D268 (2019). 10.1093/nar/gkz991 PMC6943070 31777944

[97] Camacho, C. et al. BLAST+: architecture and applications. *BMC Bioinformatics* **10**, 421 (2009). 20003500 10.1186/1471-2105-10-421 PMC2803857

[98] The UniProt Consortium UniProt: the Universal Protein Knowledgebase in 2025. *Nucleic Acids Res.* 53, D609–D617 (2024). 10.1093/nar/gkae1010 PMC11701636 39552041

[99] Buchfink, B., Xie, C. & Huson, D. H. Fast and sensitive protein alignment using DIAMOND. *Nat. Methods* **12**, 59–60 (2015). 25402007 10.1038/nmeth.3176

[100] Chaumeil, P.-A., Mussig, A. J., Hugenholtz, P. & Parks, D. H. GTDB-Tk v2: memory friendly classification with the genome taxonomy database. *Bioinformatics* **38**, 5315–5316 (2022). 36218463 10.1093/bioinformatics/btac672 PMC9710552

[101] Minh, B. Q. et al. IQ-TREE 2: new models and efficient methods for phylogenetic inference in the genomic era. *Mol. Biol. Evol.* **37**, 1530–1534 (2020). 32011700 10.1093/molbev/msaa015 PMC7182206

[102] Steinegger, M. & Söding, J. MMseqs2 enables sensitive protein sequence searching for the analysis of massive data sets. *Nat. Biotechnol.* **35**, 1026–1028 (2017). 29035372 10.1038/nbt.3988

[103] Edgar, R. C. Muscle5: high-accuracy alignment ensembles enable unbiased assessments of sequence homology and phylogeny. *Nat. Commun.* **13**, 6968 (2022). 36379955 10.1038/s41467-022-34630-w PMC9664440

[104] Kalyaanamoorthy, S., Minh, B. Q., Wong, T. K. F., von Haeseler, A. & Jermiin, L. S. ModelFinder: fast model selection for accurate phylogenetic estimates. *Nat. Methods* **14**, 587–589 (2017). 28481363 10.1038/nmeth.4285 PMC5453245

[105] Letunic, I. & Bork, P. Interactive Tree of Life (iTOL) v6: recent updates to the phylogenetic tree display and annotation tool. *Nucleic Acids Res.* **52**, W78–W82 (2024). 38613393 10.1093/nar/gkae268 PMC11223838

[106] Boitreaud, J. et al. Chai-1: decoding the molecular interactions of life. Preprint at *bioRxiv* 10.1101/2024.10.10.615955 (2024).

[107] Lumbantobing, T., Barlow, C., Kropp, A. & Grinter, R. Quinone-transporting filaments extend the respiratory chain of Gram-positive bacteria [Dataset]. *Zenodo*10.5281/zenodo.21271664 (2026).

[108] Simsive, L., Sharma, V. & Zdorevskyi, O. Quinone-transporting filaments extend the respiratory chain of Gram-positive bacteria [Dataset]. *Zenodo*10.5281/zenodo.21226551 (2026).

