# Supplementary material for: Quinone-transporting filaments expand bioenergetic capacity in Gram-positive Bacillota
Source: Nat Microbiol. 2026 Aug 17;11(9):2543–61. doi: 10.1038/s41564-026-02450-z (PMC13524794; doi:10.1038/s41564-026-02450-z)

Extended Data Fig 1d

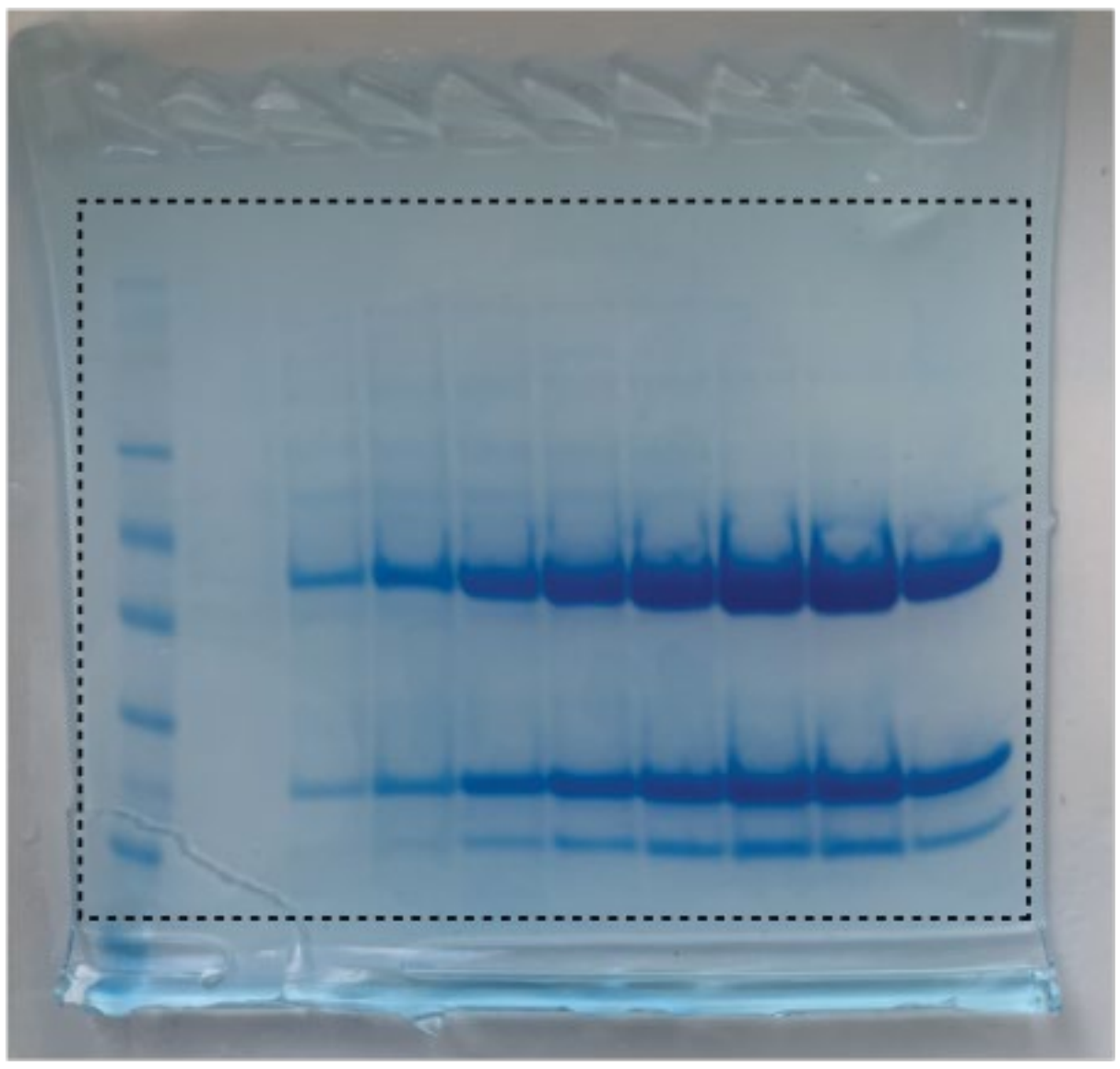

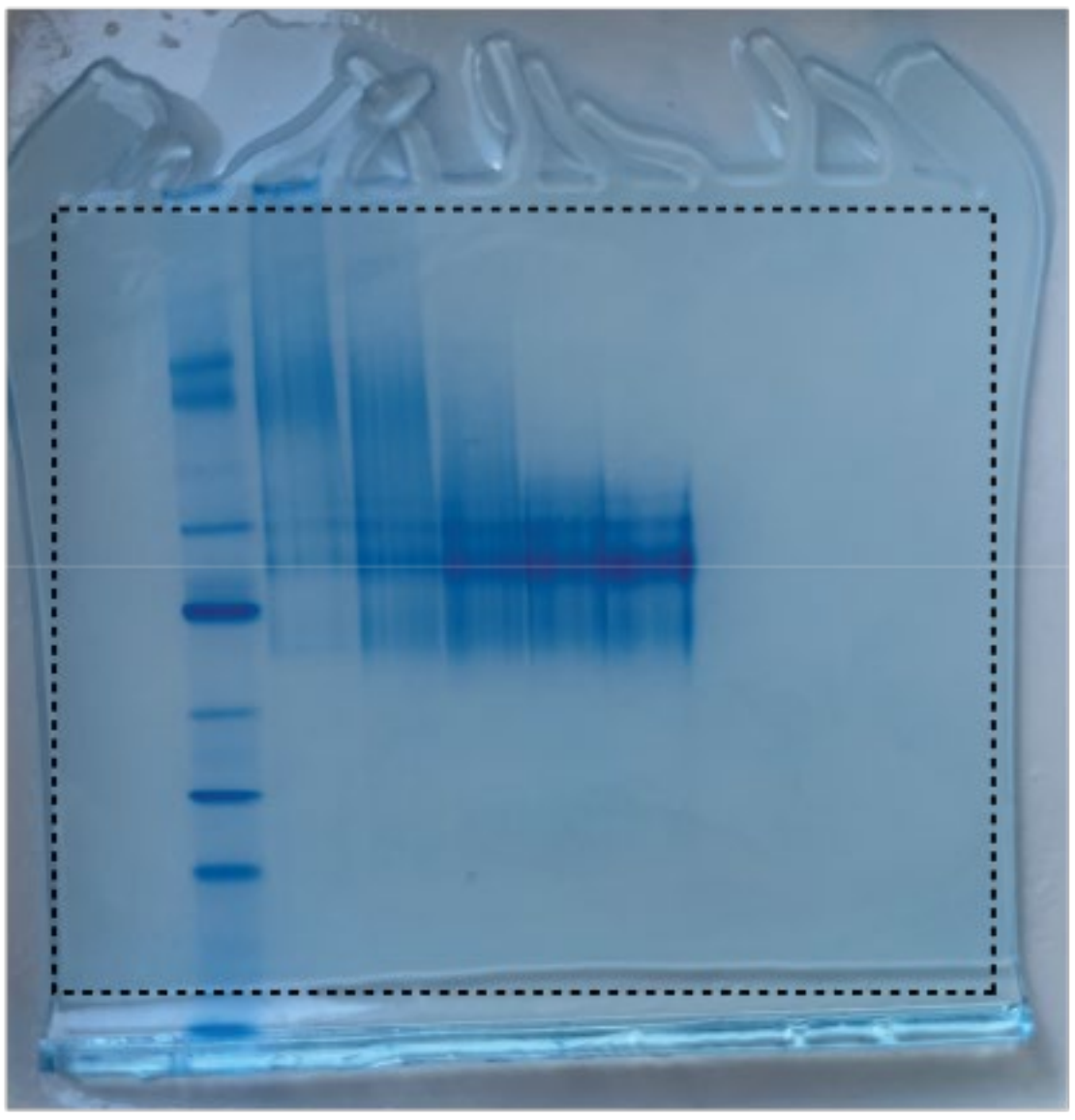

Supplement: Supplementary file 1 [file 41564_2026_2450_MOESM15_ESM.pdf]
