# Supplementary material for: Quinone-transporting filaments expand bioenergetic capacity in Gram-positive Bacillota
Source: Nat Microbiol. 2026 Aug 17;11(9):2543–61. doi: 10.1038/s41564-026-02450-z (PMC13524794; doi:10.1038/s41564-026-02450-z)

Extended Data Fig 3a (and coomassie stained loading control)

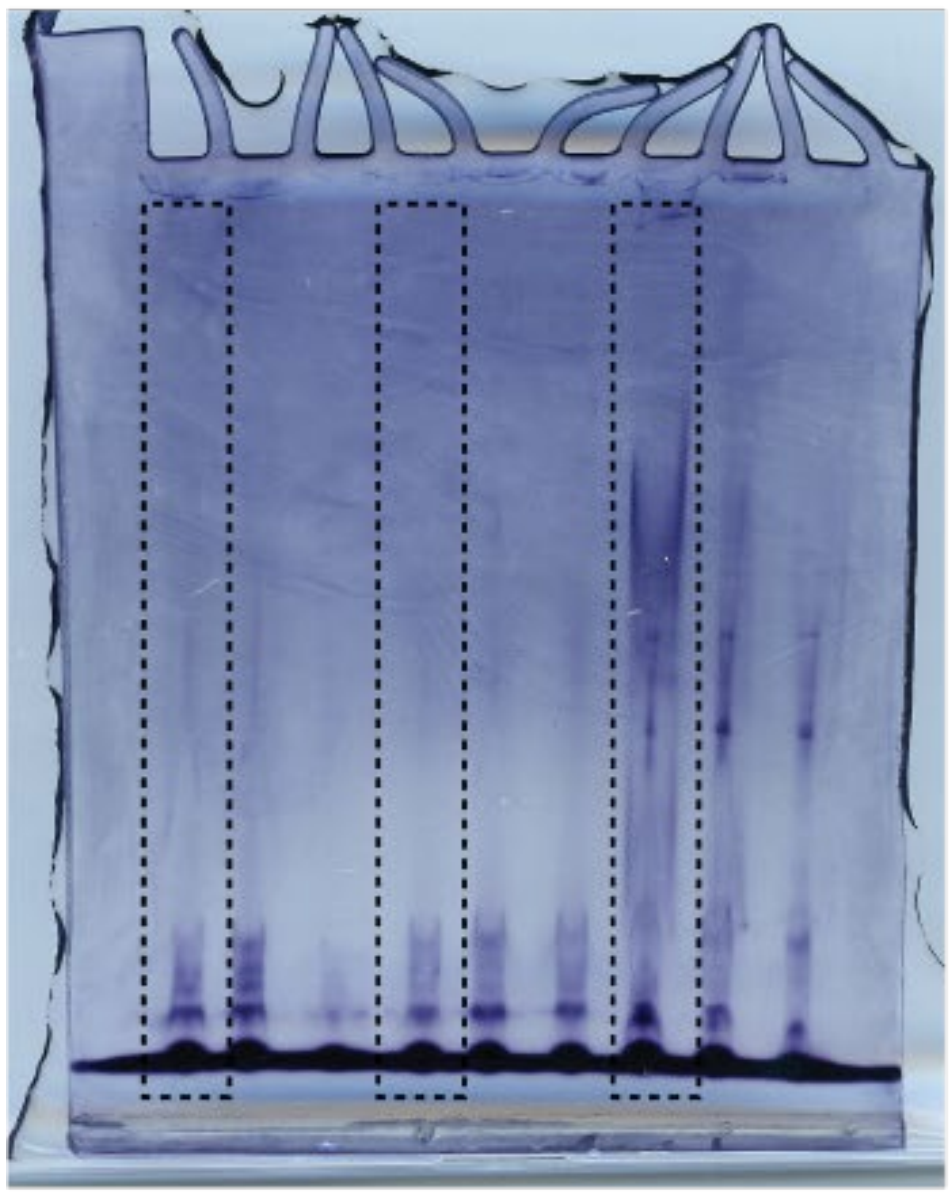

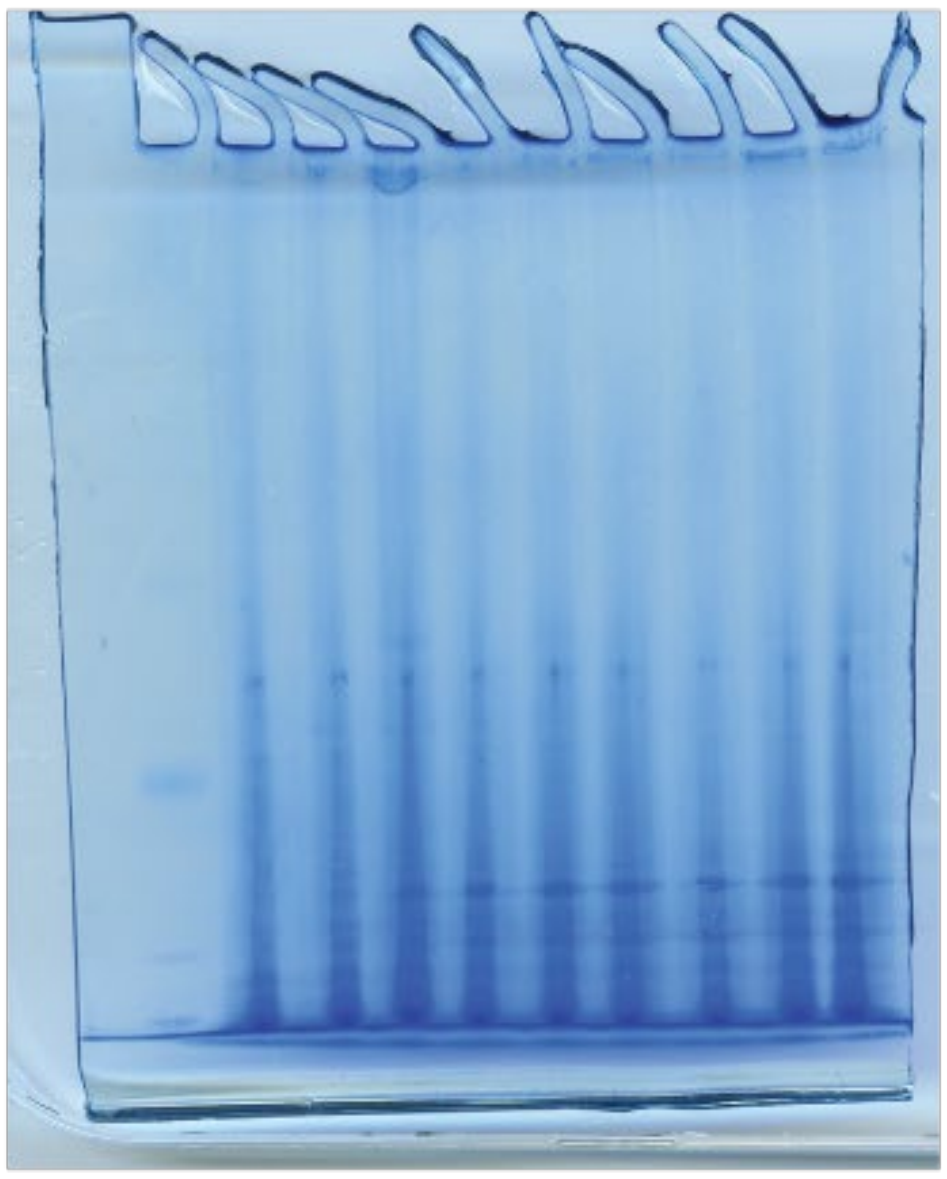

Extended Data Fig 3d

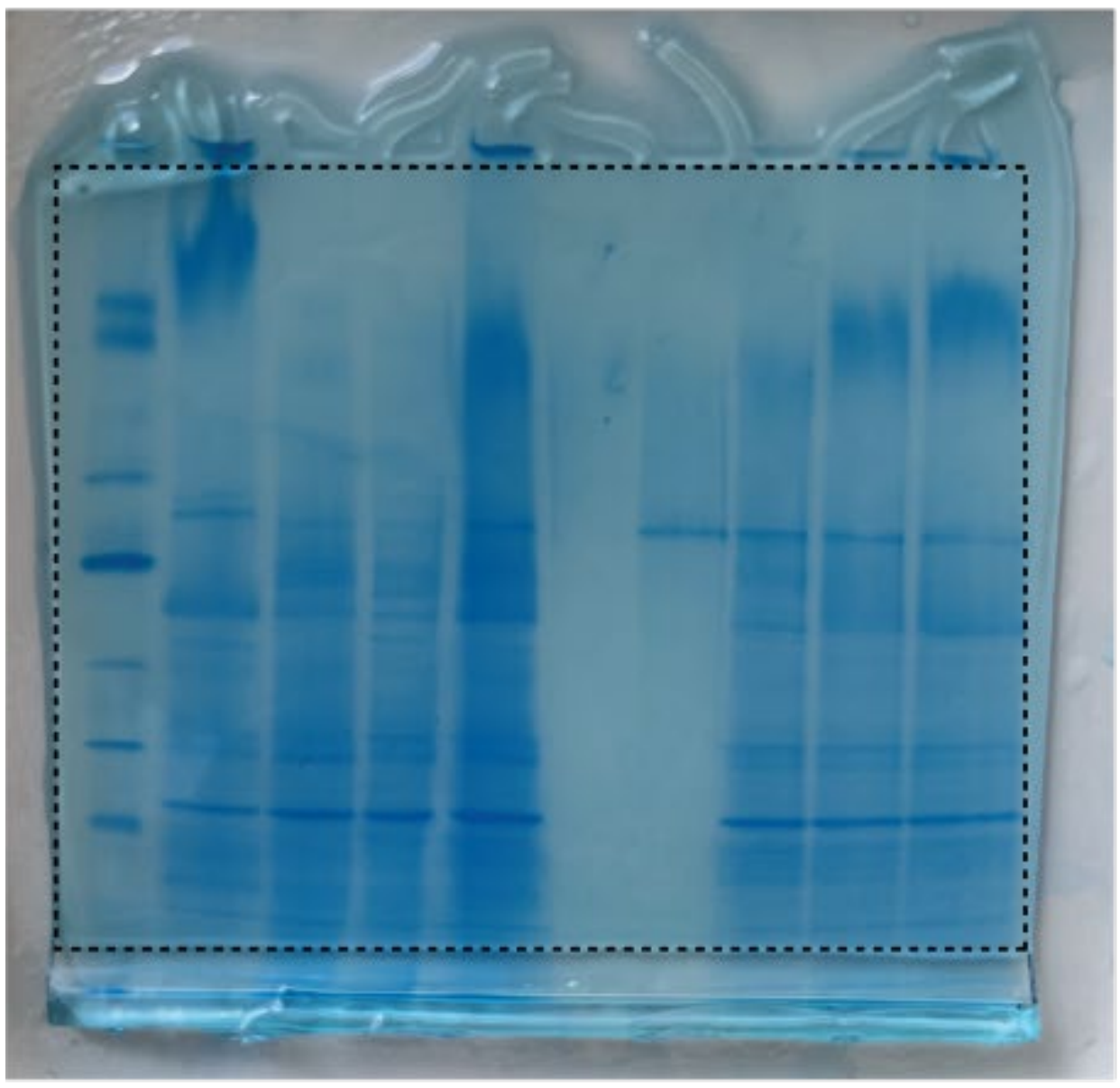

Extended Data Fig 3e

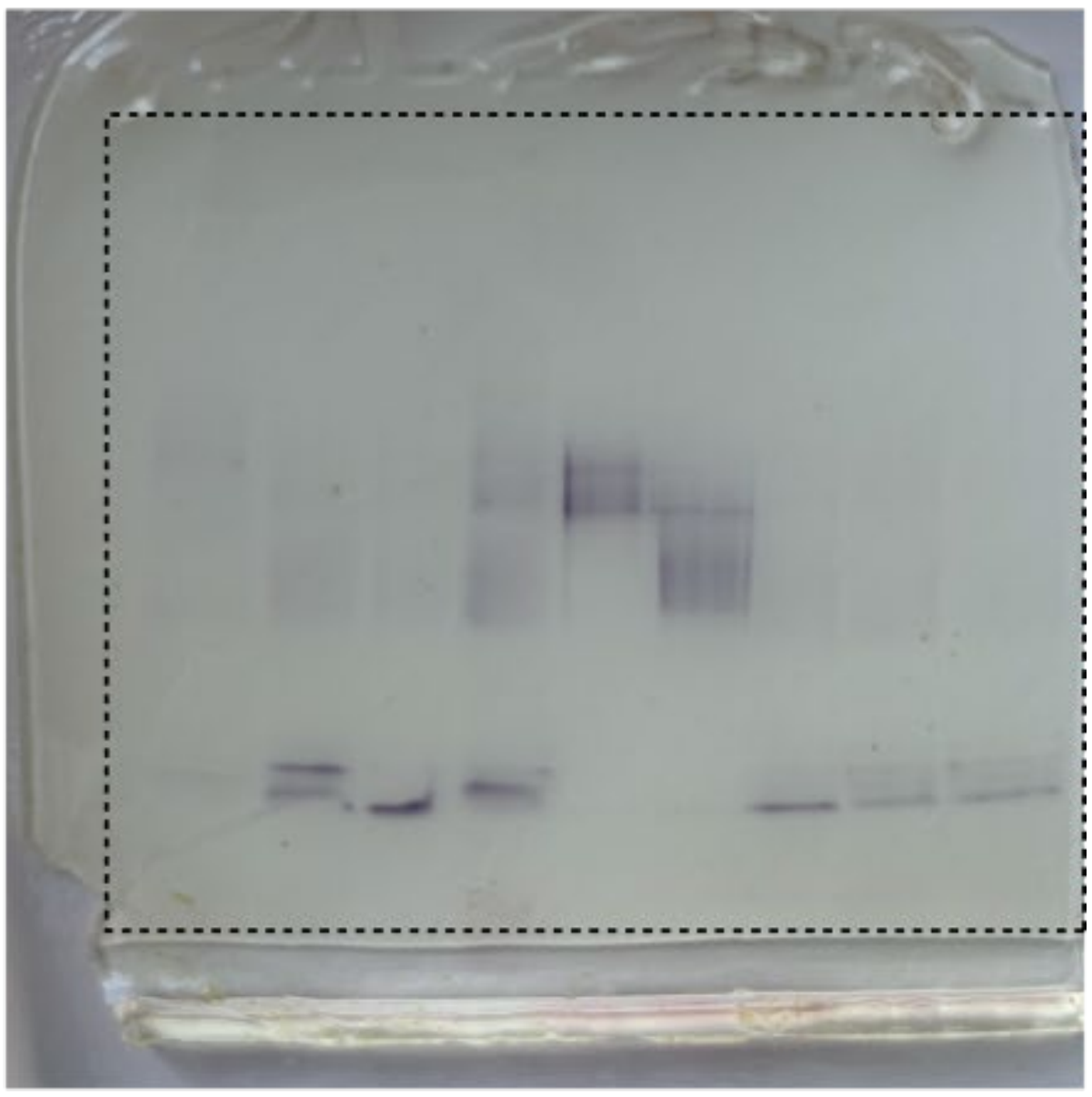

Supplement: Supplementary file 2 [file 41564_2026_2450_MOESM16_ESM.pdf]
