# Supplementary material for: Quinone-transporting filaments expand bioenergetic capacity in Gram-positive Bacillota
Source: Nat Microbiol. 2026 Aug 17;11(9):2543–61. doi: 10.1038/s41564-026-02450-z (PMC13524794; doi:10.1038/s41564-026-02450-z)
Supplement: Supplementary file 4 [file 41564_2026_2450_MOESM1_ESM.pdf]

nature microbiology

Supplementary information

https://doi.org/10.1038/s41564-026-02450-z

Quinone-transporting filaments expand bioenergetic capacity in Gram-positive Bacillota

In the format provided by the authors and unedited

Tables

Supplementary Table S1. Cryo-EM data collection, refinement and validation statistics.

	Ndh-Ncp	*Ndh-Ncp filament*	*Ndh-Ncp NADH/MK-8*
**Data collection and processing**			
Magnification (xk)	130	130	96
Voltage (kV)	300	300	300
Electron exposure (e–/Å^2)	39	39	50
Defocus range (μm)	-1.2-0.6	-1.2-0.6	-1.2-0.6
Pixel size (Å pixel^{-1})	0.655	0.655	0.808
Symmetry imposed	C4	C4	C4
Initial particle images (no.)	483,556	483,556	5,095,952
Final particle images (no.)	356,549	389,955	341,297
Map resolution (Å)	2.11	3.09	2.52
FSC threshold	0.143	0.143	0.143
Map resolution range (Å)	2.1-3.1	2.6-4.6	2.1-4.3
**Refinement**			
Initial model used (PDB code)	-	-	-
Model resolution (Å)	2.11	3.09	2.52
FSC threshold	0.143	0.143	0.143
Model resolution range (Å)	n/a	n/a	n/a
Map sharpening *B* factor (Å^2)			
Model composition			
Non-hydrogen atoms	15,768	46,296	16,084
Protein residues	2,044	6,004	2,044
Ligands	4	12	16
B factors (Å^2)			
Protein	87	156	95
Ligand	65	105	103
R.m.s. deviations			
Bond lengths (Å)	0.002	0.003	0.002
Bond angles (°)	0.486	0.538	0.514
Validation			
MolProbity score	1.16	1.38	1.31
Clashscore	3.71	5.50	5.66
Poor rotamers (%)	0	0.00	0
Ramachandran plot			
Favored (%)	98.57	97.63	98.72
Allowed (%)	1.28	2.37	1.28
Disallowed (%)	0.15	0.00	0.00

Supplementary Table S2. Primers, strains and plasmids used in this study.

Primer Name	Sequence 5'-3'	Purpose
Ndh:ncp Fw	CATCATGGATCCATGCCAGAAACAATCGATCAAACAAATG	Forward primer for amplification of *ncp-ndh* from *B. subtilis*
Ndh:ncp Rv	TATAATGCTAGCGATTAGTAAGCCAGGCTGAAAAGTCC	Reverse primer for amplification of *ncp-ndh* from *B. subtilis*
Mutant1 Fw	GCTGCGCTATTAAAGCCAGAAGTTGCAGCATCATTGACTGTTTTAGTTGACCAG	Forward primer for mutation of pNdh-Ncp to generate Mutant 1
Mutant1 Rv	AATAAGATCTTGCTGGCTTTGG	Reverse primer for mutation of pNdh-Ncp to generate Mutant 1
Mutant2 Fw	GCAAGCGCGGCAGATCTTATTGCTCAGCTATTAAAGCCAGAAGTTCAAG	Forward primer for mutation of pNdh-Ncp to generate Mutant 2
Mutant1 Rv	GCTGACAGACGCATTTGTTTG	Reverse primer for mutation of pNdh-Ncp to generate Mutant 2
**Strain or plasmid**	**Relevant genotype and description**	**Reference or source**
Escherichia coli K12 (DH5α)	Standard laboratory *E. coli* strain for plasmid propagation and maintenance	
Escherichia coli K12 (C41)	Standard laboratory *E. coli* strain for recombinant protein expression	
Bacillus subtilis 168ca (trp+)	Strahl lab strain of *B. subtilis* for genetic modification and WT growth curve	This study
Bacillus subtilis 168ca (trp+) Δ*ndh*::kan	Strahl lab strain of *B. subtilis* with *ndh* deletion	This study
Bacillus subtilis 168ca (trp+) Δ*ncp*::kan	Strahl lab strain of *B. subtilis* with *ncp* deletion	This study
Bacillus subtilis 168	Lyras lab strain of *B. subtilis* for lipidomics experiments	This study
pNdh-Ncp	Ndh-Ncp from *B. subtilis* cloned into a modified pet22b vector which encodes an N-terminal twin strep tag	This study
pNdh-Ncp Mutant1	Ndh-Ncp from *B. subtilis* cloned into a modified pet22b vector which encodes an N-terminal twin strep tag and mutations Q14A, Q16A, Q17A, D21A	This study
pNdh-Ncp Mutant2	Ndh-Ncp from *B. subtilis* cloned into a modified pet22b vector which encodes an N-terminal twin strep tag and mutations D21A, Q22A, Q29A, E30A	This study

Supplementary Table S3. Classical MD simulation setups, their size, time scales, and lipid composition.

Setup	Starting structure PDBID	Number of atoms	Number of lipids in the Ncp chamber	Number of lipids at the end of Ncp tube	Number of MK7	Replica 1 (μs)	Replica 2 (μs)	Replica 3 (μs)
1	9PXL	666,368	40	-	-	1.05	1.17	1.01
2	9PXL	664,518	27	-	-	1.05	1.19	1.06
3	9PXL	665,005	27	5	-	1.01	1.10	1.11
4	9PXL	665,523	27	10	-	0.53	0.58	0.51
5	9PXL	663,776	-	-	-	1.06	1.13	1.13
6*	9PXK	666,394	27	8	4	1.00	1.00	1.00

*in this setup, initial arrangement of chamber lipids was taken from the last snapshot of the production run of setup 2 (replica 1). In the production run of setup 6, we additionally applied distance restraints between NADH-FAD and FAD-MK7 (see methods)

Supplementary Notes

Supplementary note 1. Incubation of the Ndh-Ncp with *B. subtilis* membranes.

To assess how the Ndh–Ncp complex interacts with membranes from its native host, we incubated the purified complex with *B. subtilis* membranes, followed by re-isolation and analysis of its structure and lipid content (Extended Data Fig. 4a). After incubation, membranes were removed by ultracentrifugation, and the complex was subjected to Streptactin affinity purification via its twin-Strep-II tag. Only a minor fraction of the complex bound to the Streptactin resin, with the majority recovered in the flow-through. SDS-PAGE analysis of the flow-through revealed that Ncp had undergone proteolysis during membrane incubation, likely removing the twin-Strep-II tag and preventing resin binding (Extended Data Fig. 4b). This cleaved complex was subsequently re-isolated by size exclusion chromatography, yielding a preparation with >90% of contaminating proteins removed (Extended Data Fig. 4b). Negative stain TEM confirmed that this fraction

contained intact Ndh–Ncp complexes and was largely free of membrane contamination (Extended Data Fig. 4b). This material was used for lipidomic analysis, with this procedure repeated to provide three independent replicates. In contrast, negative stain TEM of the small fraction retained on the Streptactin resin revealed extended Ndh–Ncp filaments that were associated with membrane vesicles (Fig. 3k, Extended Data Fig. 4a,g). Due to the low abundance of this fraction and the presence of membrane contamination, it was not suitable for lipidomic analysis

Supplementary note 2. Preparation of the Ndh-Ncp-phospholipid complex MD simulations. The Ndh-Ncp-phospholipid complex was investigated using classical atomistic molecular dynamics simulations on two cryo-EM structures of the Ndh–Ncp complex with and without substrate. Guided by cryo-EM and lipidomics data, we examined the architecture and dynamics of the lipidic interior of the tube. The FAD, NADH, and MK-7 molecules associated with the complex were restrained during these simulations to generate a mode of the holo-Ndh-Ncp-phospholipid complex (see methods).

Volume estimates suggested the Ndh-Ncp chamber could accommodate ~40–45 lipids. Accordingly, we generated a model with 40 lipids tightly packed into the Ndh-Ncp chamber and analysed its dynamics (Extended Data Fig. 5a,c, setup 1). In this configuration, lipid movement was highly constrained; which is not fully consistent with the blurred cryo-EM density observed in this region, indicating lipid mobility. We therefore constructed an alternative model where we aligned the pre-equilibrated lipid bilayer patch with the centre of the chamber, and retained the lipids populating the tube interior, resulting in 27-lipids bilayer inside the tube (Extended Data Fig. 5a, setup 2). Lipids in this new setup 2 were substantially more mobile than in setup 1 (Extended Data Fig. 5c). During microsecond-scale dynamics of setup 2, lipid head groups and hydration shells migrated toward the protein surface, exposing them to solvent, while water was expelled from the conduit, leading to a hydrophobic environment in the interior of the Ndh-Ncp complex (Extended Data Fig. 5d-f). By contrast, lipids in setup 1 remained largely immobile. These results indicate that the poorly resolved cryo-EM density is best

represented by the smaller number of lipids modelled in setup 2, which we used as the basis for all subsequent simulations.

Simulations also revealed an abrupt conformational change at the N-terminal end of the Ncp tube, where partial collapse closed the opening. This is unlikely to be physiological, especially considering this region is associated with the C-terminal end of a neighbouring Ncp upon filament formation. To prevent this, we introduced five lipids into the region, which stabilised it in a conformation closer to the cryo-EM structure (Extended Data Fig. 5a, setup 3). The addition of these lipids left unoccupied space between the N-terminal region of Ncp and the main chamber of the Ndh-Ncp complex, which we filled with five additional, all lipids oriented tail-first toward the tube end (Extended Data Fig. 5a, setup 4). During equilibration of this model, ~1–2 lipids spontaneously flipped, reorienting their head groups toward solvent while expelling water from the chamber. These simulations resulted in a stable model of the Ndh-Ncp-phospholipid complex, containing 30 lipids in the chamber and 5 at the N-terminal end (Extended Data Fig. 5a, setup 6), which was used for a final round of 1 μs (x3) simulations resulting in the model discussed in this paper.

Supplementary Figures:

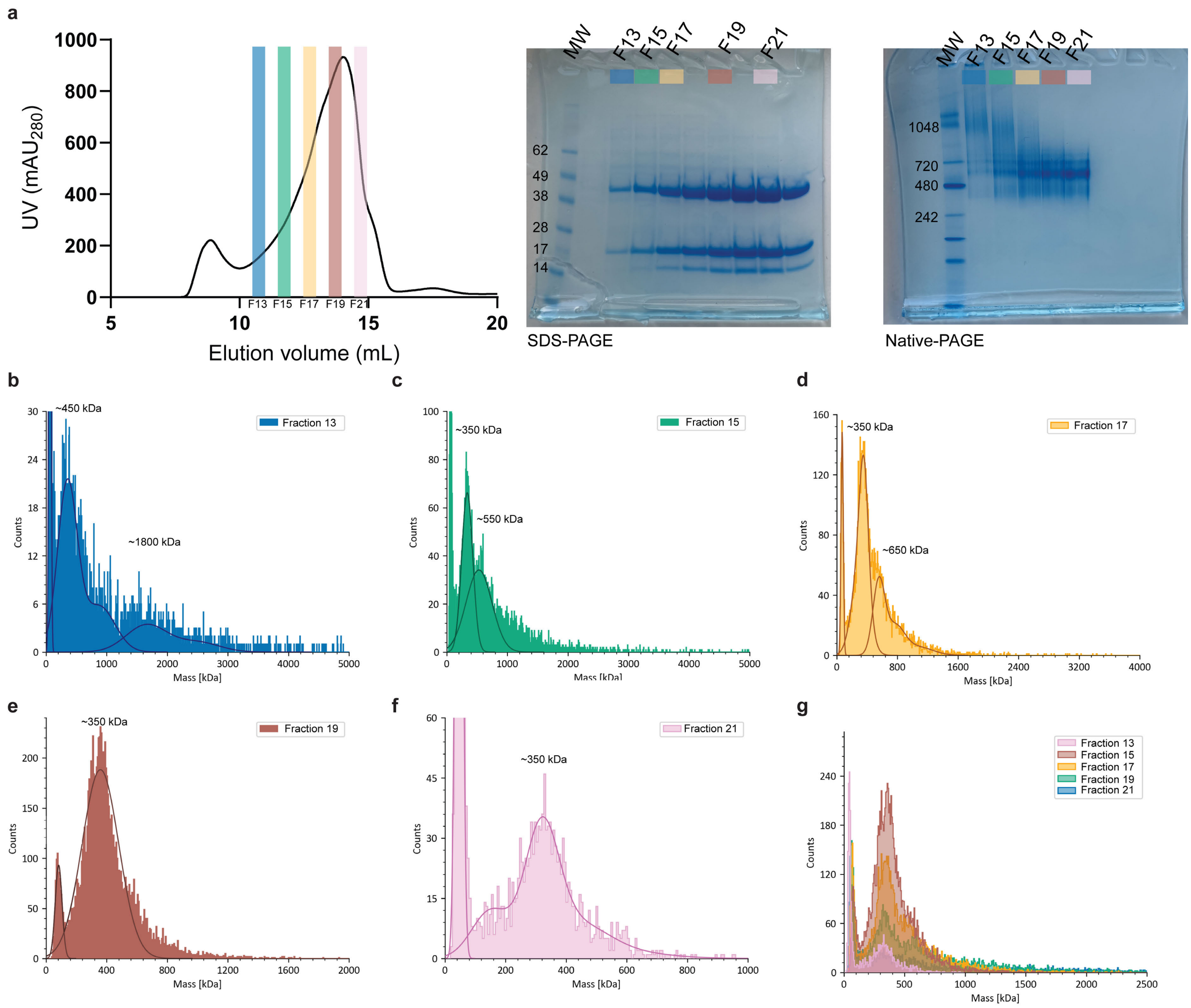

Supplementary Figure 1. Analysis of the size distribution of purified the Ndh-Ndh complex. a, SEC analysis of purified Ndh-Ncp showing fractionation of the extended elution profile, and SDS-PAGE and native-PAGE analysis of these fractions. **b-g,** mass photometry of the fractions from panel a, showing a shift towards lower molecular weight species across the SEC peak.

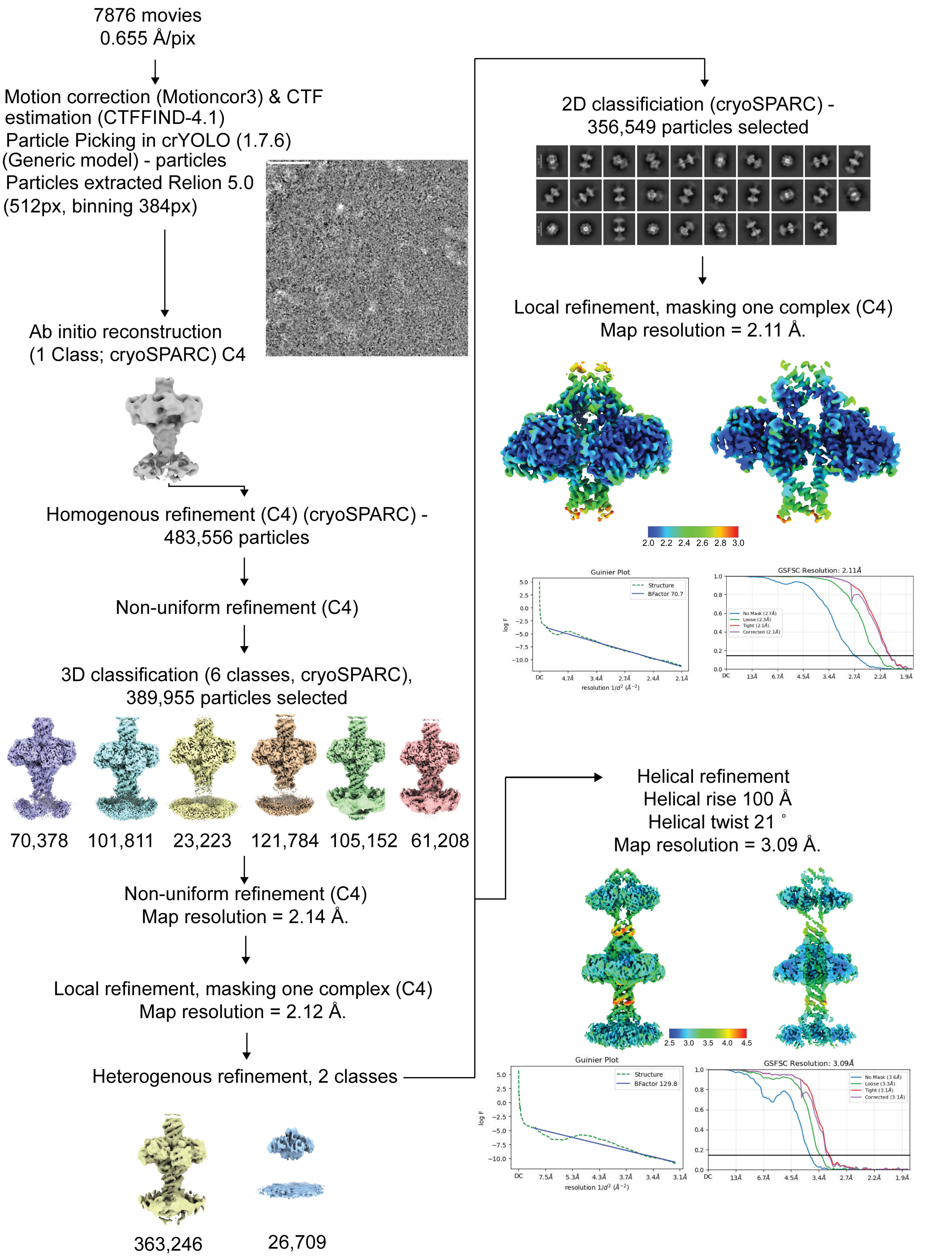

Supplementary Figure 2. Ndh-Ncp cryo-EM data processing workflow. Beginning with 7876 movies, the data was processed in Relion 5.0, crYOLO and cryoSPARC, resulting in two maps, one for the filament at 3.09 Å global resolution and one Ndh-Ncp unit at 2.11 Å global resolution.

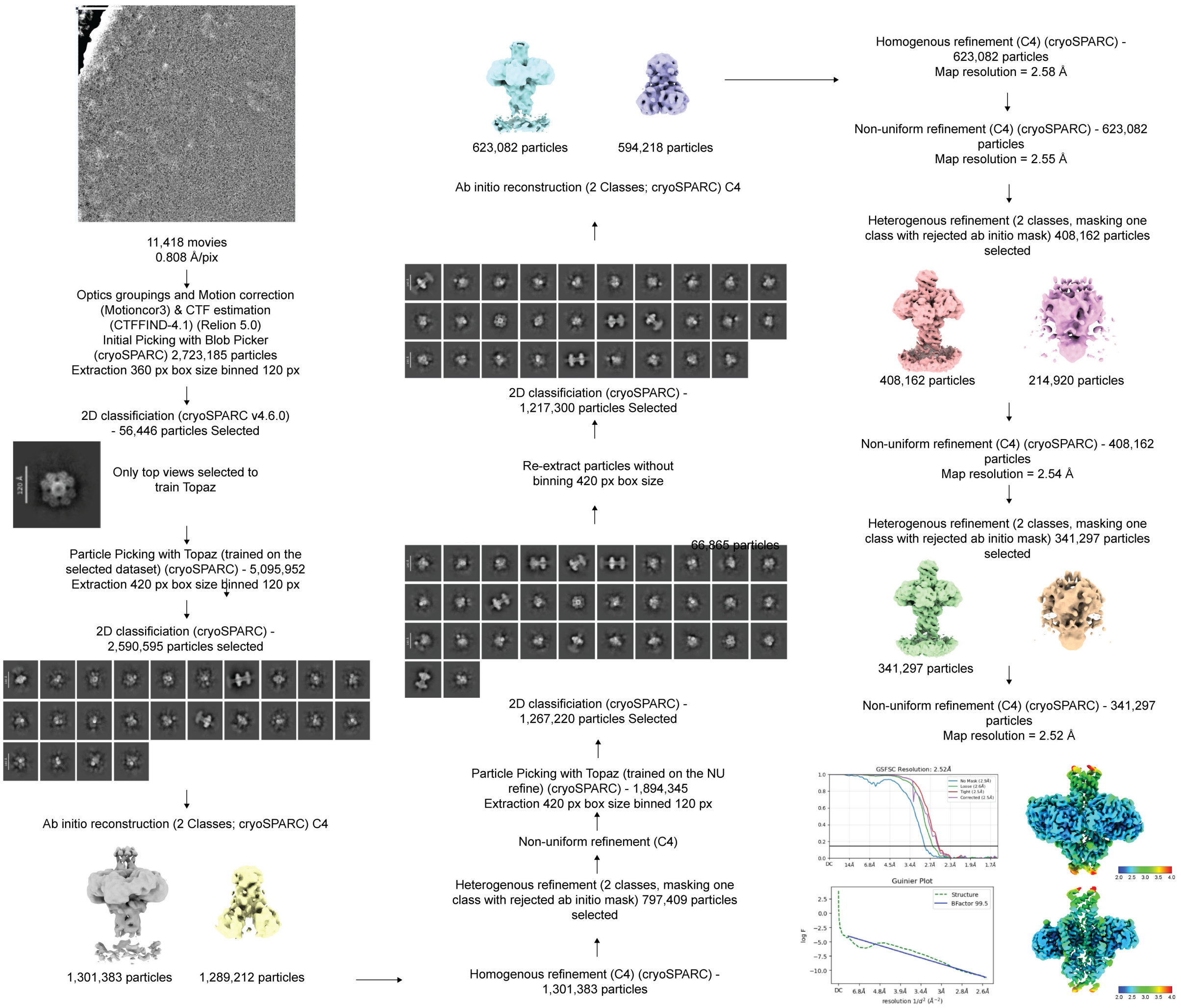

Supplementary Figure 3. Ndh-Ncp NADH-MK-8 data processing workflow. Beginning with 11418 movies, the data was processed in Relion 5.0 and cryoSPARC, resulting in a map of 2.52 Å.

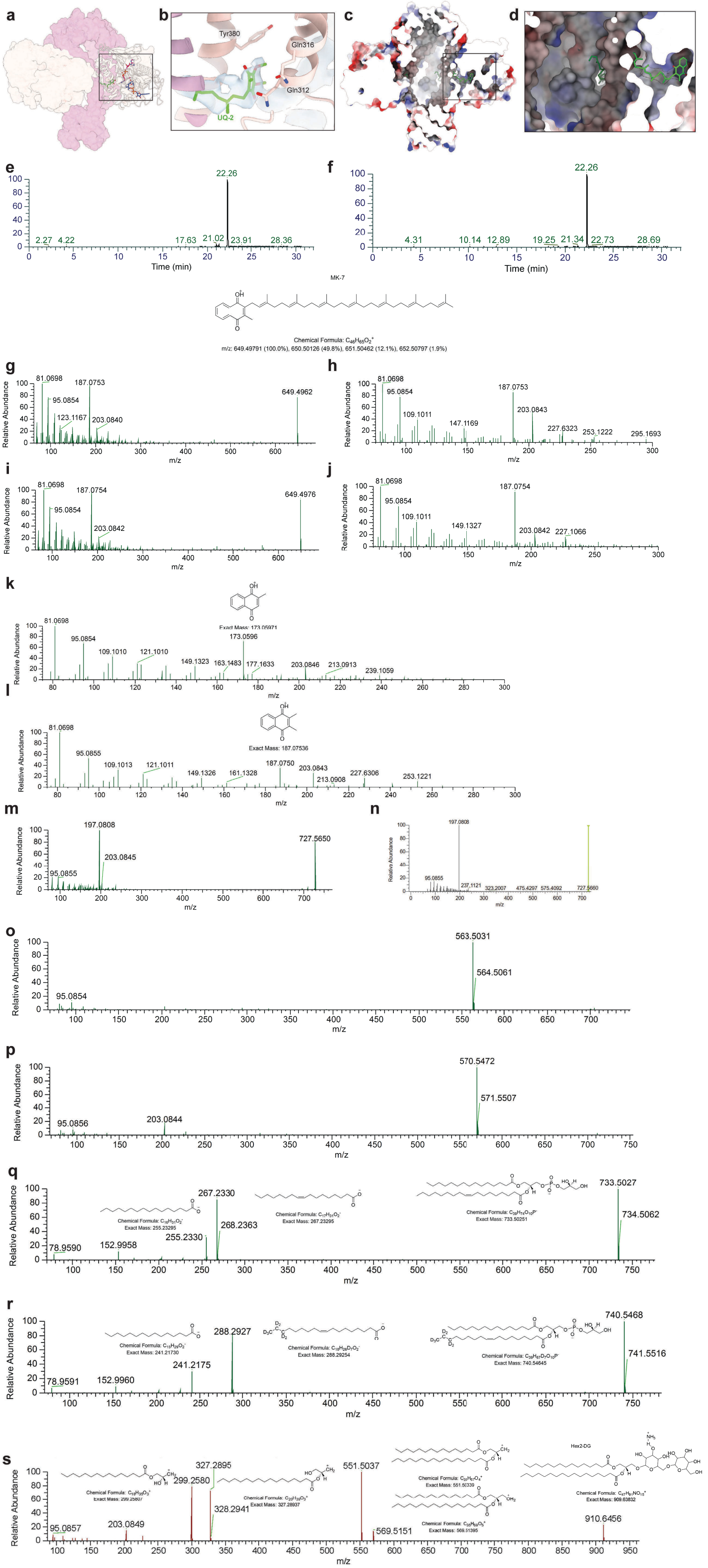

a
b
Tyr380
Gln316
Gln312
UQ-2
c
d
e
22.26
2.27
4.22
17.63
21.02
23.91
28.36
Time (min)
f
22.26
4.31
10.14
12.89
19.25
21.34
22.73
28.69
Time (min)
MK-7
Chemical Formula: $C_{46}H_{65}O_2^+$
m/z: 649.49791 (100.0%), 650.50126 (49.8%), 651.50462 (12.1%), 652.50797 (1.9%)
g
Relative Abundance
81.0698
187.0753
95.0854
123.1167
203.0840
649.4962
m/z
h
Relative Abundance
81.0698
95.0854
109.1011
147.1169
187.0753
203.0843
227.6323
253.1222
295.1693
m/z
i
Relative Abundance
81.0698
187.0754
95.0854
203.0842
649.4976
m/z
j
Relative Abundance
81.0698
95.0854
109.1011
149.1327
187.0754
203.0842
227.1066
m/z
k
Relative Abundance
Exact Mass: 173.05971
81.0698
95.0854
109.1010
121.1010
149.1323
163.1483
173.0596
177.1633
203.0846
213.0913
239.1059
m/z
l
Relative Abundance
Exact Mass: 187.07536
81.0698
95.0855
109.1013
121.1011
149.1326
161.1328
187.0750
203.0843
213.0908
227.6306
253.1221
m/z
m
Relative Abundance
197.0808
203.0845
95.0855
727.5650
m/z
n
Relative Abundance
197.0808
95.0855
237.1121
323.2007
475.4297
575.4092
727.5660
m/z
o
Relative Abundance
563.5031
564.5061
95.0854
m/z
p
Relative Abundance
570.5472
571.5507
95.0856
203.0844
m/z
q
Relative Abundance
Chemical Formula: $C_{16}H_{31}O_2^-$
Exact Mass: 255.23295
Chemical Formula: $C_{17}H_{31}O_2^-$
Exact Mass: 267.23295
Chemical Formula: $C_{39}H_{74}O_{10}P^-$
Exact Mass: 733.50251
267.2330
268.2363
255.2330
152.9958
78.9590
733.5027
734.5062
m/z
r
Relative Abundance
Chemical Formula: $C_{15}H_{29}O_2^-$
Exact Mass: 241.21730
Chemical Formula: $C_{18}H_{28}D_7O_2^-$
Exact Mass: 288.29254
Chemical Formula: $C_{38}H_{65}D_7O_{10}P^-$
Exact Mass: 740.54645
740.5468
741.5516
288.2927
241.2175
152.9960
78.9591
m/z
s
Relative Abundance
Hex2-DG
327.2895
299.2580
328.2941
551.5037
569.5151
95.0857
203.0849
910.6456
Exact Mass: 299.25807
Exact Mass: 327.28937
Exact Mass: 551.50339
Exact Mass: 569.51395
Exact Mass: 909.63832
m/z

Supplementary Figure 4. Ncp quinone and lipid association. a and b, Cartoon representation of Ndh-Ncp with UQ-2 modelled into the quinone binding site (green). Density is shown around UQ-2. **c,** Electrostatic surface representation of Ndh-Ncp cut away down the middle vertically showing the internal tube and the MK-8 molecules interfacing into the tube. **d,** A zoom of a MK-8 molecule (with the tail length truncated based on density) showing the entrance to the Ndh quinone binding site from the Ncp tube. **e-f,** Extracted ion chromatograms for m/z = 649.4960-649.4998 for Ndh-Ncp treated with *B. subtilis* membrane (**e**) and MK-7 standard (**f**). Corresponding MS/MS spectra for the Ndh-Ncp treated with *B. subtilis* membrane (**g and h**) and MK-7 standard (**i and j**). The structure of MK-7 (inset). **k and l**, MS/MS of the feature putatively identified as the ammonium adduct of DMK-8 (m/z = 720.57141) in the Ndh-Ncp treated with *B. subtilis* membrane sample (**k**) and the ammonium adduct of the MK-7 standard (**l**). (Note that in neither case do we observe significant product ions above m/z = 300. The spectra are extremely similar. The two spectra differ in the MK-7 standard containing a product ion at m/z 187.075 consistent with protonated 2,3-dimethyl-1,4-napthoquione which is absent in the putative DMK-8 spectra. Instead, the spectra for the putatively identified DMK-8 contains a product ion at m/z = 173.0597 which is consistent with protonated 2-methylnaphthalene-1,4-dione, the demethylated analogue of 2,3-dimethyl-1,4-napthoquione.) **m and n**, MS/MS spectra for a Ndh-Ncp treated with *B. subtilis* membranes sample at m/z = 788.6346 corresponding to the putatively identified Coenzyme Q8 (UQ-8) (**m**). A screenshot showing the authentic UQ-8 MS/MS spectra from the mzCloud mass spectrometry database (**n**). **o and p**, MS/MS of the protonated ion of PE 33:1 in Ndh-Ncp treated with *B. subtilis* membrane (m/z 703.515206) (**o**) and the protonated ion of the PE 33:1(d7) standard (**p**). In both cases we observe essentially a single product ion corresponding to the loss of the phophosphoethanolamine headgroup. **q and r,** MS/MS of the deprotonated ion of PG 33:1 in Ndh-Ncp treated with *B. subtilis* membrane (m/z 733.5022) (**q**) and the deprotonated ion of the PG 33:1(d7) standard (**r**). **s** MS/MS of the ammonium adduct of the putatively identified Hex2-DG 15:0_17:0. Ndh-Ncp after *B. subtilis* membrane incubation is used as the example for comparison to standards.

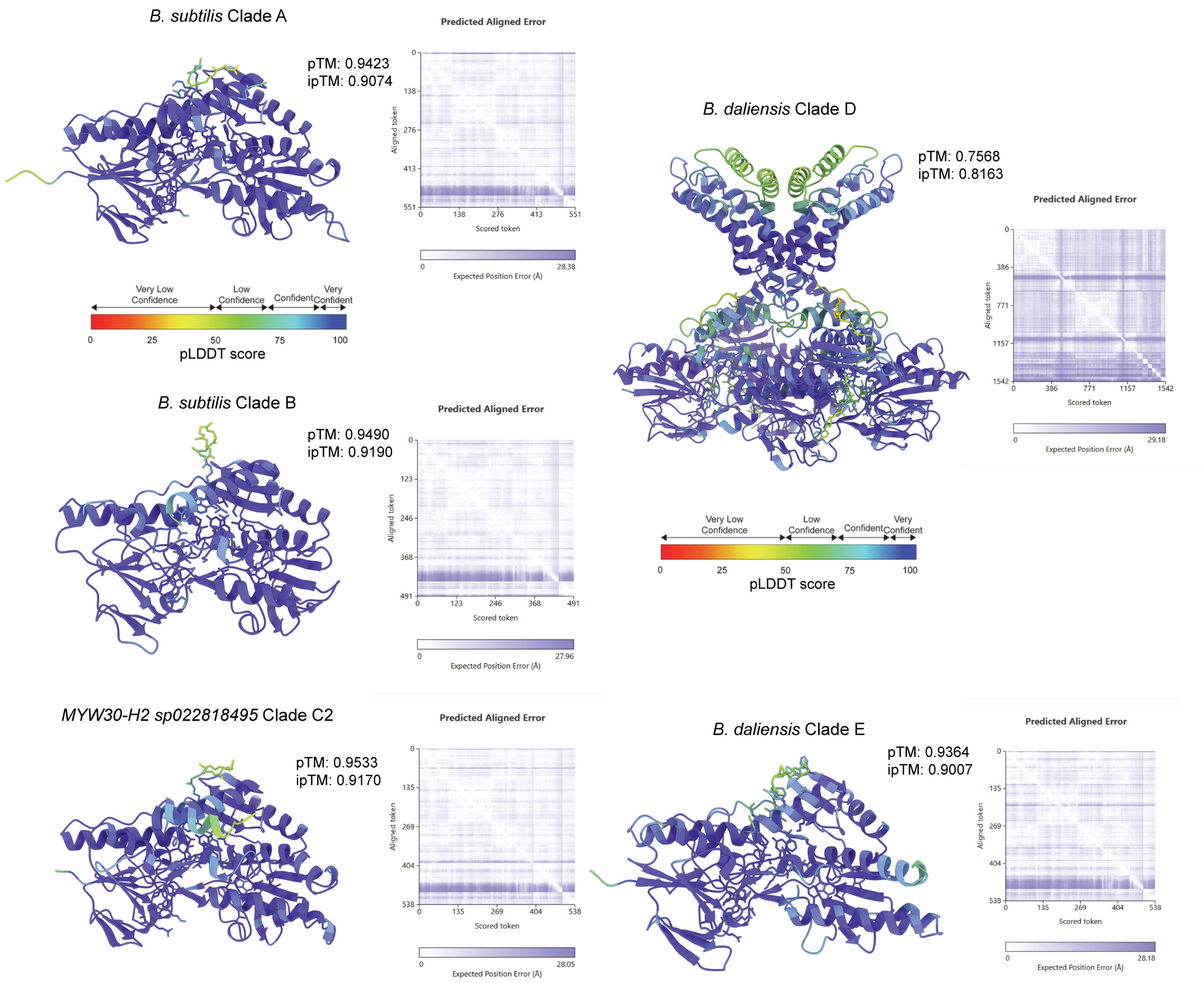

Supplementary Figure 5. Quality estimates for the Chai1 models of NDH-2 enzymes. NDH-2 enzymes modelled in Figure 5 shown here with colour coding to demonstrate model confidence, indicated with the pLDDT key showing confidence levels. Predicted alignment error (PAE) plots are shown on the right of each model, demonstrating the predicted error of the model, with purple showing less error and white higher error.

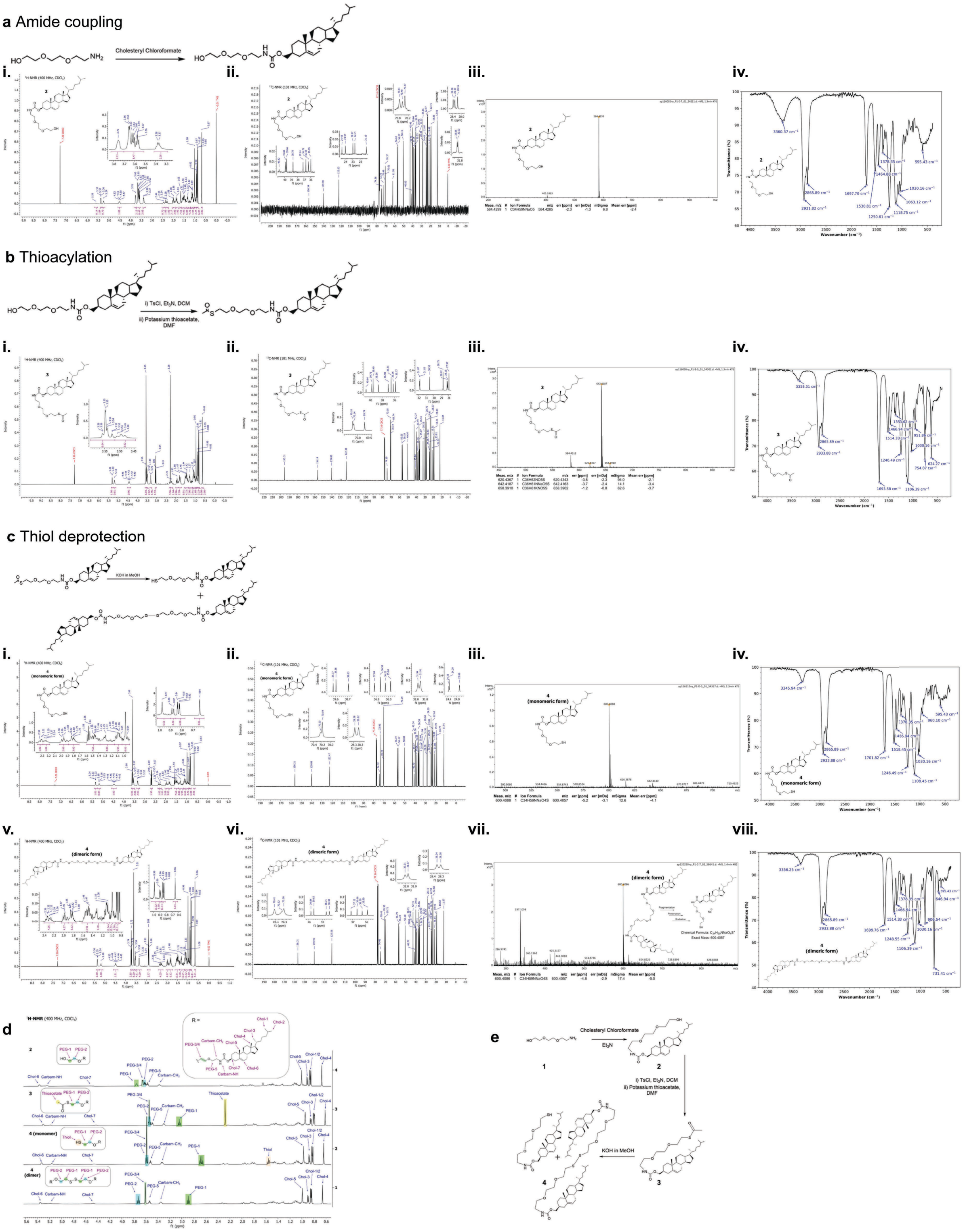

Supplementary Figure 6. Synthesis and characterisation of S-cholesterol for electrode bilayer. a, Amide coupling reaction. **b,** Preparation of Thioester. **c,** Deprotection of Thiol reaction. **d,** Assignment of readily distinguishable regions of the ^{1}H NMR spectra of compounds 2-4. **e,** The full synthesis of EO-3 cholesterol. All **i.** ^{1}H NMR spectrum of each compound, all **ii.** ^{13}C NMR spectrum of each compound, all **iii.** (ESI)HRMS spectrum of each compound, all **iv.** FT-IR (ATR) spectrum of each compound. **cv-viii.** is dimeric form of compound 4.
