# Supplementary material for: Quinone-transporting filaments expand bioenergetic capacity in Gram-positive Bacillota
Source: Nat Microbiol. 2026 Aug 17;11(9):2543–61. doi: 10.1038/s41564-026-02450-z (PMC13524794; doi:10.1038/s41564-026-02450-z)
Supplement: Supplementary file 5 [file 41564_2026_2450_MOESM2_ESM.pdf]

nature portfolio

Corresponding author(s): Rhys Grinter

Last updated by author(s): Jul 9, 2026

Reporting Summary

Nature Portfolio wishes to improve the reproducibility of the work that we publish. This form provides structure for consistency and transparency in reporting. For further information on Nature Portfolio policies, see our Editorial Policies and the Editorial Policy Checklist.

Statistics

For all statistical analyses, confirm that the following items are present in the figure legend, table legend, main text, or Methods section.

n/a	Confirmed	
☐	☒	The exact sample size (n) for each experimental group/condition, given as a discrete number and unit of measurement
☒	☐	A statement on whether measurements were taken from distinct samples or whether the same sample was measured repeatedly
☒	☐	The statistical test(s) used AND whether they are one- or two-sided<br>*Only common tests should be described solely by name; describe more complex techniques in the Methods section.*
☒	☐	A description of all covariates tested
☒	☐	A description of any assumptions or corrections, such as tests of normality and adjustment for multiple comparisons
☐	☒	A full description of the statistical parameters including central tendency (e.g. means) or other basic estimates (e.g. regression coefficient) AND variation (e.g. standard deviation) or associated estimates of uncertainty (e.g. confidence intervals)
☒	☐	For null hypothesis testing, the test statistic (e.g. F, t, r) with confidence intervals, effect sizes, degrees of freedom and P value noted<br>*Give P values as exact values whenever suitable.*
☒	☐	For Bayesian analysis, information on the choice of priors and Markov chain Monte Carlo settings
☒	☐	For hierarchical and complex designs, identification of the appropriate level for tests and full reporting of outcomes
☒	☐	Estimates of effect sizes (e.g. Cohen's d, Pearson's r), indicating how they were calculated

Our web collection on statistics for biologists contains articles on many of the points above.

Software and code

Policy information about availability of computer code

Data collection	EPU v3.6 (Thermo Fisher Scientific) for single-particle cryo-EM data acquisition, Refeyn DiscoverMP for mass photometry, GROMACS 2022.3/4 and NAMD 2.14 for molecular dynamics simulations, Prodigal v2.6.3, DRAM v1.2.4, BLAST+ v2.16.0, DIAMOND v.2.1.10 were used for genome surveys, Thermo Scentific SII for XCalibur v1.8.0.530 and Tune Application v5.1.761.17 for lipidomics, Iviumsoft 4.1148 for electrochemistry
Data analysis	Microsoft Excel 2016, GraphPad Prism 10.6, UCSF MotionCor3, crYOLO (1.7.6), CTFFIND-4.1, RELION 5.0, TOPAZ, AFIS.py, cryoSPARC 4.6.0, UCSF ChimeraX 1.8, Phenix 1.2, Pymol 3.1.8, WinCoot 0.9.8.1, AlphaFold3 (AlphaFold Server), Chai1 (online server) VMD v1.9.4, Openstructure software package, mzMine 4.3, TraceFinder 5.2 SP3, Muscle v5.3, IQ-TREE v2.4.0, iTOL v7, MestReNova 15.0.1-36756 for NMR analysis

For manuscripts utilizing custom algorithms or software that are central to the research but not yet described in published literature, software must be made available to editors and reviewers. We strongly encourage code deposition in a community repository (e.g. GitHub). See the Nature Portfolio guidelines for submitting code & software for further information.

Data

Policy information about availability of data

All manuscripts must include a data availability statement. This statement should provide the following information, where applicable:

- Accession codes, unique identifiers, or web links for publicly available datasets
- A description of any restrictions on data availability
- For clinical datasets or third party data, please ensure that the statement adheres to our policy

The cryo-EM structures are available in the PDB and EMDB under the codes PDB: 9PXK, EMD-71972 (Ndh-Ncp with MK-8/NADH), PDB: 9PXL, EMD-71973 (Ndh-Ncp) and PDB: 9PXM, EMD-71974 (Ndh-Ncp filament). All phylogenetics sequences and accession numbers are found in Supplementary data file S4. Raw and processed lipidomics data have been deposited here: https://doi.org/10.5281/zenodo.21271664 Molecular dynamics simulation data have been deposited here: https://doi.org/10.5281/zenodo.21226551. Source data containing raw and uncropped gels and numerical values for all graphs are published in the Source Data file associated with the manuscript. All other data are provided with the manuscript.

Research involving human participants, their data, or biological material

Policy information about studies with human participants or human data. See also policy information about sex, gender (identity/presentation), and sexual orientation and race, ethnicity and racism.

Reporting on sex and gender	N/A
Reporting on race, ethnicity, or other socially relevant groupings	N/A
Population characteristics	N/A
Recruitment	N/A
Ethics oversight	N/A

Note that full information on the approval of the study protocol must also be provided in the manuscript.

Field-specific reporting

Please select the one below that is the best fit for your research. If you are not sure, read the appropriate sections before making your selection.

☒ Life sciences ☐ Behavioural & social sciences ☐ Ecological, evolutionary & environmental sciences

For a reference copy of the document with all sections, see nature.com/documents/nr-reporting-summary-flat.pdf

Life sciences study design

All studies must disclose on these points even when the disclosure is negative.

Sample size	No statistical methods were used to predetermine sample size. Sample sizes follow established conventions for each experiment type and were chosen to ensure reproducibility while reflecting the large effect sizes observed. For growth assays, lipidomics and activity assays (kinetics, staining and UV- Vis), experiments were conducted in n = 3 independent replicates, with the exception of B. subtilis and E. coli membrane lipidomics that were n = 2 independent replicates. For negative stain TEM micrographs, data were collected with a minimum of 10 independent micrographs, dependent on number of particles within the micrograph (more micrographs were collected for sparsely populated gridholes). For single-particle cryo-EM,no target particle number was predetermined; data were collected to the extent needed to reach high resolution, and final reconstructions used [356,549 / 389,955 / 341,297] particles for the Ndh-Ncp, Ndh-Ncp filament, and Ndh-Ncp NADH/MK-8 structures respectively, yielding gold-standard resolutions of 2.11–3.09 Å sufficient for de novo model building. Molecular dynamics used three independent ~1 µs replicas, sufficient to assess the movement and association of lipids to the complex.
Data exclusions	Optimisation experiments were conducted for activity assays (including electrochemistry, kinetics, UV-Vis spectra and native PAGE activity stain) and these were not included in the final manuscript but are consistent with the data presented.
Replication	Independent biological replicates were used to verify findings, and in some instances orthogonal experiments were used. For cryo-EM structures, substrates were confirmed to bind Ndh-Ncp through UV-Vis, kinetics and electrochemistry experiments. Experiments were conducted with independent biological replicates of n = 3 or n = 2. Two cryo-EM data collections were carried out on independent samples and three structures were refined from independent half-maps and validated by gold-standard FSC (0.143). The Ndh-Ncp multimer and filament formation was supported by analytical size-exclusion, native PAGE and native PAGE activity assays, mutagenesis, mass photometry and native B. subtilis purification and recombinant E. coli purification. Molecular dynamics simulations were carried out n = 3 times as independent simulations. All replication attempts gave consistent results, and no findings failed to reproduce.
Randomization	Randomisation is not relevant to this study. Microbial strains are derived from single clonal cultures and then grown from this culture into independent replicates, so there are no pre-existing differences between samples to require randomisation.

Blinding	Blinding was not relevant to this study. The experiments are mechanistic and biochemical rather than subjective-outcome assessments, and the measurements are quantitative readouts collected by automated instruments (optical density for growth, absorption for kinetics/UV-Vis, mass spectrometry for lipidomics, detector counts for cryo-EM, detector readouts for electrochemistry and mass photometry, that are not susceptible to observer expectation. Computational pipelines for cryo-EM reconstruction and molecular dynamics applied identical, predefined parameters to all conditions, removing scope for selective analysis.<br>conditions, removing scope for selective analysis.

Reporting for specific materials, systems and methods

We require information from authors about some types of materials, experimental systems and methods used in many studies. Here, indicate whether each material, system or method listed is relevant to your study. If you are not sure if a list item applies to your research, read the appropriate section before selecting a response.

Materials & experimental systems

n/a	Involved in the study
☒	☐ Antibodies
☒	☐ Eukaryotic cell lines
☒	☐ Palaeontology and archaeology
☒	☐ Animals and other organisms
☒	☐ Clinical data
☒	☐ Dual use research of concern
☒	☐ Plants

Methods

n/a	Involved in the study
☒	☐ ChIP-seq
☒	☐ Flow cytometry
☒	☐ MRI-based neuroimaging

Plants

Seed stocks	N/A
Novel plant genotypes	N/A
Authentication	N/A
