# Supplementary material for: Quinone-transporting filaments expand bioenergetic capacity in Gram-positive Bacillota
Source: Nat Microbiol. 2026 Aug 17;11(9):2543–61. doi: 10.1038/s41564-026-02450-z (PMC13524794; doi:10.1038/s41564-026-02450-z)
Supplement: Supplementary file 6 [file 41564_2026_2450_MOESM3_ESM.pdf]

nature portfolio

Peer Review File

Quinone-transporting filaments expand bioenergetic capacity in Gram-positive Bacillota

Corresponding Author: Dr Rhys Grinter

Version 0:

Decision Letter:

24th September 2025

Dear Dr Grinter,

Thank you very much for your enquiry about submitting your manuscript "Quinone-transporting filaments extend the respiratory chain of Gram- positive bacteria" to Nature Microbiology. I looked into your full manuscript, and discussed it with my colleague Francois. We think the work is interesting and exciting, and we would definitely send it out for peer review.

If you'd like to take up this option (which we hope you will), please make sure that you update the manuscript files to include any supplementary data or additional files that are important for the reviewers. Please make sure you also provide PDB files (or access to them) for reviewers to take a look into the structural work. Also, you will need to make sure that the information for all of the authors is included in the manuscript handling system. Lastly, we would also need you to fill in a Reporting Summary form, available through this link:
https://www.nature.com/documents/nr-reporting-summary-flat.pdf
You can then also upload this with the submitted manuscript files. Once we have all of this, we can move ahead straight away with securing reviewers.

If you have any questions, please feel free to send me an email. I'll be happy to help. Otherwise, I'll be looking forward to seeing the full submission in my inbox.

In order to submit your complete manuscript to Nature Microbiology, please use the link below:

Link Redacted

Yours sincerely,

Version 1:

Reviewer comments:

Reviewer #1

(Remarks to the Author)
This study reveals the structure of a novel type of NADH dehydrogenase filaments that intriguingly seems to expand the available membrane area for the respiratory chain in gram positive cells. Using complementing cryo-EM, biochemistry, cyclic voltammetry, lipidomics and molecular dynamics analyses the authors show that the Ndh NADH dehydrogenase from Bacillus subtilis interacts with Ncp encoded by a gene adjacent to the ndh gene and the complex forms filaments with an internal tunnel occupied by lipids. The authors show that these filaments are widespread among Bacillota and suggest a plausible recent evolutionary origin of the Ndh-Ncp filmaments through a transitional NDH-2 clade by comparisons of experimental structures and predicted Chai1 models.

The apparent concentration of quinones inside the filament is a major finding and should be emphasised in the manuscript at the same level as the membrane extension. I find that this is a very interesting novel and presumably important functional aspect of microcompartment formation in the electron transport chain.

The study is very elegant, technically sound and it is a significant contribution to the field. I recommend publication in Nature Microbiology after minor revisions as detailed below.

1. The cryo-EM workflow shows that C4 symmetry was applied for the reconstruction. Did the authors do the reconstruction in C1 to investigate if parts of the structure do not obey C4 symmetry (i.e. proteins regions, quinones and lipids)? Were symmetry restraints used for the 2D analysis? This should be discussed in the manuscript. Examples of raw micrographs should be included in the cryo-EM workflow figures.

2. The colouring scheme used in some of the figures (e.g. figure 1b and figure 2a and 2b) makes it difficult to discriminate the curves or chains presented. This should be changed so they appear more distinct.

3. L187 and 1121: Michaelis-Menton should be Michaelis-Menten

4. Materials and Methods section in supplementary: Pixel size should be in Å, and defocus ranges should be in micrometer not micromolar. Were data collected without using an energy filter?

5. L446: gram negative should be gram positive?

6. L519-525 should be moved to the discussion section.

7. Fig 4b: include overlay between MD and cryo-EM position of PE. Was the PE placed in the MD starting structure based on the cryo-EM data?

8. The lipid uptake experiment is very interesting but more information regarding this experiment is needed. I wonder if lipid vesicles were co-purified with the protein filaments and that the apparent lipid uptake/exchange inside the filaments interpreted from the lipidomics data was just caused by contaminating lipids from vesicles. To demonstrate that the interpretation is correct the SEC profiles before and efter incubation should be shown together with SDS-PAGE gels of the protein fractions and negative stain EM (or preferably cryo-EM) should be performed on the SEC fractions similar to those used for lipidomics to show that no vesicles are present in these samples. It would also be interesting to see EM images of the samples with mixed vesicles and Ndh-Ncp filaments to demonstrate direct interaction of the filaments with membranes.

Reviewer #2

(Remarks to the Author)
Aerobic organisms face restrictions on respiratory capacity due to limited membrane surface area. Kopp and coauthors have discovered a complex and lipid-rich protein structure which has evolved to overcome this limitation in a subset of Bacillota/Firmicutes, by serving as an "extension" of membrane-localized respiratory machinery into the cytosol. They support this model with three key pillars: structural data, in vitro electron transfer, and elutional lipid identification. Their work is elegant and convincing in its design and execution, and the manuscript is exceptionally well-written.

Though we have considerable enthusiasm for the novelty and impact of this work, we have major concerns about the lack of replication and detail regarding the lipidomic experiments, on which the central conclusions of this work as presented rely.

Major

The primary conclusion of this work is that the Ndh-Ncp complex is a "quinone-transporting pseudomembrane" that amplifies electron transfer. This conclusion relies on the presence of both quinones and phospholipids within the protein complex, and its relative enrichment of quinones. As cryo-EM data and the resulting models alone do not sufficiently support this finding, the authors performed lipidomics to positively identify quinone and phospholipid species eluted from the Ndh-Ncp complex using organic solvents. Their lipidomic design is wholly appropriate, and the menaquinone species-switching repurification experiment is a creative and convincing method to show specific quinone capture.

However, as specified in the legend for Figure 3 (line 342), the data shown to support this finding are from one experiment with no replication. This is insufficient for publication; the final paper must include at least two high-quality lipid datasets which show consistent results across time and separate protein preparations. This is necessary for both the initial purified sample data and the findings after Bacillus membrane treatment.

The authors also do not sufficiently support their lipid identification. They state in the Methods (line 803) that lipid identities were confirmed by MS/MS, but do not show any of these data except for MK-7 (and its related MK-9 standard) in Supplemental Figure 4e/f. For any lipid identifications that support the major findings of the manuscript, MS/MS spectra should be shown and annotated for diagnostic ions (e.g. the dimethylnapthoquinone ion at m/z 187 in the displayed menaquinone spectra). This includes the identification of MK-7, MK-8, UQ-8, DMK-8, PE, and PG. For phospholipids, spectra for one member of each family would be sufficient (i.e. one PE and one PG species). Formatting for these MS/MS spectra should be modeled on literature examples, e.g. in Reference 72 (Layre et al 2011), and can be confined to the supplement for this manuscript.

A major piece of supporting evidence for the respiratory role of the Ndh-Ncp complex is its enrichment of quinones relative to their levels in E. coli membrane. The authors compare integrated lipid intensities from purified Ndh-Ncp to a "mass balance estimate" for the E. coli quinone to phospholipid ratio (line 309). It is not acceptable to use raw lipid intensities for this purpose since MS molar responses vary greatly between compound classes (even somewhat within classes); estimating molar or mass

ratios of different lipids from mass spectral data requires comparison to lipid standards. The authors could do this by acquiring one commercial standard of similar molecular weight for each relevant lipid family (i.e. one MK, one UQ, one PE, and one PG), and in the same mass spectrometer run establishing standard curves and measuring experimental samples. Alternatively, the authors could bypass the need for standards by measuring relative lipid ion intensities (quinone vs phopholipids) in solvent-extracted membranes from the same E. coli strain used to isolate the complexes, and with this as a reference measure quinone enrichment in the isolated complexes measured under identical conditions (same day, same instrument).

Minor

The purity of the isolated Ndh-Ncp complex is key for interpreting the lipidomics findings, but the only evidence shown of preparation purity is an SDS-PAGE gel in a small inset for Figure S1d. This gel should be expanded to a viewable size and shown in full (not cropped), along with any other relevant supporting data for protein complex purity.

Many figure panels are extremely small in this version of the manuscript, with text and details not discernible on the printed page. The most severe (but not only) examples are Figures 1b, 4d, 5a, S1d/f/g/h, and S4e/f. All text and details in all figures must be understandable when printed on standard size paper, with the potential exception of the phylogenetic trees specifically in the supplement.

DMK-8 is only defined in a figure legend and not the main text

Lipidomic data in the listed repository (line 808) are inaccessible to reviewers

When the Supplemental Data 1 spreadsheet is opened in Excel, it asks the user to sign in with University of Melbourne credentials to update links. The data appear to be intact when this prompt is ignored, but this should be fixed.

Some details are missing from the lipidomics methods and data. Sample injection volume is stated, but not the total resuspension volume from which this injection was withdrawn. Solvent compositions are incomplete (A = 40% isopropanol, 60% ???). It is unclear whether targeted peak areas were taken from mzMine or a different software, and which peak areas were used in Figure 3f/g (the ones in Supplemental Data 1, or others?).

Additional discussion would be helpful regarding why so much ubiquinone signal was observed in Figure 3f, and yet ubiquinone neither fits the molecular model nor is active by kinetics or cyclic voltammetry.

In line 446, “gram-negative” should be “gram-positive”

Reviewer #3

(Remarks to the Author)
The work by Kropp et al describes the characterization of complex composed by the type II NADH dehydrogenase, Ndh-2 and the quinone-transporting protein, Ncp. The genes coding for these proteins were cloned as one continuous fragment, which also codified for an N-terminal twin strep II tag. The complex was produced in E. coli and purified from the cytoplasm. The obtained cryo-EM structure of the Ndh-2 and Ncp complex is amazing. However, evidence for physiological relevance of the assembling of those complexes into filaments is missing.

Major observations:
1- Physiologic relevance of the observed filaments.
The cryo-EM structures show the Ndh-2 and Ncp complexes assembled into filaments containing up to 13 units. The work does not provide evidence for physiological relevance of the existence of such filaments. It is well known and described in literature that many proteins or proteins complexes aggregate forming filaments under certain conditions. The studied complexes were produced in E. coli and obtained from the cytosolic fraction, which is intriguing for a complex that uses a lipid soluble substrate, a quinone.
Such a large filaments should be observed in the cell (B. subtilis) by Cryo-electron tomography.
The hypothesis put forward by the authors that the filaments interact with the membrane transiently is difficult to understand since several quinone/quinol molecules have to have time to interchange to/from the hydrophobic interior of the filament from/to the membrane.
The main problem of the work is the physiological occurrence of the filaments.
How are filaments excluded from being an artifact from heterologous protein production or/and purification procedures? Or larger complexes are better detected in cryo-EM data processing. Did the authors perform an analytic SEC to determine the complex molecular mass or a native electrophoresis? These are important characterizations missing in the manuscript.

2- The putative existence of the filaments does not contribute to expand bioenergetics.
Even if filaments were physiologically relevant, these would not substitute for additional membrane surface area in terms of energy conservation as the case of mitochondrial inner membrane invagination. The filaments would contribute possibly to a higher supply of the lipid soluble substrate, the quinone. This is because Ndh-2 is not involved in energy transduction and no transmembrane potential would be established in the filaments (it is established in the invagination of the mitochondrial inner membrane). The authors should clarify these different concepts.

3- The existence of filaments would make quinol oxidation a limiting factor.
The existence of filaments would allow the accumulation of quinol, the reaction product and quinols may react with O2 producing

radical oxygen species. Thus, it is difficult to understand the reason for a cell to carry such a potential producer of radical oxygen species.

4- Menadione as preferential quinone substrate
Menadione (MDA) is water soluble and the quinone binding site of Ndh-2 is facing the hydrophobic tunnel composed of Ncp subunits. In this case how does MDA interact with Ndh-2? Does it interact at the quinone binding site? If not, what is the relevance of the data obtained with MDA.
Moreover, in order to assess quinone specificity the authors compared the catalytic properties of the reaction using MDA, the redox head group of menaquinone, which has no hydrophobic tail and is thus water soluble, with the reaction using decylubiquinone, a benzoquinone with a tail containing 10 hydrocarbon groups and thus a very hydrophobic molecule. Please comment

5- Structure with substrates
It is not clear how the sample(s) to obtain substrate-bound structure was(were) prepared. On line 244 it is briefly referred "we repeated the cryo-EM, supplementing Ndh-Ncp with
NADH immediately before freezing." (no mention to this in MM section) and then on the following line the authors mention "We resolved this complex at a global resolution of 2.52 A, showing clear density for the FAD group, NADH and quinone, at their structurally conserved binding sites. Where did the quinone come from? Was it added or do the authors hypothesised it was in the tunnel? (based on the lipidomics data the authors mentioned (line 314) "that it is likely that quinones occupy both canonical Ndh quinone binding sites and the central Ndh-Ncp cavity." Although not quinone is observed at its binding site in the structure of the "as prepared" complex).
If quinone was in the tunnel it would be available for reduction by NADH and thus no CTC would be observed. How do the authors conciliate the observation of a CTC upon addition of NADH to the complex when quinone is present? Was the amount of NADH added (how much was it?) higher than that of the quinone? Can the presence of quinone be detected in the UV-Vis spectra? The amount of quinone present may possibly be quantified by the amount of NADH added.
What is the occupancy of NAD+/NADH and of quinone in the cryo-EM structures?

6- MD simulations suggest that the Ndh-Ncp complex tilts so the opening of the Ncp tube penetrates the hydrophobic region of the lipid bilayer. What is the relevance of this tilting? Does the complex have to tilt in order to exchange quinone/quinol with the membrane? How could it be possible in the case of a filament? And only with a transient interaction between the filament and the membrane?

Minor observations:
1- Please refer to type-II NADH dehydrogenase or alternative NADH dehydrogenase always as Ndh-2 and not only Ndh, which refers to NADH dehydrogenase homologous to respiratory complex I.
2- What is the protein concentration used in UV-Vis spectroscopy? What is the protein NADH stoichiometry?
3- The UV-Vis spectrum of the NADH-reduced complex presents a band/shoulder at ~420 nm. What is the origin of this band/shoulder?
4- Clade D – Extracellular electron transfer may be misleading by indicating that those enzymes may be facing the outside of the cell.

Reviewer #4

(Remarks to the Author)

Decision Letter:

28th November 2025

*Please ensure you delete the link to your author homepage in this e-mail if you wish to forward it to your co-authors.

Dear Dr Grinter,

Thank you for your patience while your manuscript "Quinone-transporting filaments extend the respiratory chain of Gram-positive bacteria" was under peer-review at Nature Microbiology. It has now been seen by 3 referees, whose expertise and comments you will find at the end of this email. Although they find your work of potential interest, they have raised a number of concerns that will need to be addressed before we can consider publication of the work in Nature Microbiology.

In particular, Referee #3 noted some concerns that it is not yet clear whether these structures occur in bacteria and whether they expand bioenergetic capacity. Referee #2 noted some concerns over the lipidomics analysis which are also central to the manuscript, and Referee #1 noted a few further experiments that would help solidify the data supporting the main conclusions. It is essential that a revised manuscript address all of these concerns, in particular those from Referee #3, and those pertaining to the lipidomics analysis.

Should further experimental data allow you to address these criticisms, we would be happy to look at a revised manuscript.

We are committed to providing a fair and constructive peer-review process. Please do not hesitate to contact us if there are specific requests from the reviewers that you believe are technically impossible or unlikely to yield a meaningful outcome.

We strongly support public availability of data. Please place the data used in your paper into a public data repository, if one exists, or alternatively, present the data as Source Data or Supplementary Information. If data can only be shared on request, please explain why in your Data Availability Statement, and also in the correspondence with your editor. For some data types, deposition in a public repository is mandatory - more information on our data deposition policies and available repositories can be found at https://www.nature.com/nature-research/editorial-policies/reporting-standards#availability-of-data.

Please include a data availability statement as a separate section after Methods but before references, under the heading "Data Availability". This section should inform readers about the availability of the data used to support the conclusions of your study. This information includes accession codes to public repositories (data banks for protein, DNA or RNA sequences, microarray, proteomics data etc…), references to source data published alongside the paper, unique identifiers such as URLs to data repository entries, or data set DOIs, and any other statement about data availability. At a minimum, you should include the following statement: "The data that support the findings of this study are available from the corresponding author upon request", mentioning any restrictions on availability. If DOIs are provided, we also strongly encourage including these in the Reference list (authors, title, publisher (repository name), identifier, year). For more guidance on how to write this section please see: http://www.nature.com/authors/policies/data/data-availability-statements-data-citations.pdf

If revising your manuscript:

* Include a "Response to referees" document detailing, point-by-point, how you addressed each referee comment. If no action was taken to address a point, you must provide a compelling argument. This response will be sent back to the referees along with the revised manuscript.

* If you have not done so already we suggest that you begin to revise your manuscript so that it conforms to our Article format instructions at http://www.nature.com/nmicrobiol/info/final-submission. Refer also to any guidelines provided in this letter.

* Include a revised version of any required reporting checklist. It will be available to referees (and, potentially, statisticians) to aid in their evaluation if the manuscript goes back for peer review. A revised checklist is essential for re-review of the paper.

When submitting the revised version of your manuscript, please pay close attention to our href="https://www.nature.com/nature-portfolio/editorial-policies/image-integrity">Digital Image Integrity Guidelines. and to the following points below:

-- that unprocessed scans are clearly labelled and match the gels and western blots presented in figures.
-- that control panels for gels and western blots are appropriately described as loading on sample processing controls
-- all images in the paper are checked for duplication of panels and for splicing of gel lanes.

EXTENDED DATA FIGURES

When re-submitting your manuscript, please ensure that any supplementary figures and tables that are crucial to the manuscript's conclusions are converted into Extended Data figures and tables to increase visibility of these data. Extended Data figures and tables are online-only (present in the online PDF and full-text HTML versions of the paper), peer-reviewed display items that provide essential background to the article but are not included in the main article due to space constraints. A maximum of ten Extended Data display items (figures and tables) is permitted.

Finally, please ensure that you retain unprocessed data and metadata files after publication, ideally archiving data in perpetuity, as these may be requested during the peer review and production process or after publication if any issues arise.

Please use the link below to submit a revised paper:

Link Redacted

Note: This url links to your confidential homepage and associated information about manuscripts you may have submitted or be reviewing for us. If you wish to forward this e-mail to co-authors, please delete this link to your homepage first.

Nature Microbiology is committed to improving transparency in authorship. As part of our efforts in this direction, we are now requesting that all authors identified as 'corresponding author' on published papers create and link their Open Researcher and Contributor Identifier (ORCID) with their account on the Manuscript Tracking System (MTS), prior to acceptance. This applies to primary research papers only. ORCID helps the scientific community achieve unambiguous attribution of all scholarly contributions. You can create and link your ORCID from the home page of the MTS by clicking on 'Modify my Springer Nature account'. For more information please visit please visit www.springernature.com/orcid.

If you wish to submit a suitably revised manuscript we would hope to receive it within 6 months. If you cannot send it within this time, please let us know. We will be happy to consider your revision, even if a similar study has been accepted for publication at Nature Microbiology or published elsewhere (up to a maximum of 6 months).

In the meantime we hope that you find our referees' comments helpful.

Yours sincerely,

****
Reviewer Expertise:

Referee #1: CryoEM, ETC
Referee #2: lipidomics, biochemistry
Referee #3: Respiratory chains, energy transduction

Reviewer Comments:

Reviewer #1 (Remarks to the Author):

This study reveals the structure of a novel type of NADH dehydrogenase filaments that intriguingly seems to expand the available membrane area for the respiratory chain in gram positive cells. Using complementing cryo-EM, biochemistry, cyclic voltammetry, lipidomics and molecular dynamics analyses the authors show that the Ndh NADH dehydrogenase from Bacillus subtilis interacts with Ncp encoded by a gene adjacent to the ndh gene and the complex forms filaments with an internal tunnel occupied by lipids. The authors show that these filaments are widespread among Bacillota and suggest a plausible recent evolutionary origin of the Ndh-Ncp filmaments through a transitional NDH-2 clade by comparisons of experimental structures and predicted Chai1 models.

The apparent concentration of quinones inside the filament is a major finding and should be emphasised in the manuscript at the same level as the membrane extension. I find that this is a very interesting novel and presumably important functional aspect of microcompartment formation in the electron transport chain.

The study is very elegant, technically sound and it is a significant contribution to the field. I recommend publication in Nature Microbiology after minor revisions as detailed below.

1. The cryo-EM workflow shows that C4 symmetry was applied for the reconstruction. Did the authors do the reconstruction in C1 to investigate if parts of the structure do not obey C4 symmetry (i.e. proteins regions, quinones and lipids)? Were symmetry restraints used for the 2D analysis? This should be discussed in the manuscript. Examples of raw micrographs should be included in the cryo-EM workflow figures.

2. The colouring scheme used in some of the figures (e.g. figure 1b and figure 2a and 2b) makes it difficult to discriminate the curves or chains presented. This should be changed so they appear more distinct.

3. L187 and 1121: Michaelis-Menton should be Michaelis-Menten

4. Materials and Methods section in supplementary: Pixel size should be in Å, and defocus ranges should be in micrometer not micromolar. Were data collected without using an energy filter?

5. L446: gram negative should be gram positive?

6. L519-525 should be moved to the discussion section.

7. Fig 4b: include overlay between MD and cryo-EM position of PE. Was the PE placed in the MD starting structure based on the cryo-EM data?

8. The lipid uptake experiment is very interesting but more information regarding this experiment is needed. I wonder if lipid vesicles were co-purified with the protein filaments and that the apparent lipid uptake/exchange inside the filaments interpreted from the lipidomics data was just caused by contaminating lipids from vesicles. To demonstrate that the interpretation is correct the SEC profiles before and efter incubation should be shown together with SDS-PAGE gels of the protein fractions and negative stain EM (or preferably cryo-EM) should be performed on the SEC fractions similar to those used for lipidomics to show that no vesicles are present in these samples. It would also be interesting to see EM images of the samples with mixed vesicles and Ndh-Ncp filaments to demonstrate direct interaction of the filaments with membranes.

Reviewer #2 (Remarks to the Author):

Aerobic organisms face restrictions on respiratory capacity due to limited membrane surface area. Kopp and coauthors have discovered a complex and lipid-rich protein structure which has evolved to overcome this limitation in a subset of Bacillota/Firmicutes, by serving as an “extension” of membrane-localized respiratory machinery into the cytosol. They support this model with three key pillars: structural data, in vitro electron transfer, and elutional lipid identification. Their work is elegant and convincing in its design and execution, and the manuscript is exceptionally well-written.

Though we have considerable enthusiasm for the novelty and impact of this work, we have major concerns about the lack of replication and detail regarding the lipidomic experiments, on which the central conclusions of this work as presented rely.

Major

The primary conclusion of this work is that the Ndh-Ncp complex is a “quinone-transporting pseudomembrane” that amplifies electron transfer. This conclusion relies on the presence of both quinones and phospholipids within the protein complex, and its relative enrichment of quinones. As cryo-EM data and the resulting models alone do not sufficiently support this finding, the authors performed lipidomics to positively identify quinone and phospholipid species eluted from the Ndh-Ncp complex using organic solvents. Their lipidomic design is wholly appropriate, and the menaquinone species-switching repurification experiment is a creative and convincing method to show specific quinone capture.

However, as specified in the legend for Figure 3 (line 342), the data shown to support this finding are from one experiment with no replication. This is insufficient for publication; the final paper must include at least two high-quality lipid datasets which show consistent results across time and separate protein preparations. This is necessary for both the initial purified sample data and the findings after Bacillus membrane treatment.

The authors also do not sufficiently support their lipid identification. They state in the Methods (line 803) that lipid identities were confirmed by MS/MS, but do not show any of these data except for MK-7 (and its related MK-9 standard) in Supplemental Figure 4e/f. For any lipid identifications that support the major findings of the manuscript, MS/MS spectra should be shown and annotated for diagnostic ions (e.g. the dimethylnapthoquinone ion at m/z 187 in the displayed menaquinone spectra). This includes the identification of MK-7, MK-8, UQ-8, DMK-8, PE, and PG. For phospholipids, spectra for one member of each family would be sufficient (i.e. one PE and one PG species). Formatting for these MS/MS spectra should be modeled on literature examples, e.g. in Reference 72 (Layre et al 2011), and can be confined to the supplement for this manuscript.

A major piece of supporting evidence for the respiratory role of the Ndh-Ncp complex is its enrichment of quinones relative to their levels in E. coli membrane. The authors compare integrated lipid intensities from purified Ndh-Ncp to a “mass balance estimate” for the E. coli quinone to phospholipid ratio (line 309). It is not acceptable to use raw lipid intensities for this purpose since MS molar responses vary greatly between compound classes (even somewhat within classes); estimating molar or mass ratios of different lipids from mass spectral data requires comparison to lipid standards. The authors could do this by acquiring one commercial standard of similar molecular weight for each relevant lipid family (i.e. one MK, one UQ, one PE, and one PG), and in the same mass spectrometer run establishing standard curves and measuring experimental samples. Alternatively, the authors could bypass the need for standards by measuring relative lipid ion intensities (quinone vs phopholipids) in solvent-extracted membranes from the same E. coli strain used to isolate the complexes, and with this as a reference measure quinone enrichment in the isolated complexes measured under identical conditions (same day, same instrument).

Minor

The purity of the isolated Ndh-Ncp complex is key for interpreting the lipidomics findings, but the only evidence shown of preparation purity is an SDS-PAGE gel in a small inset for Figure S1d. This gel should be expanded to a viewable size and shown in full (not cropped), along with any other relevant supporting data for protein complex purity.

Many figure panels are extremely small in this version of the manuscript, with text and details not discernible on the printed page. The most severe (but not only) examples are Figures 1b, 4d, 5a, S1d/f/g/h, and S4e/f. All text and details in all figures must be understandable when printed on standard size paper, with the potential exception of the phylogenetic trees specifically in the supplement.

DMK-8 is only defined in a figure legend and not the main text

Lipidomic data in the listed repository (line 808) are inaccessible to reviewers

When the Supplemental Data 1 spreadsheet is opened in Excel, it asks the user to sign in with University of Melbourne credentials to update links. The data appear to be intact when this prompt is ignored, but this should be fixed.

Some details are missing from the lipidomics methods and data. Sample injection volume is stated, but not the total resuspension volume from which this injection was withdrawn. Solvent compositions are incomplete (A = 40% isopropanol, 60% ???). It is unclear whether targeted peak areas were taken from mzMine or a different software, and which peak areas were used in Figure 3f/g (the ones in Supplemental Data 1, or others?).

Additional discussion would be helpful regarding why so much ubiquinone signal was observed in Figure 3f, and yet ubiquinone neither fits the molecular model nor is active by kinetics or cyclic voltammetry.

In line 446, “gram-negative” should be “gram-positive”

Reviewer #3 (Remarks to the Author):

The work by Kropp et al describes the characterization of complex composed by the type II NADH dehydrogenase, Ndh-2 and the quinone-transporting protein, Ncp. The genes coding for these proteins were cloned as one continuous fragment, which also codified for an N-terminal twin strep II tag. The complex was produced in E. coli and purified from the cytoplasm. The obtained cryo-EM structure of the Ndh-2 and Ncp complex is amazing. However, evidence for physiological relevance of the assembling of those complexes into filaments is missing.

Major observations:
1- Physiologic relevance of the observed filaments.
The cryo-EM structures show the Ndh-2 and Ncp complexes assembled into filaments containing up to 13 units. The work does not provide evidence for physiological relevance of the existence of such filaments. It is well known and described in literature that many proteins or proteins complexes aggregate forming filaments under certain conditions. The studied complexes were produced in E. coli and obtained from the cytosolic fraction, which is intriguing for a complex that uses a lipid soluble substrate, a quinone.
Such a large filaments should be observed in the cell (B. subtilis) by Cryo-electron tomography.
The hypothesis put forward by the authors that the filaments interact with the membrane transiently is difficult to understand since several quinone/quinol molecules have to have time to interchange to/from the hydrophobic interior of the filament from/to the membrane.
The main problem of the work is the physiological occurrence of the filaments.
How are filaments excluded from being an artifact from heterologous protein production or/and purification procedures? Or larger complexes are better detected in cryo-EM data processing. Did the authors perform an analytic SEC to determine the complex molecular mass or a native electrophoresis? These are important characterizations missing in the manuscript.

2- The putative existence of the filaments does not contribute to expand bioenergetics.
Even if filaments were physiologically relevant, these would not substitute for additional membrane surface area in terms of energy conservation as the case of mitochondrial inner membrane invagination. The filaments would contribute possibly to a higher supply of the lipid soluble substrate, the quinone. This is because Ndh-2 is not involved in energy transduction and no transmembrane potential would be established in the filaments (it is established in the invagination of the mitochondrial inner membrane). The authors should clarify these different concepts.

3- The existence of filaments would make quinol oxidation a limiting factor.
The existence of filaments would allow the accumulation of quinol, the reaction product and quinols may react with O2 producing radical oxygen species. Thus, it is difficult to understand the reason for a cell to carry such a potential producer of radical oxygen species.

4- Menadione as preferential quinone substrate
Menadione (MDA) is water soluble and the quinone binding site of Ndh-2 is facing the hydrophobic tunnel composed of Ncp subunits. In this case how does MDA interact with Ndh-2? Does it interact at the quinone binding site? If not, what is the relevance of the data obtained with MDA.
Moreover, in order to assess quinone specificity the authors compared the catalytic properties of the reaction using MDA, the redox head group of menaquinone, which has no hydrophobic tail and is thus water soluble, with the reaction using decylubiquinone, a benzoquinone with a tail containing 10 hydrocarbon groups and thus a very hydrophobic molecule. Please comment

5- Structure with substrates
It is not clear how the sample(s) to obtain substrate-bound structure was(were) prepared. On line 244 it is briefly referred “we repeated the cryo-EM, supplementing Ndh-Ncp with
NADH immediately before freezing.” (no mention to this in MM section) and then on the following line the authors mention “We resolved this complex at a global resolution of 2.52 A, showing clear density for the FAD group, NADH and quinone, at their structurally conserved binding sites. Where did the quinone come from? Was it added or do the authors hypothesised it was in the tunnel? (based on the lipidomics data the authors mentioned (line 314) “that it is likely that quinones occupy both canonical Ndh quinone binding sites and the central Ndh-Ncp cavity.” Although not quinone is observed at its binding site in the structure of the “as prepared” complex).
If quinone was in the tunnel it would be available for reduction by NADH and thus no CTC would be observed. How do the authors conciliate the observation of a CTC upon addition of NADH to the complex when quinone is present? Was the amount of NADH added (how much was it?) higher than that of the quinone? Can the presence of quinone be detected in the UV-Vis spectra? The amount of quinone present may possibly be quantified by the amount of NADH added.
What is the occupancy of NAD+/NADH and of quinone in the cryo-EM structures?

6- MD simulations suggest that the Ndh-Ncp complex tilts so the opening of the Ncp tube penetrates the hydrophobic region of the lipid bilayer. What is the relevance of this tilting? Does the complex have to tilt in order to exchange quinone/quinol with the membrane? How could it be possible in the case of a filament? And only with a transient interaction between the filament and the membrane?

Minor observations:
1- Please refer to type-II NADH dehydrogenase or alternative NADH dehydrogenase always as Ndh-2 and not only Ndh, which refers to NADH dehydrogenase homologous to respiratory complex I.
2- What is the protein concentration used in UV-Vis spectroscopy? What is the protein NADH stoichiometry?
3- The UV-Vis spectrum of the NADH-reduced complex presents a band/shoulder at ~420 nm. What is the origin of this band/shoulder?
4- Clade D – Extracellular electron transfer may be misleading by indicating that those enzymes may be facing the outside of the cell.

Version 2:

Reviewer comments:

Reviewer #1

(Remarks to the Author)
The authors have successfully responded to my comments and concerns. I congratulate the authors with a beautiful piece of work and significant contribution to the field.

Reviewer #2

(Remarks to the Author)
Comments for the authors

Kropp and coauthors have meaningfully improved their already impressive manuscript with additional experiments and rewriting. These changes constitute a more-than-satisfactory response to both our major and minor concerns. This thorough response also extended to the concerns of other reviewers, many of which we agree with and believe were sufficiently addressed during revision.

We have a few very minor points that could help future reader understanding. The revised manuscript combines Extended Data Figures with Supplemental Figures, which is confusing and challenging to navigate. Extended Data Fig. 4d specifies “DGDG” on its y-axis, an undefined acronym, while the figure legend references Hex2DG. Supplemental Figure 6 is mostly too low-resolution to be interpretable, even when zoomed on a computer screen.

The additional work in this revision has produced a manuscript which we were unabashedly enthusiastic to read, and which we believe will significanty move the field forward with respect to understanding respiration in gram positive bacteria.

Reviewer #3

(Remarks to the Author)
The authors addressed all the points raised by the reviewers, completing their answers with further experiments, which make the work much more robust. Nevertheless, the manuscript would still benefit from the clarification of some points.

Major observations:
1- Physiologic relevance of the observed filaments.
I understand the authors’ arguments and encourage them to introduce these arguments in the Discussion section.

2- The putative existence of the filaments does not contribute to expand bioenergetics.
I understand and agree with the authors’ arguments that “the filaments enhance redox flux”. In fact, in their answer to my concern they mention, “The reviewer is correct that the Ndh–Ncp filaments do not contribute directly to transmembrane potential generation and, in this sense, are not analogous to mitochondrial inner membrane invaginations, which increase the surface area available for proton-pumping complexes.” In this way, the introduction section from lines 50 to 64 should be rewritten accordingly. As it is, it can be misleading.

3- Structure with substrates
It is not clear how much the occupancy of NADH binding sites is. The authors mentioned that “For the NADH-bound structure, Ndh–Ncp (6 mg ml^{-1}) was incubated with 5 μM NADH for 10 min prior to freezing.”. Considering MW=230140 Da, it means that Ndh–Ncp concentration is ~26.07 μM, more than 5 times that of NADH (protein/NADH ration is 1:0.25). Is quinone only observed when NADH is present? The authors mentioned an occupancy of 1 both for NADH and quinone, which is not possible considering the amount of NADH added. How do the authors conciliate these observations? In fact, 25% agrees quite well with the ~22% bulk occupancy inferred from lipidomics. Please discuss.
A discussion on the “curious” establishment of a CTC in the presence of quinone should also be included in the manuscript.

4- MD simulations
I agree with the authors being cautious about the physiological significance of the tilting suggested by the MD simulations. But then what is the relevance of the MD simulations? Why weren’t B. subtilis native lipids considered?

Minor observations:
1- Mutations: Were complementation assays or whole genome sequencing performed?
2- Line 66, Only an integral membrane subunit is needed for these enzymes, the presence of prosthetic groups in these subunits is not always a requirement, such as in the case of complex I, for example. Thus, “containing haem or iron-sulphur clusters“ should be removed.
3- Line 75, What are “high-energy electrons”? Please remove high-energy.
4- Line 98, Please specify the key residues that are missing in B. subtilis Ndh.
5- Line 143, It is FAD not FAD+.
6- Line 153, Please include that DQ has a 10-hydrocarbon tail.
7- Line 213, Please specify the amount of MDA used here.
8- Line 284. Please specify that 5 M of NADH was added.
9- Line 299. The growth conditions of E. coli for the production of Ndh–Ncp ensured a 1:5 liquid:air ration (lines 673 to 675). Are anaerobic conditions established so E. coli can produce menaquinone?
10- Line 1291, extended data fig 7. Please remove the time-course transcriptomic profiling because the growth conditions must be different from the growth performed in the present study, in panel a) from extended data fig 1, the exponential phase is finished by 6 hour and the profiling starts at 8 hour.

11- At least one reference is duplicated.

Reviewer #4

(Remarks to the Author)

Decision Letter:

Our ref: NMICROBIOL-25093515B

26th June 2026

Dear Dr. Grinter,

Thank you for submitting your revised manuscript "Quinone-transporting filaments extend the respiratory chain of Gram-positive bacteria" (NMICROBIOL-25093515B). It has now been seen by the original referees and their comments are below. The reviewers find that the paper has improved in revision, and therefore we'll be happy in principle to publish it in Nature Microbiology, pending minor revisions to satisfy the referees' final requests and to comply with our editorial and formatting guidelines.

If the current version of your manuscript is in a PDF format, please email us a copy of the file in an editable format (Microsoft Word or LaTex)-- we can not proceed with PDFs at this stage.

We are now performing detailed checks on your paper and will send you a checklist detailing our editorial and formatting requirements in a couple of weeks. Please do not upload the final materials and make any revisions until you receive this additional information from us.

Thank you again for your interest in Nature Microbiology Please do not hesitate to contact me if you have any questions.

Sincerely,

Reviewer #1 (Remarks to the Author):

The authors have successfully responded to my comments and concerns. I congratulate the authors with a beautiful piece of work and significant contribution to the field.

Reviewer #2 (Remarks to the Author):

Comments for the authors

Kropp and coauthors have meaningfully improved their already impressive manuscript with additional experiments and rewriting. These changes constitute a more-than-satisfactory response to both our major and minor concerns. This thorough response also extended to the concerns of other reviewers, many of which we agree with and believe were sufficiently addressed during revision.

We have a few very minor points that could help future reader understanding. The revised manuscript combines Extended Data Figures with Supplemental Figures, which is confusing and challenging to navigate. Extended Data Fig. 4d specifies “DGDG” on its y-axis, an undefined acronym, while the figure legend references Hex2DG. Supplemental Figure 6 is mostly too low-resolution to be interpretable, even when zoomed on a computer screen.

The additional work in this revision has produced a manuscript which we were unabashedly enthusiastic to read, and which we believe will significanty move the field forward with respect to understanding respiration in gram positive bacteria.

Reviewer #3 (Remarks to the Author):

The authors addressed all the points raised by the reviewers, completing their answers with further experiments, which make the work much more robust. Nevertheless, the manuscript would still benefit from the clarification of some points.

Major observations:
1- Physiologic relevance of the observed filaments.
I understand the authors’ arguments and encourage them to introduce these arguments in the Discussion section.

2- The putative existence of the filaments does not contribute to expand bioenergetics.
I understand and agree with the authors’ arguments that “the filaments enhance redox flux”. In fact, in their answer to my concern

they mention, “The reviewer is correct that the Ndh–Ncp filaments do not contribute directly to transmembrane potential generation and, in this sense, are not analogous to mitochondrial inner membrane invaginations, which increase the surface area available for proton-pumping complexes.” In this way, the introduction section from lines 50 to 64 should be rewritten accordingly. As it is, it can be misleading.

3- Structure with substrates
It is not clear how much the occupancy of NADH binding sites is. The authors mentioned that “For the NADH-bound structure, Ndh–Ncp (6 mg ml^{-1}) was incubated with 5 μM NADH for 10 min prior to freezing.”. Considering MW=230140 Da, it means that Ndh–Ncp concentration is ~26.07 μM, more than 5 times that of NADH (protein/NADH ration is 1:0.25). Is quinone only observed when NADH is present? The authors mentioned an occupancy of 1 both for NADH and quinone, which is not possible considering the amount of NADH added. How do the authors conciliate these observations? In fact, 25% agrees quite well with the ~22% bulk occupancy inferred from lipidomics. Please discuss.
A discussion on the “curious” establishment of a CTC in the presence of quinone should also be included in the manuscript.

4- MD simulations
I agree with the authors being cautious about the physiological significance of the tilting suggested by the MD simulations. But then what is the relevance of the MD simulations? Why weren’t B. subtilis native lipids considered?

Minor observations:
1- Mutations: Were complementation assays or whole genome sequencing performed?
2- Line 66, Only an integral membrane subunit is needed for these enzymes, the presence of prosthetic groups in these subunits is not always a requirement, such as in the case of complex I, for example. Thus, “containing haem or iron-sulphur clusters“ should be removed.
3- Line 75, What are “high-energy electrons”? Please remove high-energy.
4- Line 98, Please specify the key residues that are missing in B. subtilis Ndh.
5- Line 143, It is FAD not FAD+.
6- Line 153, Please include that DQ has a 10-hydrocarbon tail.
7- Line 213, Please specify the amount of MDA used here.
8- Line 284. Please specify that 5 M of NADH was added.
9- Line 299. The growth conditions of E. coli for the production of Ndh–Ncp ensured a 1:5 liquid:air ration (lines 673 to 675). Are anaerobic conditions established so E. coli can produce menaquinone?
10- Line 1291, extended data fig 7. Please remove the time-course transcriptomic profiling because the growth conditions must be different from the growth performed in the present study, in panel a) from extended data fig 1, the exponential phase is finished by 6 hour and the profiling starts at 8 hour.
11- At least one reference is duplicated.

INTERNAL USE ONLY AIP modules required: ["Life Science"]

Version 3:

Decision Letter:

13th July 2026

Dear Dr Grinter,

I am pleased to accept your Article "Quinone-transporting filaments expand bioenergetic capacity in Gram-positive Bacillota" for publication in Nature Microbiology. Thank you for having chosen to submit your work to us and many congratulations.

Over the next few weeks, your paper will be copyedited to ensure that it conforms to Nature Microbiology style.

Once your paper is typeset, you will receive an email with a link to choose the appropriate publishing options for your paper and our Author Services team will be in touch regarding any additional information that may be required. Once your paper has been scheduled for online publication, the Nature press office will be in touch to confirm the details.

You may wish to make your media relations office aware of your accepted publication, in case they consider it appropriate to organize some internal or external publicity. Once your paper has been scheduled you will receive an email confirming the publication details. This is normally 3-4 working days in advance of publication. If you need additional notice of the date and time of publication, please let the production team know when you receive the proof of your article to ensure there is sufficient time to coordinate. Further information on our embargo policies can be found here:
https://www.nature.com/authors/policies/embargo.html

After the grant of rights is completed, you will receive a link to your electronic proof via email with a request to make any corrections within 48 hours. If, when you receive your proof, you cannot meet this deadline, please inform us at rjsproduction@springernature.com immediately. You will not receive your proofs until the publishing agreement has been

received through our system

Due to the importance of these deadlines, we ask you please us know now whether you will be difficult to contact over the next month. If this is the case, we ask you provide us with the contact information (email, phone and fax) of someone who will be able to check the proofs on your behalf, and who will be available to address any last-minute problems.

Acceptance of your manuscript is conditional on all authors' agreement with our publication policies (see https://www.nature.com/nmicrobiol/editorial-policies). In particular your manuscript must not be published elsewhere.

Authors may need to take specific actions to achieve compliance with funder and institutional open access mandates. If your research is supported by a funder that requires immediate open access (e.g. according to Plan S principles or the NIH public access policy) then you should select the gold OA route, and we will direct you to the compliant route where possible. Because authors warrant under our subscription licensing terms that they haven't committed to licensing any version of their article under a licence inconsistent with the terms of our agreement – including the applicable embargo period – publication under the subscription model isn't suitable for authors whose funders require no embargo.

If you have any questions about our publishing options, costs, Open Access requirements, or our legal forms, please contact ASJournals@springernature.com

An online order form for reprints of your paper is available at https://www.nature.com/reprints/author-reprints.html. All co-authors, authors' institutions and authors' funding agencies can order reprints using the form appropriate to their geographical region.

We welcome the submission of potential cover material (including a short caption of around 40 words) related to your manuscript; suggestions should be sent to Nature Microbiology as electronic files (the image should be 300 dpi at 210 x 297 mm in either TIFF or JPEG format). Please note that such pictures should be selected more for their aesthetic appeal than for their scientific content, and that colour images work better than black and white or grayscale images. Please do not try to design a cover with the Nature Microbiology logo etc., and please do not submit composites of images related to your work. We also can't move forwards wtih AI-generated images. I am sure you will understand that we cannot make any promise as to whether any of your suggestions might be selected for the cover of the journal.

You can now use a single sign-on for all your accounts, view the status of all your manuscript submissions and reviews, access usage statistics for your published articles and download a record of your refereeing activity for the Nature journals.

To assist our authors in disseminating their research to the broader community, our SharedIt initiative provides you with a unique shareable link that will allow anyone (with or without a subscription) to read the published article. Recipients of the link with a subscription will also be able to download and print the PDF.

As soon as your article is published, you will receive an automated email with your shareable link.

With kind regards,

P.S. Click on the following link if you would like to recommend Nature Microbiology to your librarian http://www.nature.com/subscriptions/recommend.html#forms

** Visit the Springer Nature Editorial and Publishing website at www.springernature.com/editorial-and-publishing-jobs for more information about our career opportunities. If you have any questions please click here.**

Open Access This Peer Review File is licensed under a Creative Commons Attribution 4.0 International License, which permits use, sharing, adaptation, distribution and reproduction in any medium or format, as long as you give appropriate credit to the original author(s) and the source, provide a link to the Creative Commons license, and indicate if changes were made.
In cases where reviewers are anonymous, credit should be given to 'Anonymous Referee' and the source.
The images or other third party material in this Peer Review File are included in the article's Creative Commons license, unless indicated otherwise in a credit line to the material. If material is not included in the article's Creative Commons license and your intended use is not permitted by statutory regulation or exceeds the permitted use, you will need to obtain permission directly from the copyright holder.
To view a copy of this license, visit https://creativecommons.org/licenses/by/4.0/

Response to reviewer comments

Reviewer #1 (Remarks to the Author):

This study reveals the structure of a novel type of NADH dehydrogenase filaments that intriguingly seems to expand the available membrane area for the respiratory chain in gram positive cells. Using complementing cryo-EM, biochemistry, cyclic voltammetry, lipidomics and molecular dynamics analyses the authors show that the Ndh NADH dehydrogenase from Bacillus subtilis interacts with Ncp encoded by a gene adjacent to the ndh gene and the complex forms filaments with an internal tunnel occupied by lipids. The authors show that these filaments are widespread among Bacillota and suggest a plausible recent evolutionary origin of the Ndh-Ncp filmaments through a transitional NDH-2 clade by comparisons of experimental structures and predicted Chai1 models.

We thank the reviewer for the time spent reviewing our manuscript.

The apparent concentration of quinones inside the filament is a major finding and should be emphasised in the manuscript at the same level as the membrane extension. I find that this is a very interesting novel and presumably important functional aspect of microcompartment formation in the electron transport chain.

We thank the reviewer for highlighting the potential importance of quinone enrichment within the Ndh–Ncp filament. We agree that this is an interesting functional feature and have strengthened this aspect of the manuscript in the revised version. Specifically, we have now included a quantitative lipidomics comparison between the purified Ndh–Ncp complex and the corresponding membrane fraction. This analysis demonstrates that the quinone-to-phospholipid ratio is significantly higher in the complex (2.4-fold molar enrichment relative to *E. coli* membranes, $p = 0.0053$). Further, following incubation with *B. subtilis* membranes, the peak intensity ratio of MK-7 to a set of *B. subtilis*-specific lipid species is significantly higher in the Ndh-Ncp complex than *B. subtilis* membranes (2-fold, $p = 0.025$). These data provide direct evidence that the complex selectively enriches and sequesters quinones rather than simply reflecting passive association or exchange. We have incorporated these findings into the Results section, and the Discussion notes that this enrichment may enable the filament to function as a localised quinone reservoir or “quinone battery”. At the same time, we have been careful not to overstate this aspect of the mechanism. While the enrichment is statistically significant and

mechanistically informative, the magnitude of the effect is moderate. It does not define the total quinone capacity of the filament or its quantitative contribution to respiratory flux in vivo. For this reason, we consider it most appropriate to present quinone enrichment as an important mechanistic feature of the Ndh–Ncp architecture, rather than a coequal primary finding alongside pseudomembrane formation and surface-area expansion. We believe this balanced framing accurately reflects the strength of the current data while highlighting the functional implications of quinone sequestration.

The study is very elegant, technically sound and it is a significant contribution to the field. I recommend publication in Nature Microbiology after minor revisions as detailed below.

We thank the reviewer for their positive assessment of our manuscript.

1. The cryo-EM workflow shows that C4 symmetry was applied for the reconstruction. Did the authors do the reconstruction in C1 to investigate if parts of the structure do not obey C4 symmetry (i.e. proteins regions, quinones and lipids)? Were symmetry restraints used for the 2D analysis? This should be discussed in the manuscript. Examples of raw micrographs should be included in the cryo-EM workflow figures.

Symmetry restraints were only applied for 3D refinements, specifically ab initio, homogenous and non-uniform refinements (symmetry is stated in the processing workflows when applied, **Supplemental Figures 2 & 3**). In response to the reviewer's suggestion, we have performed processing in C1 symmetry to compare with the density obtained from C4 symmetry processing, and we found that the resulting maps are similar. Of note, there is increased anisotropy due to particle orientation bias in the C1 processed map, which C4 symmetry helps to overcome, significantly improving map quality. In the C1 symmetry processed map, we see higher resolution density for one 'side' of the complex. The quinones, NADH, FAD and lipids are therefore better resolved in these higher resolution regions. **Figure R1,** presented below, shows the maps and lipid density processed in C1 symmetry compared to C4 symmetry. We have not included this in the revised manuscript as we do not believe this comparison is needed for the paper and our conclusions.

A note was added into the methods (**L829**) stating, "Note: Processing of both datasets was also carried out using C1 symmetry for 3D refinement; however, maps from C1 processing were lower

resolution, displayed anisotropy due to orientation bias, and did not reveal additional structural features. As such, subsequent processing focused on C4 symmetry."

We have amended the processing figures to include a sample raw micrograph for each data set (**Supplemental Figure 2 & 3**).

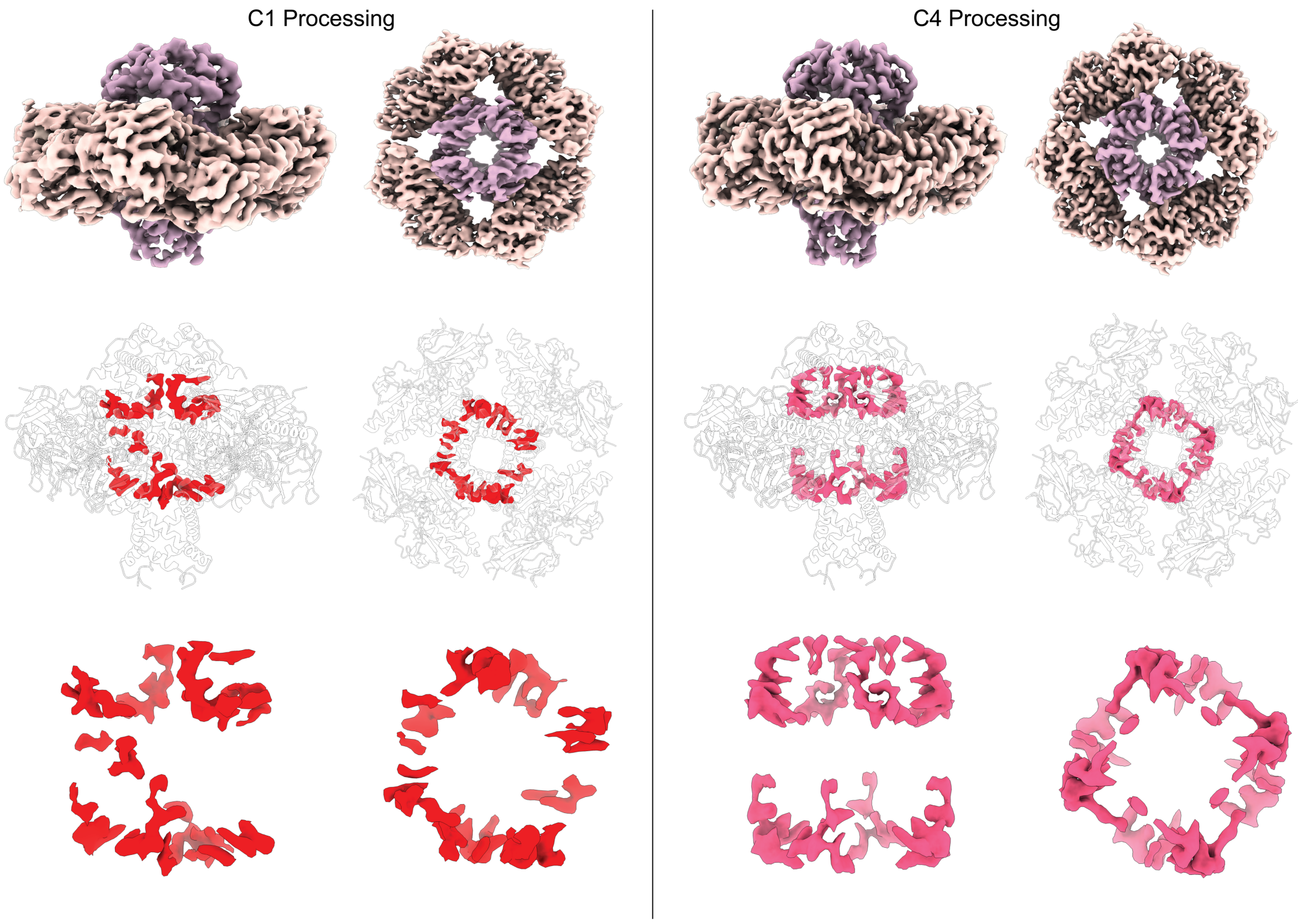

Figure R1. Processing of the Ndh-Ncp complex (quinone and NADH bound) with C1 symmetry applied (left) or C4 symmetry applied (right). Top panel shows the maps after processing. Middle panel shows the cartoon of the complex in transparent white, with difference maps shown in red (C1) or dark pink (C4) of the lipid density. Bottom panel shows difference maps of the lipid density without the cartoon of the complex.

2. The colouring scheme used in some of the figures (e.g. figure 1b and figure 2a and 2b) makes it difficult to discriminate the curves or chains presented. This should be changed so they appear more distinct.

We have amended these figures to make them easier to interpret.

3. L187 and 1121: Michaelis-Menton should be Michaelis-Menten

We have corrected this in the revised manuscript.

4. Materials and Methods section in supplementary: Pixel size should be in Å, and defocus ranges should be in micrometer not micromolar. Were data collected without using an energy filter?

We have corrected these points in the revised manuscript. Data for the apo-Ndh-Ncp complex were collected with an energy filter, while data for the Ndh-Ncp-NADH structure were collected without an energy filter.

5. L446: gram negative should be gram positive?

Amended.

6. L519-525 should be moved to the discussion section.

We have removed this point from the results and incorporated it into the discussion in the revised manuscript.

7. Fig 4b: include overlay between MD and cryo-EM position of PE. Was the PE placed in the MD starting structure based on the cryo-EM data?

Thank you for this suggestion. The instinct to directly overlay the MD-derived lipid positions with the cryo-EM density is sound, and we attempted this during our analysis. However, there are two key limitations that preclude a meaningful comparison. First, the Ndh–Ncp complex exhibits intrinsic flexibility, and the MD simulations capture a relaxed ensemble that deviates modestly from the experimental structure (global RMSD ~2–3 Å). As a result, individual MD snapshots do not align precisely with the cryo-EM reconstruction, making a one-to-one structural overlay unreliable. Second, the phospholipid density observed in the cryo-EM map represents an ensemble average over multiple lipid conformations and occupancies, rather than the position of a single, well-defined molecule. In contrast, the MD simulations resolve discrete lipid trajectories that remain highly dynamic over time. Consequently, attempting to match any individual lipid from the MD simulations to the cryo-EM density is not physically meaningful in

this case. For these reasons, we consider that a direct overlay would risk overinterpretation of both datasets. Instead, we present the cryo-EM lipid density with part of a PE molecule modelled for reference (**Figure 2e-g**) and the MD-derived lipid positions (**Figure 4**) separately, with **Figure 4b** providing a residue-level comparison that highlights consistent spatial relationships between the protein and phospholipids across both approaches. The lipid placement in the MD simulations presented in the main text was not guided by the cryo-EM density. Lipids were initially positioned in the central cavity and allowed to relax during equilibration, with the resulting distributions emerging from microseconds of production simulations. Details of this procedure are provided in **Supplemental Note 2**, **Extended Data Figure 5** and the Methods.

8. The lipid uptake experiment is very interesting but more information regarding this experiment is needed. I wonder if lipid vesicles were co-purified with the protein filaments and that the apparent lipid uptake/exchange inside the filaments interpreted from the lipidomics data was just caused by contaminating lipids from vesicles. To demonstrate that the interpretation is correct the SEC profiles before and after incubation should be shown together with SDS-PAGE gels of the protein fractions and negative stain EM (or preferably cryo-EM) should be performed on the SEC fractions similar to those used for lipidomics to show that no vesicles are present in these samples. It would also be interesting to see EM images of the samples with mixed vesicles and Ndh-Ncp filaments to demonstrate direct interaction of the filaments with membranes.

Thank you for your thoughtful comments on this experiment. We agree that it is essential to exclude the possibility that the observed lipid signal arises from co-purified membrane vesicles rather than bona fide association with the Ndh–Ncp complex. To address this, we have repeated the membrane incubation and lipidomics experiments and have now included SEC profiles, SDS-PAGE, and negative stain TEM analyses before and after membrane incubation (**Extended Data Figure 4**), along with a schematic of the workflow.

Briefly, purified Ndh–Ncp was incubated with *B. subtilis* membranes, after which membranes were removed by ultracentrifugation. The resulting supernatant was then subjected to Streptactin affinity chromatography. Notably, the majority of the Ndh–Ncp complex did not bind to the resin, likely due to partial proteolysis of Ncp during membrane incubation, and was recovered in the flow-through. This material was subsequently purified by size exclusion chromatography (SEC), and lipidomics analyses were performed exclusively on SEC fractions corresponding to the Ndh–Ncp elution volume.

The small fraction of Ndh–Ncp that remained bound to the Streptactin resin showed longer filaments and a clear association with membrane vesicles by negative stain TEM (**Figure 3k; Extended Data Figure 4g**), consistent with direct membrane interaction. In contrast, the SEC-purified material used for lipidomics contains filamentous Ndh–Ncp assemblies and does not show overt vesicle association, although minor heterogeneous material is present. Importantly, the SEC fractions used for lipidomics match the elution volume of the purified complex, are well separated from later-eluting species, and have reasonably high purity judged by SDS-PAGE (**Extended Data Figure 4a,b**). Negative stain TEM confirms that these fractions are dominated by Ndh–Ncp filaments. While we cannot completely exclude the presence of trace membrane-derived material, the combined SEC separation and EM analysis indicate that the lipidomics measurements are derived primarily from the Ndh–Ncp complex.

We also note that our revised lipidomics analysis is based on the ratio of quinones to bulk membrane lipids. Any residual membrane contamination would be expected to introduce additional lipids with their native quinone content, which would reduce the apparent quinone-to-lipid ratio. As such, any contribution from co-purified membrane material would lead to an underestimation, rather than an overestimation, of quinone enrichment within the Ndh–Ncp complex.

Finally, we observe partial proteolysis of Ncp during membrane incubation, which leads to some complex disassembly (**Extended Data Figure 4**). However, intact complexes are clearly resolved by Negative stain TEM and were specifically selected for lipidomics analysis, ensuring that the measurements reflect defined Ndh–Ncp assemblies.

Reviewer #2 (Remarks to the Author):

Aerobic organisms face restrictions on respiratory capacity due to limited membrane surface area. Kropp and coauthors have discovered a complex and lipid-rich protein structure which has evolved to overcome this limitation in a subset of Bacillota/Firmicutes, by serving as an “extension” of membrane-localized respiratory machinery into the cytosol. They support this model with three key pillars: structural data, in vitro electron transfer, and elutional lipid identification. Their work is elegant and convincing in its design and execution, and the manuscript is exceptionally well-written.

Though we have considerable enthusiasm for the novelty and impact of this work, we have major concerns about the lack of replication and detail regarding the lipidomic experiments, on which the central conclusions of this work as presented rely.

We thank the reviewer for the time spent reviewing our manuscript.

Major

The primary conclusion of this work is that the Ndh-Ncp complex is a "quinone-transporting pseudomembrane" that amplifies electron transfer. This conclusion relies on the presence of both quinones and phospholipids within the protein complex, and its relative enrichment of quinones. As cryo-EM data and the resulting models alone do not sufficiently support this finding, the authors performed lipidomics to positively identify quinone and phospholipid species eluted from the Ndh-Ncp complex using organic solvents. Their lipidomic design is wholly appropriate, and the menaquinone species-switching repurification experiment is a creative and convincing method to show specific quinone capture.

However, as specified in the legend for Figure 3 (line 342), the data shown to support this finding are from one experiment with no replication. This is insufficient for publication; the final paper must include at least two high-quality lipid datasets that show consistent results across time and separate protein preparations. This is necessary for both the initial purified sample data and the findings after Bacillus membrane treatment.

This is an important point, and we have repeated the lipidomics analyses with appropriate replication across all conditions, including purified Ndh–Ncp, Ndh–Ncp following incubation with *B. subtilis* membranes, and *E. coli* and *B. subtilis* membrane controls. Ndh–Ncp samples (pre- and post-incubation) were analysed in triplicate from independent preparations, while membrane controls were analysed in duplicate from independent preparations. These new datasets are presented in the revised manuscript and replace the original single-replicate measurements. All samples were analysed concurrently by mass spectrometry to minimise instrument-specific variation and ensure robust quantitative comparison across conditions. (**Figure 3f-k; Extended Data Figure 4; Supplemental Figure 4**).

The authors also do not sufficiently support their lipid identification. They state in the Methods (line 803) that lipid identities were confirmed by MS/MS, but do not show any of these data except for MK-7 (and its related MK-9 standard) in Supplemental Figure 4e/f. For any lipid identifications that support the major findings of the manuscript, MS/MS spectra should be shown and annotated for diagnostic ions (e.g. the dimethylnapthoquinone ion at m/z 187 in the displayed menaquinone spectra). This includes the identification of MK-7, MK-8, UQ-8, DMK-8, PE, and PG. For phospholipids, spectra for one member of each family would be sufficient (i.e. one PE and one PG species). Formatting for these MS/MS spectra should be modeled on literature examples, e.g. in Reference 72 (Layre et al 2011), and can be confined to the supplement for this manuscript.

Thank you for your excellent point. We have repeated the lipidomics and included standards for MK-7, PE and PG and the MS/MS spectra for these and shown these compared to a representative data set (Ndh-Ncp after membrane incubation) in the supplemental data (**Supplemental Figure 4**). Unfortunately, we were unable to acquire a standard for UQ-8 however the empirical (not simulated) spectra also acquired on a Thermo orbitrap is available through the m/zCloud database and matches the MS/MS spectra from our samples (**Supplemental Figure 4m & n**). We were unable to locate or obtain a DMK-8 standard or authentic MS/MS spectra and so have revised our manuscript to describe this assignment as putative. We have also included the MS/MS spectra and compared it to the very closely structurally related MK-7 (**Supplemental Figure 4k & l**). We note the only significant difference between the two spectra is entirely consistent with the difference in the 1,4-Naphthoquinone moiety between the two lipids. On this basis we feel it provides extremely compelling evidence for the assignment. Example PE and PG spectra are included for completeness and compared against the corresponding isotopically labelled standards found in the equiSPLASH mixture (**Supplemental Figure 4o-r**). In the revised manuscript, we have also compared the MK-7 peak intensity with lipid features specific to *B. subtilis* membranes, likely corresponding to dihexosyldiacylglycerols (Hex_2DGs) and a diacylglycerol (DG) species. We have presented an example MS/MS spectrum of one of these species (**Supplemental Figure 4s**); however, because we do not have standards for these lipids, we have referred to this assignment as putative in the manuscript.

A major piece of supporting evidence for the respiratory role of the Ndh-Ncp complex is its enrichment of quinones relative to their levels in *E. coli* membrane. The authors compare integrated lipid intensities from purified Ndh-Ncp to a “mass balance estimate” for the *E. coli* quinone to phospholipid ratio (line 309). It is not acceptable to use raw lipid intensities for this

purpose since MS molar responses vary greatly between compound classes (even somewhat within classes); estimating molar or mass ratios of different lipids from mass spectral data requires comparison to lipid standards. The authors could do this by acquiring one commercial standard of similar molecular weight for each relevant lipid family (i.e. one MK, one UQ, one PE, and one PG), and in the same mass spectrometer run establishing standard curves and measuring experimental samples. Alternatively, the authors could bypass the need for standards by measuring relative lipid ion intensities (quinone vs phopholipids) in solvent-extracted membranes from the same E. coli strain used to isolate the complexes, and with this as a reference measure quinone enrichment in the isolated complexes measured under identical conditions (same day, same instrument).

We thank the reviewer for this important point regarding variation in ionisation efficiency across lipid classes in MS-based analyses. We agree that direct comparison of raw lipid intensities is not appropriate for estimating molar ratios across chemically distinct species. In response, we have revised our lipidomics analysis using two complementary approaches, depending on the availability of appropriate standards.

For the initially purified Ndh–Ncp complex and *E. coli* membrane samples, we performed quantitative analysis using isotopically labelled internal standards. Specifically, each sample was spiked with defined quantities of deuterated standards representing the major lipid classes (MK-7-d7, PE 33:1 (d7), and PG 33:1 (d7)), and all samples were analysed concurrently on the same instrument. The concentration of each lipid species was estimated by comparing its peak area to that of a chemically matched internal standard. This approach accounts for ionisation efficiency differences and matrix effects and allows estimation of molar quantities and quinone-to-phospholipid ratios within each sample. We note that isotopically labelled standards for ubiquinone (UQ) and demethylmenaquinone (DMK) were not available. For these species, we used MK-7-d7 as a proxy standard, which is justified given their closely related chemical structures and ionisation properties. While this introduces some uncertainty in absolute quantitation, it does not affect the relative comparison between samples analysed under identical conditions.

For the *B. subtilis* membrane incubation experiments, appropriate standards were not available for several lipid classes (e.g. Hex_2DG and DG species). In this case, we adopted a relative quantification strategy by comparing the ratio of MK-7 to *B. subtilis*-specific lipid features. These ratios were then compared between *B. subtilis* membrane samples and post-incubation Ndh–Ncp samples analysed under identical conditions. Because these comparisons are performed

within the same analytical run and between chemically similar species, this approach is relatively robust to differences in ionisation efficiency and is consistent with the alternative strategy suggested by the reviewer. However, individual lipid intensities in samples may be influenced by matrix effects, and we are careful to point out this limitation in the manuscript (**Lines 380-382**). Importantly, this analysis shows that MK-7 is enriched in the Ndh–Ncp complex following incubation with *B. subtilis* membranes.

Together, these revised analyses address the reviewer's concern by replacing raw intensity comparisons with either internal standard-based quantification (where possible) or internally controlled relative comparisons within matched samples.

Minor

The purity of the isolated Ndh-Ncp complex is key for interpreting the lipidomics findings, but the only evidence shown of preparation purity is an SDS-PAGE gel in a small inset for Figure S1d. This gel should be expanded to a viewable size and shown in full (not cropped), along with any other relevant supporting data for protein complex purity.

We agree with the reviewer's assessment. We have enlarged the SDS-PAGE gel of the purified Ndh-Ncp complex, now **Extended Data Figure 1d**. We have also added supporting data for the membrane incubation experiment in **Extended Data Figure 4,** including SDS-PAGE of the Ndh-Ncp complex before and after membrane incubation and re-isolation.

Many figure panels are extremely small in this version of the manuscript, with text and details not discernible on the printed page. The most severe (but not only) examples are Figures 1b, 4d, 5a, S1d/f/g/h, and S4e/f. All text and details in all figures must be understandable when printed on standard size paper, with the potential exception of the phylogenetic trees specifically in the supplement.

We have revised the manuscript figures to improve readability.

DMK-8 is only defined in a figure legend and not the main text

Amended.

Lipidomic data in the listed repository (line 808) are inaccessible to reviewers

When the Supplemental Data 1 spreadsheet is opened in Excel, it asks the user to sign in with University of Melbourne credentials to update links. The data appear to be intact when this prompt is ignored, but this should be fixed.

These supplemental data sheets have been fixed.

Some details are missing from the lipidomics methods and data. Sample injection volume is stated, but not the total resuspension volume from which this injection was withdrawn. Solvent compositions are incomplete (A = 40% isopropanol, 60% ???). It is unclear whether targeted peak areas were taken from mzMine or a different software, and which peak areas were used in Figure 3f/g (the ones in Supplemental Data 1, or others?).

Amended.

Additional discussion would be helpful regarding why so much ubiquinone signal was observed in Figure 3f, and yet ubiquinone neither fits the molecular model nor is active by kinetics or cyclic voltammetry.

This is an important point, and we have clarified this in the revised manuscript (**L328-329**) "UQ-8 was the most abundant quinone associated with the complex (**Figure 3f**), however, as *B. subtilis* primarily utilises MK-7, this likely reflects the *E. coli* expression system rather than substrate preference". The lipidomics data show that substantial UQ-8 is present both associated with Ndh-Ncp and in *E. coli* membranes, which is consistent with its role as the major respiratory quinone in *E. coli* during aerobic growth. We interpret the strong UQ-8 signal as reflecting its abundance in *E. coli*, rather than a true substrate preference of Ndh. We detect menaquinone species (including MK-8) in both *E. coli* membranes and purified Ndh-Ncp (**Figure 3f,g; Supplemental Data 2**). This suggests that, despite their lower abundance relative to UQ-8 under aerobic conditions, menaquinone is present at sufficient levels to be extracted by the Ndh–Ncp complex. We propose that Ncp itself is not highly selective for the quinone species it internalises, as both ubiquinone and menaquinone share hydrophobic isoprenoid tails and broadly similar quinone head groups. Instead, the observed substrate specificity and MK binding preference are dictated by the associated Ndh enzyme. *B. subtilis* exclusively utilises menaquinone (predominantly MK-7), consistent with the preference of Ndh-Ncp for naphthoquinone substrates.

In line 446, “gram-negative” should be “gram-positive”

Amended.

Reviewer #3 (Remarks to the Author):

The work by Kropp et al describes the characterization of complex composed by the type II NADH dehydrogenase, Ndh-2 and the quinone-transporting protein, Ncp. The genes coding for these proteins were cloned as one continuous fragment, which also codified for an N-terminal twin strep II tag. The complex was produced in E. coli and purified from the cytoplasm. The obtained cryo-EM structure of the Ndh-2 and Ncp complex is amazing. However, evidence for physiological relevance of the assembling of those complexes into filaments is missing.

We thank the reviewer for the time spent assessing our manuscript.

Major observations:

1- Physiologic relevance of the observed filaments.

The cryo-EM structures show the Ndh-2 and Ncp complexes assembled into filaments containing up to 13 units. The work does not provide evidence for physiological relevance of the existence of such filaments. It is well known and described in literature that many proteins or proteins complexes aggregate forming filaments under certain conditions. The studied complexes were produced in E. coli and obtained from the cytosolic fraction, which is intriguing for a complex that uses a lipid soluble substrate, a quinone. Such a large filaments should be observed in the cell (B. subtilis) by Cryo-electron tomography.

We thank the reviewer for this important point regarding the physiological relevance of Ndh–Ncp filament formation. We agree that establishing the presence of such assemblies in vivo would be highly valuable.

Visualisation of protein filaments of this size within Gram-positive cells remains technically challenging using Cryo-electron tomography and would require specialised approaches, such as cryo-focused ion beam (cryo-FIB) milling and advanced sample preparation methods, in

combination with subtomogram averaging and genetic perturbations (e.g. fluorescent labelling or knockout strains) to enable unambiguous identification. Even in favourable cases, distinguishing specific protein assemblies from the dense cytosolic background is non-trivial and generally requires a dedicated experimental workflow, meaning a successful outcome would be highly uncertain.

Given these considerations, we believe that definitive in situ visualisation of Ndh–Ncp filaments would require a substantial additional programme of work and is beyond the scope of the current study. Importantly, our conclusions regarding filament formation are now supported by multiple independent lines of evidence, including high-resolution cryo-EM structures, biochemical characterisation, mutational analysis of filament interfaces, and observation of filamentous assemblies formed from endogenously isolated Ndh-Ncp complexes in *B. subtilis* (see below). Together, these data support the physiological relevance of filament formation, even in the absence of direct in-cell visualisation.

The main problem of the work is the physiological occurrence of the filaments. How are filaments excluded from being an artifact from heterologous protein production or/and purification procedures? Or larger complexes are better detected in cryo-EM data processing. Did the authors perform an analytic SEC to determine the complex molecular mass or a native electrophoresis? These are important characterisations missing in the manuscript.

Thank you for your comment and suggestions. We incorporated these suggestions, taking several complementary approaches to demonstrate that Ndh–Ncp filament formation is specific, stable, and physiologically relevant.

First, we examined Ndh–Ncp in *B. subtilis* lysates using native PAGE coupled to NADH activity staining (**Extended Data Figure 3a**). In wild-type cells, Ndh–Ncp activity appears as a broad band consistent with higher-order assemblies larger than a single complex. No activity at this size range is observed in *Δndh* or *Δncp* strains, confirming that the observed assemblies correspond specifically to the Ndh–Ncp complex.

Second, we now provide direct evidence that Ndh–Ncp filaments form under native expression conditions in *B. subtilis*. Using activity-guided fractionation of cell lysates, which demonstrates that the endogenous complex exhibits similar biochemical behaviour to the recombinant protein, we isolated the endogenous Ndh-Ncp complex and observed filamentous assemblies by negative stain TEM. These filaments match the dimensions and periodicity of the recombinant

complex, and mass spectrometry indicates Ndh and Ncp are highly enriched in the sample (**Extended Data Figure 3b-g, Supplemental Data S1**). This demonstrates that filament formation is not an artefact of heterologous expression or purification in *E. coli* but occurs in protein produced at normal levels in the native host. Notably, this native isolation strategy avoids overexpression and extreme concentration of the protein, reducing the likelihood of artefactual filament formation.

Third, we show that filament formation is mediated by a defined and conserved protein–protein interface. The Ncp–Ncp interaction forms a complementary charged and hydrophobic "head-to-tail" interface that generates a continuous conduit along the filament. Sequence analysis across Bacillota reveals that residues comprising this interface are highly conserved (**Extended Data Figure 2a,b**), indicating that filament formation is evolutionarily maintained and supports the formation of a continuous hydrophobic conduit for quinone transfer between Ndh–Ncp complexes.

Fourth, we demonstrate that the recombinant Ndh–Ncp complex does not form non-specific aggregates. Fractions across the SEC peak were analysed by native PAGE and mass photometry (**Supplemental Figure 1**), revealing a distribution of assemblies ranging from ~350 kDa to ~5 MDa. These data indicate a dynamic equilibrium of discrete filament lengths rather than irreversible aggregation. This behaviour is inconsistent with non-specific aggregation and instead supports regulated assembly.

Taken together, the structural, biochemical, and native purification data provide strong evidence that Ndh–Ncp filament formation is a specific and physiologically relevant property of this complex, rather than an artefact of recombinant expression or purification.

The hypothesis put forward by the authors that the filaments interact with the membrane transiently is difficult to understand since several quinone/quinol molecules have to have time to interchange to/from the hydrophobic interior of the filament from/to the membrane.

We thank the reviewer for this important point regarding the dynamics of membrane interaction and quinone exchange. In the revised manuscript, we provide direct evidence that Ndh–Ncp interacts with membranes (**Figure 3k**), and similar behaviour has been reported for other complexes containing Ncp-like proteins, including Huc from *Mycobacterium smegmatis* and ForCE from *Bacillus subtilis*. Such interactions are necessary for quinone extraction and delivery, as quinones are lipophilic and reside within the membrane.

At the same time, Ndh–Ncp can be purified from the soluble fraction, and both Huc and ForCE are similarly isolated as soluble complexes from their native hosts. This indicates that these assemblies are not stably membrane-integrated but instead associate with membranes in a dynamic manner. The interaction interface in these systems is largely electrostatic and lacks features characteristic of integral membrane proteins, further supporting a reversible mode of association.

We note that quinone exchange does not appear to require prolonged or stable membrane binding, with transient or repeated interactions sufficient to enable efficient transfer into the Ncp conduit. Consistent with this, the Ndh–Ncp complex purified from *E. coli* contains quinones derived from the expression host, despite not co-purifying with significant quantities of lipid vesicles, demonstrating that membrane interaction occurs on a timescale compatible with quinone uptake.

To better reflect this, we have revised the manuscript to avoid over-specifying the interaction as strictly "transient" and instead describe it as dynamic and potentially influenced by host membrane composition (**Lines 241-242**). We have also clarified that quinone exchange likely occurs during brief or repeated membrane interactions, rather than requiring stable membrane association.

2- The putative existence of the filaments does not contribute to expand bioenergetics. Even if filaments were physiologically relevant, these would not substitute for additional membrane surface area in terms of energy conservation as the case of mitochondrial inner membrane invagination. The filaments would contribute possibly to a higher supply of the lipid soluble substrate, the quinone. This is because Ndh-2 is not involved in energy transduction and no transmembrane potential would be established in the filaments (it is established in the invagination of the mitochondrial inner membrane). The authors should clarify these different concepts.

We thank the reviewer for this important clarification. The reviewer is correct that the Ndh–Ncp filaments do not contribute directly to transmembrane potential generation and, in this sense, are not analogous to mitochondrial inner membrane invaginations, which increase the surface area available for proton-pumping complexes. Our interpretation is instead that the filaments enhance redox flux through two related mechanisms. First, the extended scaffold increases the local concentration of Ndh and its access to membrane-derived quinones. Second, the internal cavity of the filament can accommodate multiple quinone molecules, potentially acting as a

reservoir that expands the effective cellular quinone pool. In *B. subtilis*, which lacks Complex I, Ndh performs NADH oxidation and quinone reduction without contributing to proton translocation; the resulting quinol is instead oxidised by terminal oxidases that generate the proton motive force. The Ndh–Ncp filament can therefore be viewed as enhancing electron supply to the quinone pool, supporting downstream energy conservation. Importantly, because this activity occurs within a filamentous assembly that occupies minimal membrane surface area, a greater proportion of the membrane may remain available for terminal oxidases and other energy-conserving complexes.

3- The existence of filaments would make quinol oxidation a limiting factor. The existence of filaments would allow the accumulation of quinol, the reaction product and quinols may react with O2 producing radical oxygen species. Thus, it is difficult to understand the reason for a cell to carry such a potential producer of radical oxygen species.

We agree that excessive reduction of the quinone pool could, in principle, increase the risk of reactive oxygen species formation if quinol oxidation were limiting. However, we do not think this concern is specific to the filamentous architecture of Ndh–Ncp but rather reflects a general requirement for balance between quinone reduction and quinol oxidation in any respiratory chain. In our view, the Ndh–Ncp filament is not expected to drive pathological quinol accumulation, but instead to support efficient electron flux into the quinone pool. In *B. subtilis*, Ndh–Ncp is a major respiratory input enzyme, and deletion of either Ndh or Ncp impairs growth, indicating that this system makes a positive contribution to respiratory metabolism rather than creating a detrimental redox imbalance. More broadly, our evolutionary analysis suggests that Ndh–Ncp arose from membrane-associated NDH-2 enzymes, supporting the idea that filament formation is an adaptation to sustain quinone reduction under the physiological demands of this organism. Importantly, our electrochemical data indicate that quinone/quinol exchange between the Ndh–Ncp complex and the membrane is dynamic, rather than consistent with sequestration of quinol within the filament. We therefore favour a model in which the filament facilitates rapid quinone/quinol flux, analogous to diffusion within the membrane phase, rather than one in which reduced quinol accumulates locally to harmful levels.

4- Menadione as preferential quinone substrate

Menadione (MDA) is water soluble and the quinone binding site of Ndh-2 is facing the hydrophobic tunnel composed of Ncp subunits. In this case how does MDA interact with Ndh-2?

Does it interact at the quinone binding site? If not, what is the relevance of the data obtained with MDA.

Thank you for this important point. Menadione (MDA) is more water-soluble than long-chain quinones such as menaquinone-7, but with an octanol-water partition coefficient log(P) of 2.2 (https://pubchem.ncbi.nlm.nih.gov/compound/menadione), it remains sufficiently hydrophobic to partition into lipid environments and access hydrophobic regions of the protein. In this context, we consider menadione to act as a soluble analogue of the quinone head group rather than a fully membrane-embedded substrate. We propose that menadione can access the Ndh quinone-binding site via the Ncp channel, consistent with its amphiphilic character and small size. Importantly, our use of menadione is intended to probe substrate preference at the level of the quinone head group. The observed preference for menadione over ubiquinone is therefore most readily explained by the compatibility of its 1,4-naphthoquinone head group with the Ndh active site, consistent with the native use of menaquinone by *B. subtilis* Ndh. This interpretation is supported by our structural and functional data. The cryo-EM density within the channel is consistent with a two-ring quinone head group, and both kinetic and electrochemical measurements indicate a preference for naphthoquinone-type substrates over ubiquinone, despite the higher abundance of ubiquinone in the expression system. We therefore use menadione as a tractable proxy to isolate head-group recognition by Ndh, while the full-length isoprenyl tail in native quinones is expected to primarily influence membrane localisation and partitioning rather than catalytic specificity.

Moreover, in order to assess quinone specificity the authors compared the catalytic properties of the reaction using MDA, the redox head group of menaquinone, which has no hydrophobic tail and is thus water soluble, with the reaction using decylubiquinone, a benzoquinone with a tail containing 10 hydrocarbon groups and thus a very hydrophobic molecule. Please comment

Thank you for this point. To assess quinone specificity, we compared the catalytic properties of Ndh–Ncp using menadione (MDA), which represents the redox-active head group of menaquinone, and decylubiquinone, a benzoquinone analogue with a hydrophobic tail.

Our combined kinetic, electrochemical, and structural data indicate that the *B. subtilis* Ndh quinone-binding site exhibits a clear preference for 1,4-naphthoquinone head groups, as found in menaquinone and MDA. The cryo-EM density within the binding region is consistent with a two-ring quinone head group, and both kinetic and electrochemical measurements show enhanced activity with naphthoquinone-type substrates relative to benzoquinones. We therefore interpret

the higher apparent activity with MDA compared to decylubiquinone as primarily reflecting favourable interactions between the naphthoquinone head group and residues within the Ndh active site. While both substrates can access the enzyme, their differing physicochemical properties (including solubility and membrane partitioning) may also influence apparent kinetics, and we do not exclude a contribution from these factors. Together, these data support a model in which head-group chemistry is the dominant determinant of substrate recognition by Ndh, while the isoprenyl tail primarily influences membrane association and substrate availability.

5- Structure with substrates

It is not clear how the sample(s) to obtain substrate-bound structure was(were) prepared. On line 244 it is briefly referred "we repeated the cryo-EM, supplementing Ndh-Ncp with NADH immediately before freezing." (no mention to this in MM section) and then on the following line the authors mention "We resolved this complex at a global resolution of 2.52 A, showing clear density for the FAD group, NADH and quinone, at their structurally conserved binding sites. Where did the quinone come from? Was it added or do the authors hypothesised it was in the tunnel? (based on the lipidomics data the authors mentioned (line 314) "that it is likely that quinones occupy both canonical Ndh quinone binding sites and the central Ndh-Ncp cavity." Although not quinone is observed at its binding site in the structure of the "as prepared" complex).

For the substrate-bound structure, NADH was added to the purified Ndh–Ncp complex at a final concentration of 5 μM, 10 minutes prior to grid freezing. This information has now been included in the Methods (**L769-770**): "For the NADH-bound structure, Ndh–Ncp (6 mg ml^{-1}) was incubated with 5 μM NADH for 10 min prior to freezing." No exogenous quinone was added at any stage. Consistent with our lipidomics and electrochemical data, which indicate that quinones are retained within the Ndh–Ncp assembly, we conclude that the quinone observed in the structure co-purified with the complex during expression in *E. coli*.

In the initial (apo) structure, we did not observe clear density for quinone in the Ndh binding site. In contrast, in the NADH-supplemented dataset, we observe well-defined density at this site consistent with a bound quinone. This difference may reflect increased occupancy under turnover-relevant conditions, where quinone binding is influenced by local conformational or redox state changes.

We have added text to clarify the absence of exogenous quinone and the likely origin of the bound quinone (**L296-297**): "No exogenous quinone was added to the Ndh-Ncp complex, meaning the

observed quinone was likely extracted during *E. coli* expression and retained through protein purification."

If quinone was in the tunnel it would be available for reduction by NADH and thus no CTC would be observed. How do the authors conciliate the observation of a CTC upon addition of NADH to the complex when quinone is present? Was the amount of NADH added (how much was it?) higher than that of the quinone? Can the presence of quinone be detected in the UV-Vis spectra? The amount of quinone present may possibly be quantified by the amount of NADH added.

We thank the reviewer for this insightful comment. Below, we address each point in turn.

Yes. In the spectroscopic titration, we added 0–100 μM NADH to 85 μM Ndh-Ncp. Our lipidomics data estimate total menaquinone content (MK + DMK) at ~188 pmoles from 48.6 μg of Ndh-Ncp (MW = 230,140 Da; 211 pmoles of complex), yielding a molar ratio of approximately 0.89 quinone molecules per complex. At 85 μM complex, this corresponds to roughly 75 μM quinone equivalents — meaning that NADH concentrations above ~75 μM exceed the estimated quinone content. CTC formation and FAD reduction both saturate at ~60 μM NADH, a concentration at which NADH approaches parity with available quinone. This is consistent with NADH gaining access to active sites not occupied by quinone.

Both menaquinone and ubiquinone absorb exclusively in the far-UV region, menaquinone at ~248 nm and ~270 nm (Cao, Du et al. 2020), and ubiquinone (oxidised form) at ~270–275 nm (Schopfer, Riobo et al. 2000). At these wavelengths, absorbance is dominated by aromatic amino acid residues and the protein backbone, making it impractical to resolve or quantify quinone above background. UV-Vis spectroscopy was therefore not a viable approach for quinone quantification in this system; we relied instead on the lipidomics approach described above.

We agree that the observation of CTC upon addition of NADH in the presence of quinone is curious, and we acknowledge that the available data do not permit a fully resolved stoichiometric model. We offer the following considerations.

Our lipidomics data indicate that quinone is present at substoichiometric levels relative to the total number of Ndh active sites. Based on 211 pmoles of complex (48.6 μg of Ndh-Ncp @ MW = 230,140 Da) used for lipid extraction and 4 Ndh active sites per complex, we estimate ~844 pmoles of active sites in total, against only ~188 pmoles of menaquinone, implying that at most ~22% of sites are quinone-occupied, leaving the remainder available to form a CTC upon NADH addition. This substoichiometry provides a straightforward basis for the observed CTC signal.

However, the spectroscopic titration independently suggests that only a subset of theoretical active sites respond to NADH: FAD reduction, and the CTC signal both saturate at ~60 μM NADH against a theoretical active site concentration of 340 μM (85 μM complex × 4 sites), corresponding to approximately 18% of theoretical capacity. This fraction is broadly consistent with the quinone occupancy estimated from lipidomics, which does not readily explain the formation of the CTC.

We acknowledge, however, that this picture is not straightforwardly reconciled with our cryo-EM quinone occupancy (see below), which shows relatively high occupancy of MK-8 at the quinone-binding site. This high structural occupancy is inconsistent with the ~22% bulk occupancy inferred from lipidomics. The most likely explanation for this discrepancy is that the two techniques sample different subpopulations of the complex: cryo-EM particle selection favours intact, well-ordered complexes in which the quinone-binding site is occupied, whereas lipidomics reflects the total lipid content of the preparation, which may be biased downward by losses during extraction or by the inclusion of partially disassembled or quinone-depleted complexes that contribute protein but not lipid signal. The cryo-EM occupancy, therefore, likely represents an upper bound on site occupancy, and the lipidomic estimate a lower bound, with the true occupancy lying somewhere between. Given these uncertainties, we are cautious of over-interpreting either figure or presenting the stoichiometric calculation in the main manuscript.

What is the occupancy of NAD+/NADH and of quinone in the cryo-EM structures?

Thank you for this question. In the refined cryo-EM model, both NADH and quinone were modelled at full occupancy (occupancy = 1), based on the presence of continuous and reasonably well-defined density at their respective binding sites. The refined B-factors for these ligands are modestly higher than those of the surrounding protein (approximately 125 for NADH and 112 for quinone, compared to ~60–100 for the protein), indicating increased mobility or local heterogeneity, but not to an extent that warranted modelling partial occupancy. We note that cryo-EM density represents an average over the particle population, and therefore the assigned occupancies reflect the best fit to the observed density rather than a direct measurement of absolute biochemical occupancy.

6- MD simulations suggest that the Ndh-Ncp complex tilts so the opening of the Ncp tube penetrates the hydrophobic region of the lipid bilayer. What is the relevance of this tilting? Does

the complex have to tilt in order to exchange quinone/quinol with the membrane? How could it be possible in the case of a filament? And only with a transient interaction between the filament and the membrane?

The tilting observed in the MD simulations may be relevant in that it could improve access of the Ncp tube opening to the hydrophobic core of the membrane, thereby facilitating quinone/quinol exchange. However, we are cautious of overinterpreting the physiological significance of this motion from the current data alone. At present, we cannot conclude whether tilting is required for quinone exchange. In our negative stain images, the Ndh–Ncp filament appears to associate with membrane vesicles in a largely upright orientation, although tilting appears to occur in some instances (**Figure 3k**). We note that this is also a simplified experimental system. We therefore interpret the MD result as identifying one possible membrane-engaged state, rather than establishing a required mechanism. With respect to the filament, we do not currently have sufficient mechanistic data to determine whether filament formation substantially alters the geometry of membrane interaction or the need for tilting. At this stage, our data support the conclusion that the interaction must be sufficiently dynamic to allow quinone transfer between the membrane and the tube, but the structural basis of this exchange remains to be firmly established.

Minor observations:

1- Please refer to type-II NADH dehydrogenase or alternative NADH dehydrogenase always as Ndh-2 and not only Ndh, which refers to NADH dehydrogenase homologous to respiratory complex I.

We appreciate the reviewer's comment but respectfully maintain our use of "Ndh" throughout the manuscript. Ndh is the established name of this specific *B. subtilis* protein, as documented in SubtiWiki (https://subtiwiki.uni-goettingen.de/v5/gene/1292), where the gene is designated ndh and the protein Ndh. In model organisms, referring to proteins by their canonical gene/protein names is considered good practice and promotes clarity and consistency with the broader literature.

Importantly, "Ndh-2" is a functional descriptor referring to the type-II NADH dehydrogenase family more broadly, whereas Ndh refers specifically to this *B. subtilis* protein. In addition, *B. subtilis* encodes a second type-II NADH dehydrogenase, YumB, which functions in spores. Using "Ndh" unambiguously distinguishes the protein studied here from YumB, whereas the generic

"Ndh-2" designation would not. We therefore believe that "Ndh" is the most precise and appropriate name, and that the assembly is best described as the Ndh–Ncp filament.

2- What is the protein concentration used in UV-Vis spectroscopy? What is the protein NADH stoichiometry?

The concentration of Ndh-Ncp used in UV-Vis spectroscopy was 85 µM. There was 0-100 µM of NADH added, so the stoichiometries are (NADH/Ndh-Ncp): 0, 0.2, 0.4, 0.6, 0.8, ~1. The materials and methods (**Lines 742-743**) have been amended with the protein concentration for this assay.

3- The UV-Vis spectrum of the NADH-reduced complex presents a band/shoulder at ~420 nm. What is the origin of this band/shoulder?

A shoulder at ~420 nm is characteristic of very small amounts of haem contamination in the preparation. As the flavin has a much smaller extinction coefficient compared to haems, it is very demanding to fully remove this signal without many rounds of SEC, which can then lead to removal of the flavin. The shoulder is found in other NDH-2 enzymes in the literature (Blaza, Bridges et al. 2017), but it is much less obvious here due to the preparation being cleaner.

4- Clade D – Extracellular electron transfer may be misleading by indicating that those enzymes may be facing the outside of the cell.

We appreciate the reviewer's point. Previous reports have indicated that these Clade D enzymes form part of an extracellular electron transfer system. This system functions by oxidising NADH from the intracellular space where the NDH-2 enzyme domain is located and transmitting the electrons either to quinones in the membrane or directly to substrates in the extracellular space. We have modified the text (**L534**) to better reflect this: "Interestingly, clade D represents NDH-2 enzymes that associate with the membrane through transmembrane helices, with previous work indicating this clade forms a component of an extracellular electron transport system..."

References:

Blaza, J. N., H. R. Bridges, D. Aragão, E. A. Dunn, A. Heikal, G. M. Cook, Y. Nakatani and J. Hirst (2017). "The mechanism of catalysis by type-II NADH:quinone oxidoreductases." Scientific Reports **7**(1): 40165.

Cao, S., X. Du, P. Li, G. Yuan, S. Chen, W. Chen, X. Song and B. Kuang (2020). "A chemical screening method for menaquinone-producing strains based on HPLC-UV technology." Journal of Microbiological Methods **172**: 105907.

Schopfer, F., N. Riobo, M. C. Carreras, B. Alvarez, R. Radi, A. Boveris, E. Cadenas and J. J. Poderoso (2000). "Oxidation of ubiquinol by peroxynitrite: implications for protection of mitochondria against nitrosative damage." Biochemical Journal **349**(1): 35-42.

Reviewer #1 (Remarks to the Author):

The authors have successfully responded to my comments and concerns. I congratulate the authors with a beautiful piece of work and significant contribution to the field.

Thank you for your review and the kind appraisal of our work.

Reviewer #2 (Remarks to the Author):

Comments for the authors

Kropp and coauthors have meaningfully improved their already impressive manuscript with additional experiments and rewriting. These changes constitute a more-than-satisfactory response to both our major and minor concerns. This thorough response also extended to the concerns of other reviewers, many of which we agree with and believe were sufficiently addressed during revision.

We have a few very minor points that could help future reader understanding. The revised manuscript combines Extended Data Figures with Supplemental Figures, which is confusing and challenging to navigate. Extended Data Fig. 4d specifies "DGDG" on its y-axis, an undefined acronym, while the figure legend references Hex2DG. Supplemental Figure 6 is mostly too low-resolution to be interpretable, even when zoomed on a computer screen.

Thank you for your review and the kind appraisal of our work. We have amended Extended Data Figure 4g and relabelled the axis with Hex_2DG and added the description of this into the figure legend. The resolution of Supplemental Figure 6 appears high-resolution in our exported manuscript, so could be a compression issue. We will ensure our submitted figure is high-resolution.

The additional work in this revision has produced a manuscript which we were unabashedly enthusiastic to read, and which we believe will significanty move the field forward with respect to understanding respiration in gram positive bacteria.

Reviewer #3 (Remarks to the Author):

The authors addressed all the points raised by the reviewers, completing their answers with further experiments, which make the work much more robust. Nevertheless, the manuscript would still benefit from the clarification of some points.

Major observations:
1- Physiologic relevance of the observed filaments.
I understand the authors' arguments and encourage them to introduce these arguments in the Discussion section.

We thank the reviewer for appreciating the complexities of verifying the filaments in *B. subtilis* using cryo-ET. We have not added our rationale as to why investigating filament formation in cells using imaging techniques and cryo-ET to the discussion, primarily due to manuscript length constraints. We also acknowledge that it is widely known and discussed in the cryo-ET literature that Gram-positive microbial substructures are difficult to investigate using cryo-ET and require complex and lengthy preparation procedures for any visualisation. Thus, we feel any further discussion of this in our manuscript is unnecessary, given we showed through other means that this complex is filamentous in cells.

2- The putative existence of the filaments does not contribute to expand bioenergetics.
I understand and agree with the authors' arguments that "the filaments enhance redox flux". In fact, in their answer to my concern they mention, "The reviewer is correct that the Ndh–Ncp filaments do not contribute directly to transmembrane potential generation and, in this sense, are not analogous to mitochondrial inner membrane invaginations, which increase the surface area available for proton-pumping complexes." In this way, the introduction section from lines 50 to 64 should be rewritten accordingly. As it is, it can be misleading.

We agree the filaments do not contribute directly to proton pumping and are not analogous to mitochondrial cristae in that respect. However, the reviewer's concern that this makes the filaments irrelevant to bioenergetics overlooks that quinone reduction and oxidation are themselves proton-translocating steps. Ndh takes up two protons from the cytoplasm per quinone reduced; quinol reoxidation at the terminal oxidase releases protons on the external side. This chemiosmotic redox loop generates membrane potential independent of any pumping activity by the oxidase itself. Expanding Ndh capacity therefore increases flux through this loop and expands respiratory capacity, without requiring the filaments to pump protons directly. Lines 50-64 already avoid claiming Ndh-Ncp is a proton pump or a direct analogue of cristae; they argue only that Gram-positive bacteria face a constraint on electron-transfer capacity due to reduced membrane surface area, and that Ndh-Ncp addresses this constraint.

3- Structure with substrates
It is not clear how much the occupancy of NADH binding sites is. The authors mentioned that "For the NADH-bound structure, Ndh–Ncp (6 mg ml^{-1}) was incubated

with 5 μM NADH for 10 min prior to freezing.”. Considering MW=230140 Da, it means that Ndh–Ncp concentration is ~26.07 μM, more than 5 times that of NADH (protein/NADH ration is 1:0.25).

Thank you for this comment and we appreciate that you have pointed out an error in our manuscript. The amount of NADH added was written incorrectly in the manuscript, rather than 5 uM, we added **5x the molar concentration** of NADH (~130 uM NADH). This then aligns with our statement that the occupancy of NADH is 1 in our cryo-EM structure.

Is quinone only observed when NADH is present? The authors mentioned an occupancy of 1 both for NADH and quinone, which is not possible considering the amount of NADH added. How do the authors conciliate these observations? In fact, 25% agrees quite well with the ~22% bulk occupancy inferred from lipidomics. Please discuss.

While lipidomics indicates that menaquinone is present in the NADH-free, as-purified Ndh-Ncp complex, we only observe density for quinone in the structure of Ndh-Ncp bound to NADH. As noted in our response above, the NADH concentration used for this structure was in 5x molar excess over protein, not 0.25x as stated in the manuscript; at this concentration, near-full NADH occupancy is expected and not itself remarkable. We modelled both NADH and menaquinone at an occupancy of 1. Per our standard practice, we fix ligand occupancy at 1 during refinement and instead express uncertainty in occupancy/flexibility through the B-factor, which is the more sensitive indicator when working near the noise floor; both ligands refined with B-factors only modestly elevated relative to the surrounding protein, consistent with high occupancy.

We note that the reviewer's calculated agreement between the (erroneously reported) 25% NADH occupancy and the ~22% bulk quinone occupancy from lipidomics does not hold once the NADH concentration is corrected and should not be read as evidence of coupled occupancy between the two ligands. The question of quinone occupancy on its own terms, however, is worth addressing directly. The ~22% figure from lipidomics is lower than we would expect given the quality of the cryo-EM density, but we are cautious about over-interpreting it as the molar concentration of quinone was estimated using standards on a separate preparation from the one used for imaging. We therefore favour the cryo-EM occupancy as the more reliable estimate for this specific complex, while acknowledging that reconciling it fully with the bulk lipidomics figure will require further work.

A discussion on the “curious” establishment of a CTC in the presence of quinone should also be included in the manuscript.

We agree this is an interesting observation. The CTC we observe reflects charge-transfer character between reduced FAD and NAD+ following hydride transfer from NADH, and its persistence in the presence of quinone implies that intramolecular electron transfer from FAD to the quinone-binding site is not fast enough to fully deplete the charge-transfer species on the timescale of our measurement, or that the two processes are in a steady-state equilibrium rather than a strict sequential hand-off. Distinguishing between these possibilities (kinetic competition between intramolecular electron transfer and quinone reduction, versus a distinct conformational or binding-site gating step) would require pre-steady-state kinetics, which are beyond the scope of the current structural and lipidomic dataset. We think this is a valuable direction for future mechanistic work on this complex, but consider it premature to speculate on the underlying basis in the manuscript itself, and prefer to keep the discussion focused on the structural and compositional findings presented here.

4- MD simulations
I agree with the authors being cautious about the physiological significance of the tilting suggested by the MD simulations. But then what is the relevance of the MD simulations? Why weren't B. subtilis native lipids considered?

Thank you for understanding our cautious approach to interpreting the MD simulations. The purpose of the MD simulations is to serve as an alternate technique to understand lipid engagement with the Ndh-Ncp complex, complementing our cryo-EM and lipidomics data. We used *E. coli* lipids given this is what is in our sample from recombinant purifications and therefore in our cryo-EM and lipidomics data. Importantly, our MD simulations were guided by these data to ensure accuracy of the system and that our data aligned. Without purifying Ndh-Ncp from *B. subtilis* and quantitating the lipids associated with the complex, we cannot accurately add the correct lipids for MD simulations in the correct ratios. This could be expanded upon further with *B. subtilis* lipids in future studies, but for our purposes is outside of the scope of this paper.

Minor observations:
1- Mutations: Were complementation assays or whole genome sequencing performed?

Complementation and whole-genome sequencing were not performed for this study. We did, however, confirm the mutations by sequencing the mutant loci directly, verifying that these are bona fide knockouts. Furthermore, the growth phenotype we observe closely matches that previously reported for Ndh knockouts in the literature(Gyan, Shiohira et al. 2006), which supports that the phenotype is attributable to loss of Ndh function rather than a secondary genomic effect.

2- Line 66, Only an integral membrane subunit is needed for these enzymes, the presence of prosthetic groups in these subunits is not always a requirement, such as in the case of complex I, for example. Thus, “containing haem or iron-sulphur clusters“ should be removed.

We have amended the text to "often containing heme or iron-sulfur clusters" to reflect that not all integral membrane subunits carry such cofactors. We note, however, that Complex I is not a clean example of this: its membrane-embedded arm performs no redox chemistry itself and functions as a proton pump structurally related to Mrp-type antiporters, driven by long-range conformational coupling to the cofactor-containing peripheral arm rather than by any prosthetic group within the membrane domain itself. In this sense Complex I's membrane subunits are not "electron-transport machinery lacking cofactors" so much as a distinct proton-translocation module altogether. We think this supports retaining a hedged description rather than removing the reference to cofactors, since several of the other systems cited (e.g., cytochrome-containing and Rieske-type complexes in thylakoids and related systems) do rely on membrane-embedded cofactors for electron transfer.

3- Line 75, What are “high-energy electrons”? Please remove high-energy.

We have amended the text to describe these as "low-potential electrons," which more precisely reflects that the driving force for electron transfer arises from the reduction potential difference between NADH/NAD+ and quinone, rather than any inherent energy of the electrons themselves.

4- Line 98, Please specify the key residues that are missing in *B. subtilis* Ndh.

The key residues in question are detailed in the Results and in Extended Data Fig. 9. Given the manuscript is already lengthy, we do not think it necessary to duplicate this level of detail in the introduction.

5- Line 143, It is FAD not FAD+.

Done.

6- Line 153, Please include that DQ has a 10-hydrocarbon tail.

Done.

7- Line 213, Please specify the amount of MDA used here.

We have revised this figure legend to include the amount of MDA used.

8- Line 284. Please specify that 5 μM of NADH was added.

The relevant line now states that Ndh-Ncp was supplemented with 5x the molar concentration of NADH.

9- Line 299. The growth conditions of E. coli for the production of Ndh–Ncp ensured a 1:5 liquid:air ration (lines 673 to 675). Are anaerobic conditions established so E. coli can produce menaquinone?

No, they are not; we grow *E. coli* in aerobic conditions (1:5 liquid air) and in baffled flasks, so the menaquinone present likely represents low level background expression of menaquinone in *E. coli*.

10- Line 1291, extended data fig 7. Please remove the time-course transcriptomic profiling because the growth conditions must be different from the growth performed in the present study, in panel a) from extended data fig 1, the exponential phase is finished by 6 hour and the profiling starts at 8 hour.

We agree the growth conditions differ from those used in our study, and we state clearly that this transcriptomic profiling is drawn from an external database, not from our own experiments. We include it to illustrate differential expression across the three *B. subtilis* Ndh-2 paralogues (Ndh, YumB, YutJ), in support of the clade distinctions in our NDH-2 phylogenetic tree, not to represent growth rates or growth phase timing in our own system.

11- At least one reference is duplicated.

This has been amended.

References

Gyan, S., Y. Shiohira, I. Sato, M. Takeuchi and T. Sato (2006). "Regulatory Loop between Redox Sensing of the NADH/NAD+ Ratio by Rex (YdiH) and Oxidation of NADH by NADH Dehydrogenase Ndh in Bacillus subtilis." Journal of Bacteriology **188**(20): 7062-7071.
